# Design and evolution of the tetracycline repressor into sulfonylurea herbicide-responsive gene switches for field crops

**DOI:** 10.1038/s41467-026-73848-w

**Published:** 2026-06-08

**Authors:** Kevin E. McBride, Naga Kishore Kakani, Nina G. Bozhanova, Jin Fang, Keith Lowe, Christopher Leija, Brian McGonigle, Eric R. Schreiter, Flynn Hermanson, Mandy Chan, Maren Arling, Ning Wang, Hyeon-Je Cho, Christian Richey, George Hoerster, Jonathan S. Marvin, Brian Lenderts, Maxwell R. McReynolds, Craig Hastings, Alfredo Madrigal, S. Carl Falco, Michael W. Lassner, Ajith Anand, William Gordon-Kamm, Loren L. Looger

**Affiliations:** 1 Research and Development, Corteva Agriscience, Johnston, IA USA; 2 Department of Neurosciences, Howard Hughes Medical Institute, University of California, San Diego, La Jolla, CA USA; 3 Howard Hughes Medical Institute, Janelia Research Campus, Ashburn, VA USA

**Keywords:** Plant biotechnology, Protein design, X-ray crystallography, Synthetic biology

## Abstract

Chemically inducible expression systems enable transgene expression regulation in response to external small molecules. Tetracycline repressor (TetR)-based gene switches work in plants, but antibiotics are neither approved nor advisable for crop use. Here we report engineering of TetR mutants that respond to approved sulfonylurea (SU) herbicides instead of antibiotics. Designed variants show low-nanomolar EC_50_ values for ethametsulfuron-methyl (Es) or chlorsulfuron and tightly bind the Tet operator sequence, but only in the absence of corresponding SUs. Crystal structures of two repressors in complex with their respective SU ligands reveal extensive interactions explaining their strong binding. The Es repressor-based gene switch is introduced into tobacco, soybean, maize, rice, and *Arabidopsis*, and robust reporter gene activation is observed upon herbicide application. Addition of a repressor-regulated siRNA targeting the repressor transcript increases the magnitude and spatial distribution of the response following herbicide treatment and results in a partially bistable gene switch. The SU repressors also function well in mammalian cell culture and may enable regulation of additional genes in conjunction with TetR.

## Abstract (web summary)

Practical chemically inducible expression systems for crop plants remain lacking. Here, the authors report the design and evolution of the tetracycline repressor into sulfonylurea herbicide-responsive gene switches and show their application in multiple crops as well as mammalian cell.

## Introduction

Chemically-inducible expression tools are valuable academically for studying gene function and regulation, and practically for controlling phenotypes and processes in many organisms^1^. Numerous inducible gene expression systems have been discovered and developed through the generation of inducible chimeric transactivators and extensive protein-engineering efforts to expand the ligand specificities of the allosteric transcription factors^2–5^. In plants, these efforts have resulted in the development of several widely used inducible systems, including dexamethasone-^6^, estrogen-^7^, and ethanol-inducible^8^ systems. These systems provide strong and temporally controllable gene activation in laboratory settings. However, their inducers are not suitable for field deployment due to regulatory restrictions (dexamethasone, estrogen) and challenges related to volatility, dosing control, and pleiotropic effects (ethanol).

Important features of a field-compatible system include low background, high magnitude of inducer-evoked response, and practicality of inducer application (i.e., rapid penetration and movement to target tissues/cells, lack of degradation by target plant, neutrality to growth and development, safety, photostability, and registration for field use). Only two existing plant-specific induction systems employ approved agrochemicals as inducer: ecdysone receptor (EcR)-based systems^9–11^ and ABA receptor PYR1-based systems^12,13^.

In the case of ABA receptor-based systems, *in planta* reporter gene activation was shown only in response to azinphos-ethyl—to demonstrate the system’s potential for environmental monitoring of this toxic compound. By contrast, the agriculturally relevant candidate from that paper—mandipropamid—was shown only to induce expression of *RD29B*, a native ABA-responsive gene, with no synthetic reporter gene induction demonstrated.

EcR-based systems appeared highly promising in the early 2000s during the period of their active development^9–11^. However, they have never become widely adopted in plants for several reasons. The primary limitation is the poor bioavailability of approved ecdysone analogs (such as tebufenozide and methoxyfenozide) within plant tissue^14^, which makes induction inefficient, particularly when applied by spraying. In addition, many potent EcR agonists have been reported to occur naturally in plants^15^ (e.g., phytoecdysteroids like ecdysterone, a.k.a. 20-hydroxyecdysone), leading to undesirably high basal expression levels and diminished responses to exogenous inducer. These shortcomings have prevented EcR-based systems from being widely adopted for plant use. Thus, there appear to be no readily deployable agrochemical-inducible expression systems.

**Fig. 1:**
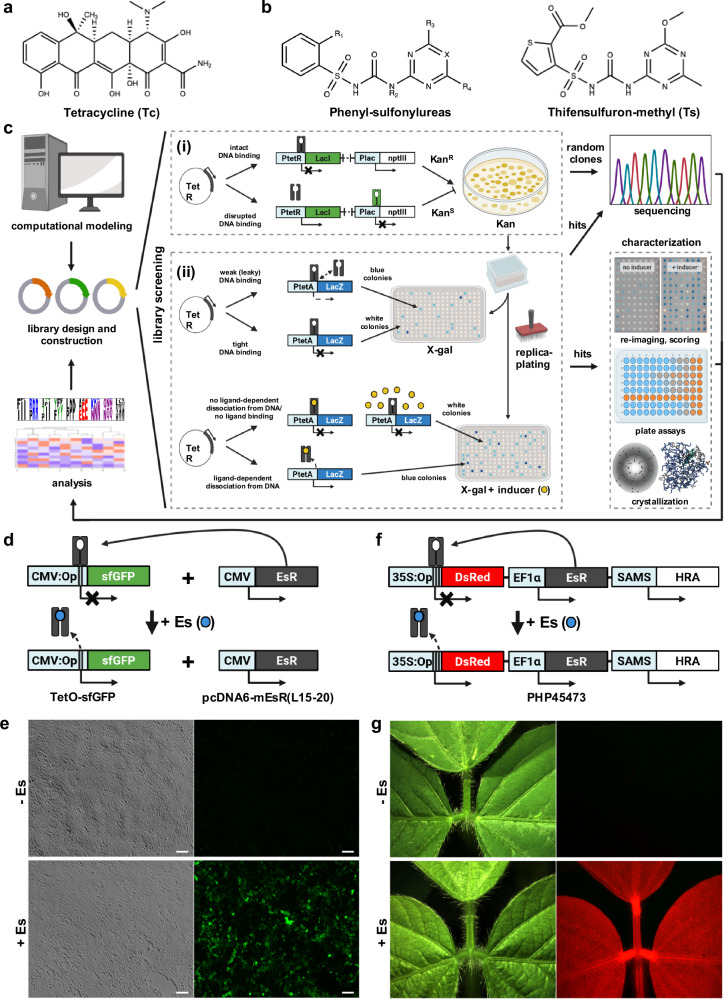
Development of sulfonylurea (SU) repressors. **a** Structure of tetracycline (Tc). **b** Structures of the sulfonylurea herbicides. Left: chlorsulfuron (Cs, R_1_ = Cl, R_2_ = ··, R_3_ = OMe, R_4_ = Me, X = N); tribenuron-methyl (Tb, R_1_ = CO_2_Me, R_2_ = Me, R_3_ = OMe, R_4_ = Me, X = N); metsulfuron-methyl (Ms, R_1_ = CO_2_Me, R_2_ = ··, R_3_ = OMe, R_4_ = Me, X = N); ethametsulfuron-methyl (Es, R_1_ = CO_2_Me, R_2_ = ··, R_3_ = OEt, R_4_ = NHMe, X = N); chlorimuron-ethyl (Ci, R_1_ = CO_2_Et, R_2_ = ··, R_3_ = OMe, R_4_ = Cl, X = CH); rimsulfuron (Rs, R_1_ = SO_2_Et, R_2_ = ··, R_3_ = OMe, R_4_ = OMe, X = CH); nicosulfuron (Ns, R_1_ = CONMe_2_, R_2_ = ··, R_3_ = OMe, R_4_ = OMe, X = CH); sulfometuron-methyl (Sm, R_1_ = CO_2_Me, R_2_ = ··, R_3_ = Me, R_4_ = Me, X = CH). Right: thifensulfuron-methyl (Ts). **c** Repressor evolution strategy. The initial library was inspired by computational modeling. Clones were (i) pre-screened for intact DNA-binding activity on kanamycin (Kan)-containing plates, then (ii) replica-plated on X-gal plates to screen for low leakiness (white colonies) in the absence of inducer, and high activation signal (blue colonies) in its presence. Hits were replated and reanalyzed. Hits and random clones were sequenced. Sequencing and rescreening results were used to guide the design of the next library. Created in BioRender. Looger, L. (2026) https://BioRender.com/g9i9440. **d** Design of vectors TetO-sfGFP and pcDNA6-mEsR(L15-20). Light blue: promoters. Green: sfGFP. Gray: repressor. Dark blue circle: Es. Top: without Es, EsR occupies the *CMV-Op* promoter, blocking sfGFP expression. Bottom: upon binding Es, EsR dissociates from the *CMV-Op* promoter, allowing sfGFP expression. Created in BioRender. Looger, L. (2026) https://BioRender.com/g9i9440. **e** SfGFP expression in HEK293 cells transiently co-transfected with TetO-sfGFP and pcDNA6-mEsR(L15-20) plasmids following treatment with DMSO (top) or Es (bottom). Images (bright field, left; EGFP filter set, right) were taken with identical exposure times and camera settings. Scale bar 100 μm. Shown are representative images of four independent experiments. **f** Design of vector PHP45473. Light blue: promoters. Red: DsRed-Express. Gray: repressor. White: HRA selection marker. Dark blue circle: Es. Top: without Es, EsR occupies the *35S-Op* promoter, blocking DsRed-Express expression. Bottom: upon binding Es, EsR dissociates from the *35S-Op* promoter, allowing DsRed-Express expression. Created in BioRender. Looger, L. (2026) https://BioRender.com/g9i9440. **g** DsRed-Express expression in soybean harboring vector PHP45473 after root soak with water (top) or 250 μg of Es (bottom). Images (bright field, left; DsRed filter set, right) were taken with identical exposure times and camera settings.

The tetracycline repressor (TetR) from bacteria is highly sensitive and widely used as the basis of gene induction systems. It has been used for human gene therapy studies and diverse applications in transgenic animals^16,17^. While wild-type TetR (wtTetR)-controlled gene expression has been successfully adapted for use in plants^18–20^, its applications are limited to the lab, since the ligands are antibiotics (risking development of resistance), light-sensitive, and not approved for use on field crops. Although wtTetR has been evolved to respond to two tetracycline (Tc, Fig. 1a) analogs that it did not previously recognize^21,22^, these changes were quite subtle. Radical manipulation to accommodate much more distant chemistry has been reported neither for TetR nor for any other allosteric transcription factors.

We sought to mold TetR to recognize sulfonylurea (SU) herbicides (Fig. 1b), which inhibit the branched-chain amino acid biosynthetic enzyme acetolactate synthase (ALS)^23,24^ in plants. These compounds have excellent plant uptake (foliar and root) and distribution properties, low toxicity to other organisms, and a molecular size similar to Tc. No known analogs have been reported to be produced by organisms, minimizing the chances of crosstalk from environmental factors. Furthermore, high-resistance alleles (HRAs) of ALS exist that render host plants immune to SUs^25^, ensuring that the genetically modified crops are not themselves affected by the treatment. In many situations, these SUs would be applied anyway to kill weeds; thus, we sought to repurpose chemicals already being applied for a second purpose—i.e., turning on or off target genes.

In this work, we select thifensulfuron-methyl (Ts, Harmony®), an SU registered for use on corn and soybeans (Supplementary Table 1), as a starting compound. Using a variant of a computational interface design algorithm^26^, we identify potential TetR mutations to convert the Tc-binding pocket into a Ts-binding site. Through iterative rounds of DNA shuffling^27,28^ and selection of TetR mutants for SU-dependent reporter gene expression and low SU-free background activity, we identify repressor variants responding to different SU molecules but not to Tc. The final round produces SU repressors (SURs) with genetic switch properties similar to TetR. Crystal structure analysis, biochemical characterization, and cell culture assays confirm the screening results and lead to the development of the SU chemical switch technology in multiple plant species encompassing field crops and lab model species. The technologies developed here could enable activation and temporal control of various genetic traits with SU herbicide application, enabling single-season activation or transient induction of transgenes that under constitutive expression would negatively affect seed formation, viability, or yield.

## Results

### Overall strategy

To develop SU-responsive repressors, we employed a strategy of iterative (i) library design and construction, (ii) screening and selection in bacteria for high ligand-dependent induction and low ligand-free background expression (“leakiness”), (iii) hit sequencing, and (iv) determination of hit trends (Fig. 1c). In later rounds, biochemical characterization of individual hits was performed, including more precise binding measurements and crystal structure determination. We began modeling and testing with a set of nine SU herbicides with different physicochemical properties and *in planta* applications (Supplementary Table 1, Fig. 1b), to maximize chances of finding initial hits. After the first few rounds of design and screening, we selected two SUs (ethametsulfuron-methyl, Es, Muster®; chlorsulfuron, Cs, Glean®) for subsequent optimization.

### Bacterial screening and selection system

We constructed a set of plasmids with ampicillin resistance to express our libraries of TetR variants. Plasmids carried the *tetR* gene under control of the *P*_*BAD*_ promoter^29^. SacI and AscI restriction sites flanked the TetR ligand-binding domain (LBD, residues 50-207) to easily insert libraries of mutated LBDs.

We also designed and created a strain of *Escherichia coli*, denoted KM3, for the selection of clones with an active repressor, followed by assessing their derepression properties in the presence of the tested ligand based on the principles of Wissmann et al.^30^ (Methods). KM3 contains two genomically integrated expression modules. The first encodes a kanamycin resistance gene, *nptIII*, under the control of lac promoter and was inserted in place of the *tolC* locus. *TolC* encodes an efflux pump that is known to expel a wide range of small molecules^31^, and its removal should allow for a more sensitive screening system. The second expression module encodes two expression cassettes: one encoding *lacI* and the other—*lacZ*, both controlled by divergent Tet promoters. In the absence of a functional Tet repressor, *lacI* is transcribed, and the produced lac repressor inhibits expression of *nptIII*, making cells susceptible to kanamycin. Transforming a repressor library into the KM3 strain and plating it onto inducer-free plates with kanamycin and carbenicillin thus allows pre-selection for transformed colonies encoding TetR mutants with acceptable repressor activity (Fig. 1c(i)). *LacZ*, which encodes β-galactosidase (β-gal), is expressed if the Tet repressor does not bind the Tet operon too tightly or if it dissociates from the promoter in the presence of the ligand. This allows further screening of repressor properties in bacterial colonies based on their β-gal activity using the chromogenic substrate 5-bromo-4-chloro-3-indolyl-β-D-galactopyranoside (X-gal). Colonies from kanamycin and carbenicillin plates can be replica-plated onto plates containing X-gal with or without inducer. Colonies with a highly active repressor would be white on X-gal plates without an inducer; those with a leaky repressor would turn blue. On replica plates with X-gal and inducer, the blue colony color would deepen for colonies with repressors that dissociated from the promoter upon ligand binding; color would be unchanged for those with repressors unresponsive to the ligand (Fig. 1c(ii)). Color intensity should scale with the degree of leakiness and/or derepression. This second screening step can be made increasingly stringent over the course of design rounds by lowering inducer concentration and decreasing repressor expression level. To more precisely characterize repressor properties, β-gal activity can also be measured using the fluorogenic substrate 4-methylumbelliferyl-β-D-galactopyranoside (MUG).

### First-round library design and screening

Tetracyclines (Tc and its derivative anhydrotetracycline, Atc) and SU herbicides are very different molecules with respect to overall shape and functional groups. Tetracyclines are internally rigid and fairly flat, with one highly-hydrogen-bonding-prone face rich in hydroxyls and ketones (Fig. 1a). SU herbicides are more flexible and aromatic, with a core SU moiety typically bearing a substituted phenyl, pyridine, or thiophene on one side and a substituted pyrimidine or 1,3,5-triazine on the other (Fig. 1b). We selected thifensulfuron-methyl (Ts, Harmony®) as our first target given its relatively small size and widespread use. Despite the differences, tetracyclines and Ts are somewhat similar in terms of molecular weight (MW: Atc, 426.4; Ts, 387 g/mol), dimensions (~10 Å × 5 Å), hydrophobicity (logP: Atc, 1.2; Ts, 2.0), and isoelectric point (pI: Atc, 5.9; Ts, 3.2). Furthermore, manual positioning of Ts in the TetR binding pocket produced no large clashes with the protein backbone. Thus, we reasoned that (perhaps heavily mutated) variants of wtTetR should be capable of binding and responding to SUs.

The crystal structure of the class D TetR from *E. coli* in complex with 7-chlortetracycline (Protein Data Bank (PDB) ID 1DU7, Supplementary Fig. 1a) was used as the scaffold for the design of TetR mutants capable of binding to and being activated by SUs. Residues constituting a potential SU binding pocket were identified by inspection (Supplementary Fig. 1b). These 17 positions were mutated in silico to all amino acids but cysteine and evaluated for compatibility with the TetR backbone, producing an initial amino acid library. In parallel, atomic models of 144 Ts conformers were created and placed at nodes of a lattice centered at the center of mass of the 7-chlortetracycline in 1DU7 (Methods).

These Ts placements were scored by assessing clashes with the scaffold and possible interactions with the side-chains of the residues from the previously created amino acid library at the 17 variable positions. Out of all possible ligand placements, 1811 were deemed possibly compatible with the protein scaffold and partitioned into 20 clusters (Supplementary Fig. 2a). The median pose was taken as representative of each cluster (Supplementary Fig. 2b), and full binding pocket sequence redesign was again performed, this time in the context of each placed ligand. The amino acid diversity of top designs was merged into an amino acid library representative of all clusters (Methods, Supplementary Fig. 3, column 1).

Conversion of the computationally designed diversity into nucleic acid libraries for testing presented multiple practical hurdles. In most cases, the desired amino acid diversity at individual positions was not uniquely encodable by a single degenerate codon—in most cases, many codons would be required—increasing the number of oligos needed. This problem was further exacerbated by regions where multiple residues would need to be encoded on the same oligo due to their proximity (e.g., positions 134, 135, 138, and 139), producing combinatorial complexity. To make the mutagenesis feasible, the library was expanded to encode a slightly enlarged set of amino acids at some positions (Supplementary Fig. 3, column 2, Supplementary Data 1), resulting in a much larger (~3 × 10^19^) theoretical sequence diversity—but one that could be readily assembled in the lab.

This library, denoted L1, was assembled by PCR, cloned into the pVER7314 vector, and transformed into the KM3 *E. coli* strain. The library was first plated onto Luria Broth (LB) plates containing carbenicillin (to select transformants) and kanamycin (to select for intact repressor activity). After sequencing ~100 random colonies to verify library quality and diversity (Supplementary Fig. 3, column 3), ~20,000 randomly picked clones were screened for Ts-induced derepression and preserved repressor activity by replica-plating onto X-gal-containing minimal media (M9) agar plates with or without 20 μg/mL (~52 μM) Ts. While most colonies showed high leakiness and/or weak derepression by Ts, we discovered 40 with relatively low leakiness and noticeable response to Ts. These hits were rearrayed and assayed for β-gal activity with a panel of nine SU herbicides (Fig. 1b) and the TetR inducer Atc to confirm their response to Ts and assess their SU-binding specificity (Supplementary Fig. 4a). Surprisingly, a number of other SUs (chlorsulfuron, Cs; chlorimuron-ethyl, Ci; ethametsulfuron-methyl, Es; and sulfometuron, Sm) induced much stronger responses than Ts in the most active clones. We selected 11 clones with low leakiness and strong activation by one or just a few compounds and retested them against the same set of ligands using a more sensitive fluorescence-based β-gal activity assay (Supplementary Fig. 4b). The largest number of clones responded to Ci, while Es elicited the strongest and most selective response from a single clone, L1-9. WtTetR exhibited no response to any SU, and no hits were activated by 4 μM Atc.

Analysis of these hit sequences (Supplementary Fig. 3) revealed significant enrichment at several library positions compared to randomly picked colonies. Enrichment was broadly consistent with the computational model of Ts binding. For instance, W was favored at 4 positions (100, 105, 134, 147), two of which had W among the designed diversity. R was favored at 3 positions (135, 138, 151), all of which had either R or K (positively charged residues) suggested by computational design. Negatively charged residues (D, E) were strongly selected against at all positions.

Highly sensitive Es- and Cs-responsive repressors (EsRs and CsRs) were then evolved throughout five additional rounds of library design and screening, resulting in a several-thousand-fold decrease of EC_50_ over round-one hit L1-9 (Supplementary Figs. 5–13, Table 1, Supplementary Note 1, Supplementary Data 2–19).

**Table 1:** Sensitivity of hits from six rounds of library design and screening to different SUs

	Es EC_50_, ng/mL (nM)	Cs EC_50_, ng/mL (nM)	Atc^a^ EC_50_, ng/mL (nM)	Ci^b^ EC_50_, ng/mL (nM)	Ms^b^ EC_50_, ng/mL (nM)	Ts^b^ EC_50_, ng/mL (nM)
L1-9	>10,000 (>24,000)	>10,000 (>28,000)	n/d	n/a	n/a	n/a
L4-118	>10,000 (>24,000)	>10,000 (>28,000)	n/d	n/a	n/a	n/a
L7-A11	71 (~173)	4945 (~13,820)	n/d	n/a	n/a	n/a
L7-D01	214 (~521)	>10,000 (>28,000)	n/d	n/a	n/a	n/a
L10-B07	22 (~54)	2761 (~7717)	n/d	n/a	n/a	n/a
L10-B08	16 (~39)	553 (~1546)	n/d	n/a	n/a	n/a
L11-C06	25 (~61)	>10,000 (>28,000)	n/d	n/a	n/a	n/a
L12-11	11 (~27)	995 (~2781)	n/d	65 (~157)	1298 (~3403)	>10,000 (>26,000)
L13-9	11 (~27)	>10,000 (>28,000)	n/d	172 (~415)	>10,000 (>26,000)	>10,000 (>26,000)
L13-23	54 (~132)	>10,000 (>28,000)	n/d	799 (~1926)	>10,000 (>26,000)	n/d
L15-20	12 (~29)	1847 (~5162)	n/d	72 (~174)	1648 (~4321)	>10,000 (>26,000)
wtTetR	n/d	n/d	3 (~7)	n/a	n/a	n/a
L2-14	n/d	>10,000 (>28,000)	n/d	n/a	n/a	n/a
L6-1B03	n/d	>10,000 (>28,000)	n/d	n/a	n/a	n/a
L6-4D10	n/d	>10,000 (>28,000)	n/d	n/a	n/a	n/a
L8-3F01	>10,000 (>24,000)	1707 (~4771)	n/d	n/a	n/a	n/a
CsL3-B9	>10,000 (>24,000)	355 (~992)	n/d	n/a	n/a	n/a
CsL3-B11	539 (~1313)	29 (~81)	n/d	n/a	n/a	n/a
CsL4.2-15	933 (~2273)	14 (~39)	n/d	131 (~316)	289 (~578)	447 (~1154)
CsL4.2-20	262 (~638)	13 (~36)	n/d	80 (~193)	163 (427)	308 (~795)

L1—1st round; EsR libraries: L4—2nd round, L7—3rd round, L10 and L11—4th round, L12 and L13—5th round, and L15—6th round; CsR libraries: L2—2nd round, L6—3rd round, L8—4th round, CsL3—5th round, and CsL4.2—6th round. Curves are shown in Supplementary Figs. 8–13. *n* = 3 for Es and Cs.

*n/d* no detectable binding, *n/a* not tested.

^a^*n* = 1 for all variants except for wtTetR and L1–9.

^b^*n* = 1 for all variants.

### Crystal structure analysis

We solved the crystal structures of the highly active round four EsR hit L11-C06 in complex with Es, the round three EsR hit L7-D01 in the apo form, and the round six CsR hit CsL4.2-20, both bound to Cs and apo, at 1.7–2.85 Å resolution (Supplementary Table 2, Supplementary Fig. 14).

**Fig. 2:**
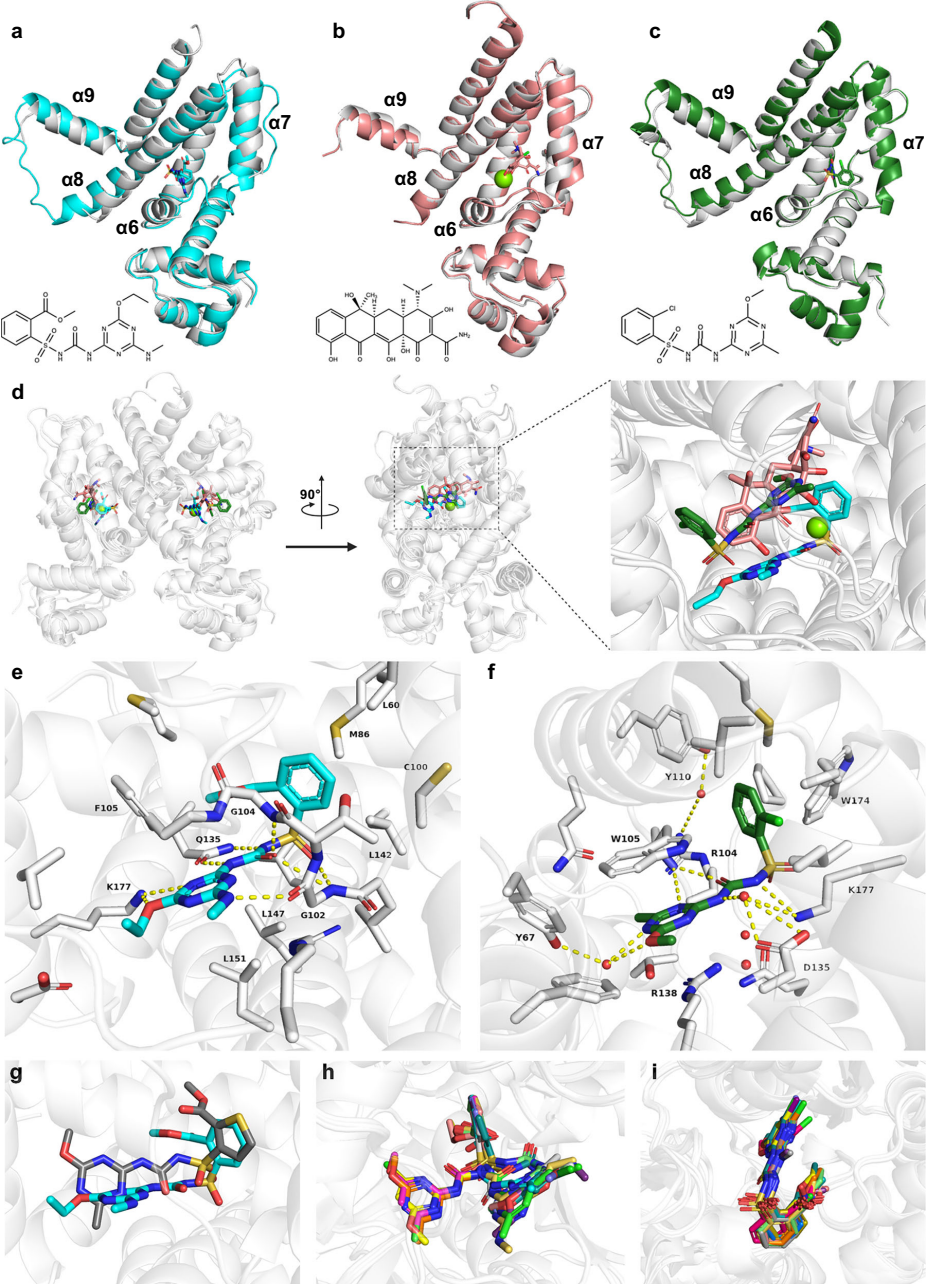
Structural characterization of SURs. **a**–**c** Overlaid apo- (white) and ligand-bound (colored) monomers of EsR (**a**, this study), wtTetR (**b**, PDB IDs 1A6I and 2TCT), and CsR (**c**, this study). Proteins are shown as cartoons, ligands are shown as sticks, Mg^2+^ is shown as a green sphere, and discussed helixes are labeled. Chemical structures of the corresponding ligands are shown at the bottom left. **d** Overlaid class D wtTetR-Tc (PDB ID 2TRT), EsR L11-C06-Es, and CsR L4.2-20-Cs complexes: overall ligand position in the proteins and close-up of the ligand-binding cavity. Proteins are shown as cartoons, ligands are shown as sticks, Mg^2+^ is shown as a green sphere. Salmon—Tc, cyan—Es, green—Cs. **e**, **f** Details of protein-ligand interactions in the EsR L11-C06-Es (**e**) and CsR L4.2-20-Cs (**f**) complexes. Proteins are shown as white cartoons, ligands are shown as colored sticks, protein residues forming the ligand-binding cavity are shown as white sticks, key interacting residues are labeled, water molecules are shown as red spheres, and hydrogen bonds are shown as dashed yellow lines. **g** The relative orientation of the median cluster 9 Ts pose (gray) and Es (cyan) in the EsR L11-C06-Es structure. **h**, **i** Overlay of the available SulE-SU (**h**) and AHAS-SU (**i**) complexes. Proteins are shown as white cartoons, ligands are shown as colored sticks.

Despite a substantial number of incorporated mutations (22–26 in a 207 amino acid long protein), all obtained SUR structures are very similar to the wild-type class B TetR (PDB ID 4AC0), with RMSDs of 1.1–1.8 Å for all common C_α_ atoms in the functional dimer. Solving the structures of both the ligand-bound and apo versions of SURs (although of slightly different mutants in the case of EsR) allowed us to investigate ligand-induced conformational changes. Alignment of apo EsR L7-D01 with Es-bound EsR L11-C06 showed structural perturbations similar to those seen upon Tc binding to wtTetR^32^. The most significant conformational changes include unfolding of the C-terminus of α6, perturbation of the loop connecting helix α6 to α7 (although a part of this loop was unresolved in EsR L7-D01), and displacement of α7, α9, and the N-terminus of α8 (Fig. 2a), suggesting a mechanism of ligand-induced derepression analogous to wild-type (Fig. 2b). Interestingly, the apo and Cs-bound structures of CsR CsL4.2-20 overlap almost perfectly, except for the N-terminus of α8, α9, and the loop connecting them (Fig. 2c). More studies would be needed to determine whether the Cs-mediated derepression indeed utilizes a different mechanism, or whether this is a crystallization artifact.

The binding modes of Tc (in class D TetR, PDB ID 2TRT), Es, and Cs to their respective repressors are quite different, despite the ligands occupying the same binding pocket and the high structural similarity of SUs (Fig. 2d). Neither SU requires Mg^2+^ ions for ligand binding, unlike wtTetR binding Mg^2+^-Tc complex.

In the EsR-Es complex, Es is stabilized by numerous hydrogen bonds: G102 H-bonds to the urea carbonyl and the triazine ring methylamine substituent, Q135 forms two H-bonds with both urea nitrogens of the Es, G104 H-bonds with the urea carbonyl, and K177 interacts either with the triazine ring nitrogen or its ethoxy substituent. The ligand-protein interface also features a methyl-pi interaction (M86 with phenyl ring) and many hydrophobic packing interactions, including L60, C100, F105, L142, L147, L151, etc. (Fig. 2e). Some additional water-mediated hydrogen bonds might not be seen due to the resolution of the structure. Many of the residues interacting with Es (C100, G104, F105, Q135, L147, L151, K177) were strongly selected during library screening (Supplementary Fig. 5).

The pose of Cs bound in the pocket of CsR L4.2-20 is essentially coplanar with Es bound to EsR L11-C06, although Cs is translated ~3 Å and rotated ~180° relative to Es (Fig. 2d). Its position much more closely matches the position of Tc in TetR than does Es in EsR L11-C06. As with the other complexes, Cs forms many diverse interactions with the protein (Fig. 2f). The Cs triazine ring is involved in pi-pi stacking interactions with W105 from the α6-α7 loop, which moves out substantially relative to the structures of wtTetR and EsR, stabilized by the water-mediated R104-Y110 interaction, to allow more space for the Cs. The opposite face of the triazine ring makes a strong cation-pi interaction with R138. The Cs phenyl ring also makes extensive contacts, including herringbone interactions with W105 and W174 from the 2nd monomer. The Cs also forms many hydrogen bonds with the protein: the backbone amide of W105 H-bonds with the urea carbonyl and a triazine ring nitrogen, K177 and D135 make direct and water-mediated H-bonds with the sulfonylurea, and Y67 makes a water-mediated H-bond to the triazine ring nitrogen and its methoxy substituent. Almost all of these directly interacting side chains (Y67, W105, R138, W174, K177) were enriched in Cs responders from the early rounds of directed evolution (Supplementary Fig. 6).

We also compared the positions of SUs in our experimentally determined crystal structures with the ensemble of Ts placements obtained from the initial computational design that informed the first-round library diversity. While most docked Ts placements differed significantly, with the docked ligand sitting deeper in the binding pocket than experimentally observed (Supplementary Fig. 15), the median pose from cluster 9 is similarly oriented and has ~2 Å RMSD with Es in the EsR L11-C06:Es structure (1.8 Å if calculated over 7 heavy atoms of the sulfonylurea group and the first atoms of each of the two substituents; 2.8 Å if calculated over all 22 similarly placed heavy atoms; Fig. 2g). Given that Tc also sits deeper in the binding pocket than Es and Cs (hence, the region computationally screened for Ts placement was centered further away from the actual Es and Cs positions), the chemical dissimilarity of Ts from other SUs (Ts contains a thiophene ring, while the other SUs screened contain a phenyl ring), the application of a fixed-backbone design protocol to a protein as flexible as TetR, and the number of additional directed evolution cycles between our initial design and the crystallized proteins, it is unsurprising that the initial models do not perfectly align with the final structures.

High-resolution structures are available in the PDB for several other proteins bound to SU ligands, including the herbicide-detoxifying esterase SulE^33,34^ and the target of SU herbicides, acetohydroxyacid synthase (AHAS, also known as ALS)^23,35–39^. In the SulE structures, the phenyl ring of the SU ligand inserts into a hydrophobic pocket, where it heavily interacts with the protein. The ligand is further stabilized through hydrogen bonds to the SU oxygen and nitrogen atoms, while the heterocyclic moiety is located outside the pocket and makes only a few contacts with the protein (Fig. 2h). Consistent with interactions involving just half of the SU molecule, the K_D_s of SulE binding to SUs are in the μM range, and the SUs adopt diverse conformations.

Conversely, the position and conformation of the bound SUs in all AHAS structures are remarkably similar (Fig. 2i). This is achieved through a combination of shape complementarity (a narrow channel leading to the active site occupied by the SU bridge) and several highly conserved interactions (R forming hydrogen bonds with a nitrogen atom of the heterocyclic ring and the carbonyl oxygen of the SU bridge; W involved in pi-pi stacking with the SU heterocyclic ring; several hydrophobic residues forming a hydrophobic pocket). Accordingly, SUs bind to AHAS with K_D_s in the mid-low nM range.

Our SUR:SU complexes display binding features intermediate between these two extremes. They form numerous diverse interactions with their ligands, leading to affinities in the nM range. However, because of the naturally wide TetR binding cavity and the dramatically different mutagenic paths taken by EsR and CsR during directed evolution, it proved possible to stabilize SUs in quite different positions and orientations.

### Validation of SUR function in mammalian cells

We first tested the engineered SURs using transient expression in mammalian cells. We cloned two EsR constructs (L13-9 and L15-20) and two CsR constructs (CsL4.2-15 and CsL4.2-20) in place of TetR in the pcDNA6/TR vector of the T-REx kit (Thermo-Fisher), and superfolder GFP (sfGFP) into the pcDNA4/TO reporter vector (Fig. 1d). The pcDNA6/Repressor (one of the two EsRs or two CsRs, or the TetR parental vector) and pcDNA4/TO-sfGFP vectors were co-transfected into HEK293 cells. Tc, Es, Cs, or DMSO (carrier solution—negative control) were added to the cells the next day. The cells were allowed to express for 24 h after induction, then imaged (Fig. 1e) and quantified for green fluorescence by flow cytometry (Supplementary Fig. 16). TetR responded strongly to Tc but not to either SU ligand; EsRs L13-9 and L15-20 responded strongly to Es, almost negligibly to Cs, but not to Tc; CsR L4.2-15 responded strongly to Cs, weakly to Es, but not Tc; CsR L4.2-20 responded strongly to both Es and Cs, but not Tc. Without a repressor, the sfGFP signal was equivalent in all cells. Thus, the designed repressors function as intended in mammalian cells, and the SU compounds are cell-permeable and do not show any obvious toxicity at the used concentrations.

### Validation of SUR function in transgenic plants

We then proceeded to test the SURs in our target system—plants. For strong, TetR-dependent expression of a reporter gene, we used the previously described “Triple-Op” promoter^20^ (referred to further as *35S-Op*)—a derivative of the potent, constitutive cauliflower mosaic virus *35S* promoter^40^ with three *TetO* sequences inserted around the TATA box. In the presence of an active repressor, reporter transcription should be blocked; in the presence of the ligand, the dissociation of the repressor is expected to result in rapid and robust expression of the reporter.

Soybean (*Glycine max* [L.] Merr. cv. Jack) embryogenic suspension cultures were transformed via particle bombardment with vector PHP45473, which includes a repressible *35S-Op*-driven DsRed-Express fluorescent marker^41^, an EsR L15-20 under the strong constitutive soybean *EF1a2* promoter^42^, and a selectable marker, the HRA, driven by the promoter from the soybean S-adenosylmethionine synthase gene (*SAMS*) (Fig. 1f, Supplementary Table 3). HRA is a double mutant (P184A, W561L)^25^ of soybean AHAS and provides resistance to both SU and imidazolinone (e.g., imazapyr) herbicides. This vector design allowed for the selection of transgenic soy plants on Cs following DNA transformation. Since Cs also acts as an inducer of this EsR variant (albeit not as efficiently as Es), transgenic soy calli were double-confirmed based on a DsRed-Express^+^ phenotype. Transgenic plants containing a single copy of the integrated vector (as determined by qPCR) were isolated.

Homozygous progeny of one of the clonal events were analyzed in the herbicide soil soaking experiment. Soil soak with Muster® (active ingredient - Es) led to complete and widespread derepression, with DsRed-Express expression increasing from near-background levels in water-treated controls to a strong signal in treated leaves (Fig. 1g, Supplementary Fig. 17). This confirmed the feasibility of stable integration and functional SU-dependent derepression of SUR-controlled genes in intact plants.

### Deployment of the SURs in monocots

**Fig. 3:**
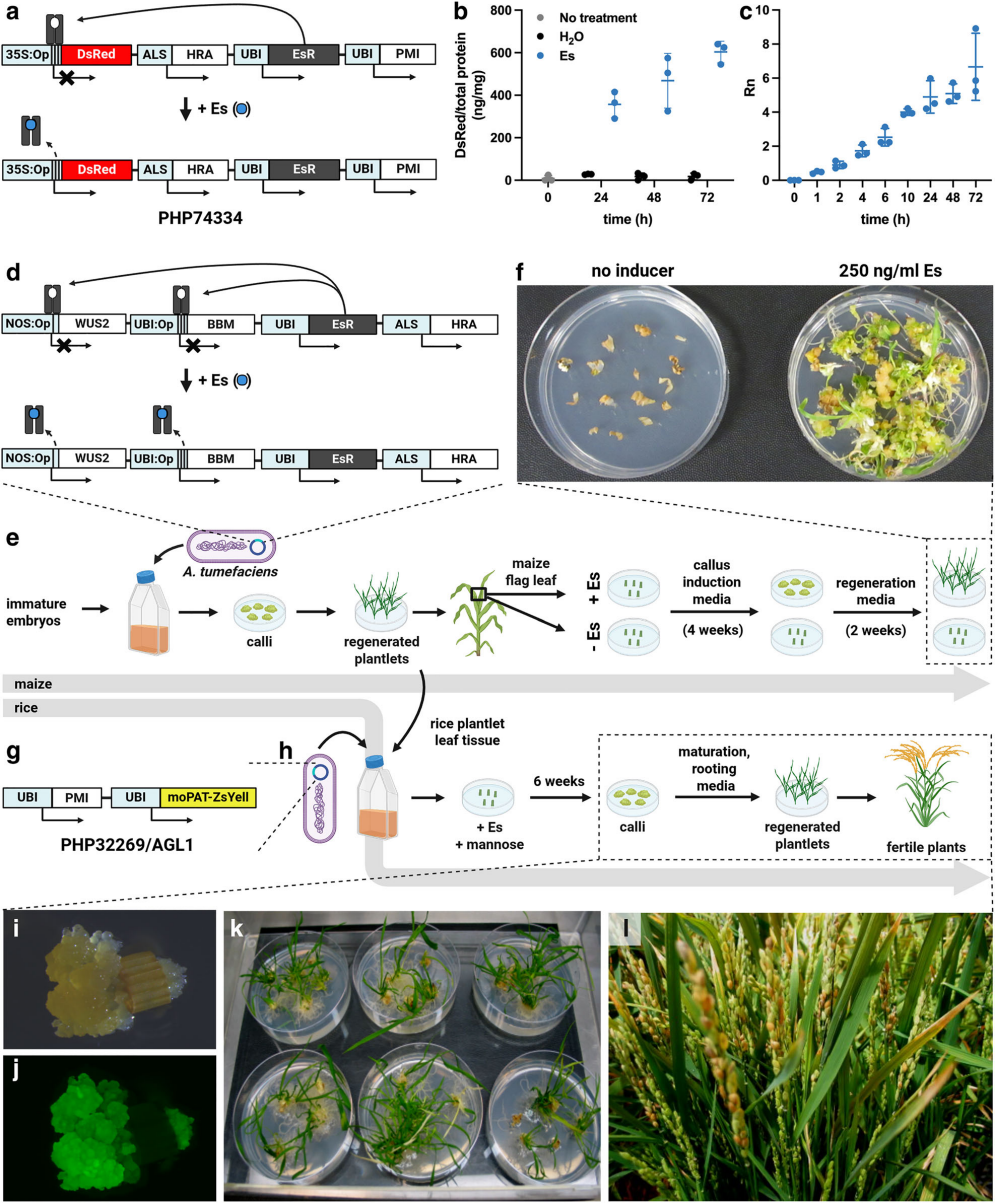
Deployment of the SURs in monocots. **a**–**c** Induction of DsRed-Express expression in maize leaf discs following Muster® (active ingredient - Es) treatment. **a** Design of vector PHP74334. Light blue: promoters. Red: DsRed-Express reporter gene. Gray: repressor. White: HRA selection marker. Dark blue circle: Es ligand. Top: without Es, EsR occupies the *35S-Op* promoter, blocking fluorescent protein expression. Bottom: upon binding Es, EsR dissociates from the *35S-Op* promoter, allowing DsRed-Express expression. Created in BioRender. Looger, L. (2026) https://BioRender.com/i97wi2p. **b** Protein level determined with biolayer interferometry in water- and Es-treated transgenic plant samples. *n* = 3, shown are the mean and std. dev. **c** DsRed-Express transcript levels determined by qRT-PCR relative to a constitutive reference gene. *n* = 3, shown are the mean and std. dev. **d**–**f** Inducible embryogenesis in maize. **d** Vector design. Light blue: promoters. Gray: repressor. White: HRA selection marker. Dark blue circle: Es ligand. Top: without Es, EsR occupies the *NOS-Op* and *UBI-Op* promoters, blocking the expression of *Wus2* and *Bbm*. Bottom: upon binding Es, EsR dissociates from *NOS-Op* and *UBI-Op* promoters, allowing the expression of *Wus2* and *Bbm*. Created in BioRender. Looger, L. (2026) https://BioRender.com/i97wi2p. **e** Maize experimental pipeline: vector PHP63015 was used to generate transgenic plants that were grown to just before the emergence of the tassel. The flag leaf was isolated, cut, and placed on callus induction media with or without Es. Created in BioRender. Looger, L. (2026) https://BioRender.com/i97wi2p. **f** Flag leaf fragments grown with (right) or without (left) Es. **g**–**l** Deployment of SURs to enable rice leaf transformation. **g** Rice experimental pipeline: vector PHP60850 was used to generate transgenic plantlets. Leaf tissue from PHP60850-carrying T0 plantlets was cut into segments and infected with *A. tumefaciens* harboring vector PHP32269. Leaf tissue was incubated on mannose selection media with Es, calli were transferred to maturation media, then rooting media, without Es, and the resulting plantlets were transferred to soil. Created in BioRender. Looger, L. (2026) https://BioRender.com/i97wi2p. **h** Vector PHP32269 design. Light blue: promoters. White: constitutively expressed selectable marker phosphomannose isomerase (*PMI*). Yellow: constitutively expressed maize-optimized phosphinothricin acetyltransferase (*moPAT*) structural gene fused to the yellow fluorescent protein ZsYellow. Created in BioRender. Looger, L. (2026) https://BioRender.com/i97wi2p. **i**, **j** Calli growing on mannose- and Es-containing medium exhibited yellow fluorescence (**j**). **k** Rice plantlets growing on maturation and germination medium. **l** Apparently healthy, fertile, transgenic rice plants in the greenhouse. Source data are provided as a Source data file.

We next tested the designed repressors in maize (*Zea mays*), a monocot. We constructed vector PHP74334 based on vector PHP45473 described above with several monocot-specific adaptations: the repressor was driven by a strong, constitutive maize ubiquitin promoter (*ZmUbi*_*pro*_); the HRA resistance allele was created from maize ALS^43^ and was driven by the *Sorghum bicolor* ALS promoter (*SbAls*_*pro*_); the selectable marker phosphomannose isomerase (PMI) was added, facilitating growth on mannose as a carbon source; and finally, the maize alcohol dehydrogenase 1 (*Adh1*) intron was placed downstream of the 5′-UTR to enable high-level expression in monocots^44^ (Fig. 3a, Supplementary Table 3).

Pioneer maize inbred PH184C was transformed using *Agrobacterium* strain LBA4404 THY- carrying the plasmid PHP74334. Transformants were selected on mannose^45^, and single-copy T0 events were identified and self-pollinated. Segregating null and homozygous T1 progeny were collected, and leaf discs were excised and vacuum-infiltrated with either Es solution or water control. Treated disks were incubated, and DsRed-Express RNA and protein levels were quantified over a 3-day period. Results clearly showed Es-dependent DsRed-Express protein expression by 24 h, which steadily increased up to 72 h (Fig. 3b). DsRed-Express RNA was detected within 1 h and started to plateau between 24 and 72 h post-treatment (Fig. 3c). Both DsRed-Express protein and RNA were below the level of detection in untreated transgenic and treated non-transgenic controls.

Interestingly, although robust reporter activation was observed in maize in vitro, we were unable to induce expression in V6 stage greenhouse-grown plants using a conventional herbicide spray test or soil drench. We could only induce expression in mature plant leaves by applying Es dissolved in DMSO, but expression was limited to the application area.

### Use of SURs to facilitate the transformation of monocots

The morphogenic factors Wuschel2 (Wus2) and Baby boom (Bbm) have been demonstrated to stimulate growth of embryogenic calli and greatly increase transformation frequencies in monocots, including maize, sorghum, rice, and sugarcane^46,47^. However, their prolonged overexpression produces severe pleiotropic plant phenotypes and infertility. This problem was previously overcome using a desiccation-responsive^47^ or heat-shock inducible^48^ promoter to drive Cre recombinase-mediated excision of the *Wus2* and *Bbm* genes before regeneration. An alternative approach would be plants with pre-integrated inducible *Wus2*/*Bbm* that could then be used for multiple rounds of transformation (retransformation) with new trait genes. New traits can be later segregated away from the *Wus2*/*Bbm* locus.

We first tested the feasibility and efficiency of inducible embryogenesis mediated by SUR-controlled expression of Wus2 and Bbm in maize. For that, we created an Es-inducible *Wus2*/*Bbm* expression cassette within the T-DNA (plasmid PHP63015) by positioning a single *TetO* sequence near the *Nos* promoter driving *Wus2,* and 3 *TetO* sequences near the TATA box and transcription start site of the *ZmUbi* promoter driving *Bbm*. PHP63015 also contained the repressor gene under a strong, constitutive maize ubiquitin promoter (*ZmUbi*_*pro*_), the *HRA* marker driven by the *Sorghum bicolor* ALS promoter providing resistance to Es, and a *CaMV* *35S Enhancer*-*HvLTP2*_*pro*_^49^:*ZsYellow*^50^ fluorescent protein expression cassette (Fig. 3d, Supplementary Table 3). Thus, we could use Es to simultaneously select for transformants and to induce morphogen expression/plant regeneration, and yellow fluorescence to additionally confirm successful transformation.

Stable PHP63015 transformants were grown for about 3 weeks, just before the emergence of the tassel. The flag leaf—protected from the environment and thus uncontaminated—was isolated, cut into fragments, and placed on callus induction media with or without 250 ng/mL (~0.61 μM) Es. The fragments were grown for 4 weeks and transferred for 2 more weeks onto regeneration media. Untreated leaf fragments failed to demonstrate any growth (0/14), whereas 8/14 Es-treated leaf fragments regenerated full seedlings (Fisher’s exact test, *p* = 0.0019, Fig. 3e, f).

We then tested the retransformation capacity of SUR-controlled Wus2/Bbm-expressing plants in the rice model (Fig. 3g). Immature rice (*Oryza sativa* cv. Kitaake) embryos were first transformed with *Agrobacterium* strain LBA4404 THY- carrying vector PHP60850. PHP60850 contained the same configuration of the *Wus2/Bbm/EsR/HRA* expression cassettes as PHP63015 used in maize experiments, but with a *ZmGz*_*pro*_:*amCyan* expression cassette placed upstream of the *Wus2* expression cassette in PHP60850 instead of the one expressing ZsYellow placed downstream of the *HRA* expression cassette in PHP63015 (Fig. 3d, Supplementary Table 3). This transformation produced 7 independent T0 events (either single- or 2-copy for the T-DNA). After regeneration of plantlets, leaf tissue from a single plantlet (complete with tillers) from each event was cut into segments and infected with *A. tumefaciens* strain AGL1 harboring a T-DNA vector PHP32269^51^ with two expression cassettes: *ZmUbi*_*pro*_*:PMI* and *ZmUbi*_*pro*_:*moPAT-ZsYellow* (the maize-optimized phosphinothricin acetyltransferase (moPAT) structural gene^52^ fused to ZsYellow) (Fig. 3h, Supplementary Table 3). After retransformation, leaf tissue was incubated on media with Es and mannose for 6 weeks. In this case, Es acted as an inducer of embryogenic callus formation and a selector for the *Wus2*/*Bbm* locus, while mannose ensured selection for the introduced vector PHP32269. Calli were further transferred to maturation media, then rooting media without Es, and the resulting plantlets were transferred to soil (Fig. 3g, i–l). The number of transformed callus events arising from each of the 7 starting T0’s ranged from 4 to 41, while the frequency of regenerating apparently normal, fully fertile plants from calli arising from each T0 was 56-95%.

### Increasing ligand sensitivity *in planta* via SUR autoregulation

**Fig. 4:**
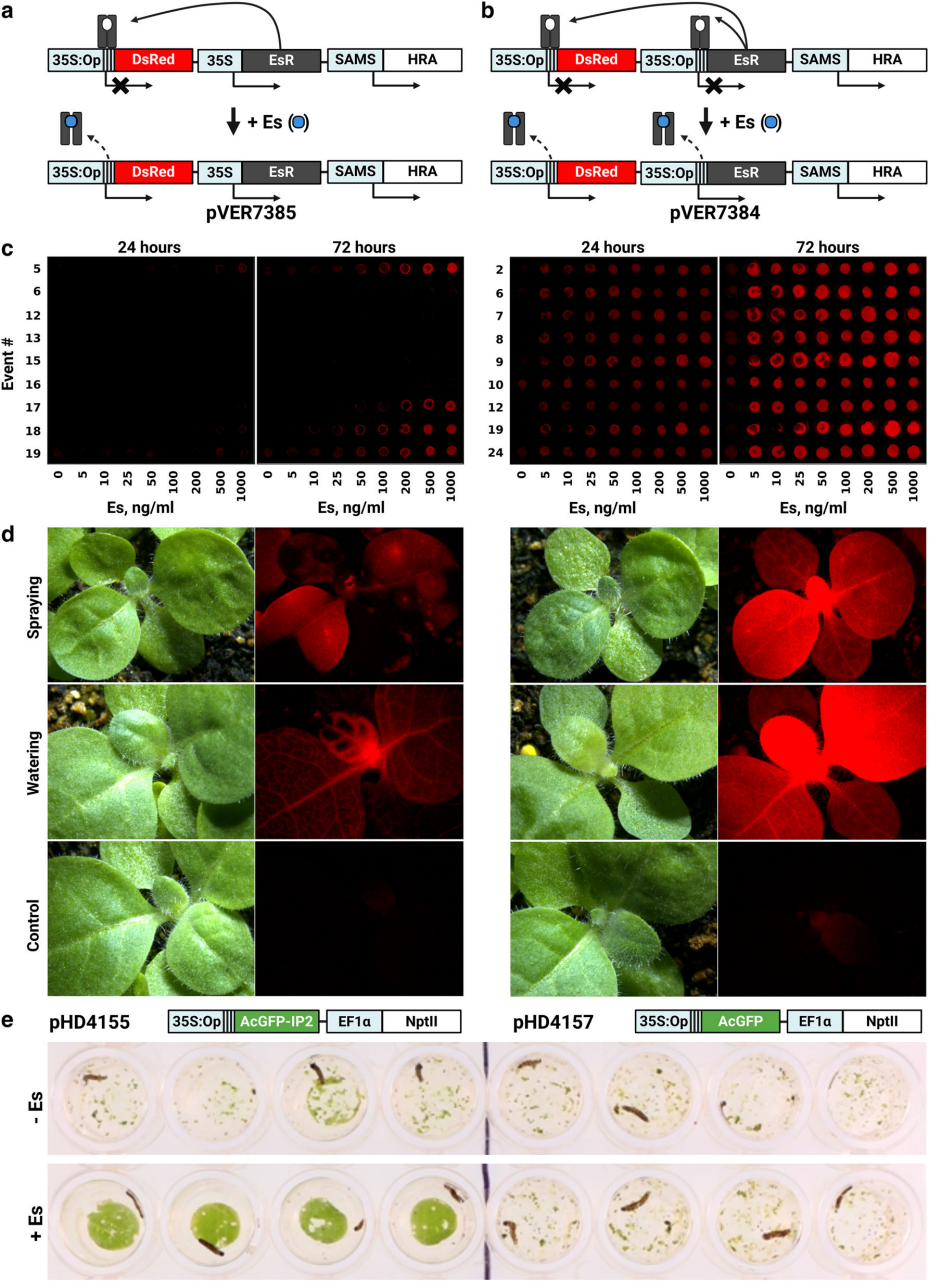
Increasing ligand sensitivity *in planta* via repressor autoregulation. Design of vectors pVER7385 (**a**) and pVER7384 (**b**). Created in BioRender. Looger, L. (2026) https://BioRender.com/38dpfdy. Light blue: promoters. Red: DsRed-Express reporter gene. Gray: repressor. White: *HRA* selection marker. Dark blue circle: Es ligand. **a** Top: without Es, EsR occupies the single *35S-Op* promoter, blocking fluorescent protein expression. Bottom: upon binding Es, EsR dissociates from the *35S-Op* promoter, allowing DsRed-Express expression. **b** Top: without Es, EsR occupies both *35S-Op* promoters, blocking fluorescent protein expression and inhibiting its own expression. Bottom: upon binding Es, EsR dissociates from the *35S-Op* promoters, allowing both DsRed-Express and EsR expression. **c** Induction of DsRed-Express expression in leaf disks from transgenic tobacco lines with constitutively (pVER7385; left) or autoregulated (pVER7384; right) repressor expression after 24 and 72 h on Murashige & Skoog agar with Es at the indicated concentrations. Photographs were taken with identical exposure times and camera settings. Autoregulated lines show stronger derepression but higher leakiness. **d** Es-dependent expression of DsRed-Express in tobacco seedlings harboring constitutive (pVER7385; left) or autoregulated (pVER7384; right) repressor after spraying or watering with Muster® or water (control). Shown is the expression on the 3rd day after treatment. Left—bright field, right—DsRed filter set. All photographs were taken with identical exposure times and camera settings. **e** Es-dependent induction of Bt toxin IP2-127 insecticidal activity in tobacco leaf disks. Tobacco leaf disks carrying a single copy of pHD4155 containing AcGFP with a C-terminal fusion of insecticidal protein (left) or pHD4157 encoding AcGFP negative control (right) events (4 each) in a pHD1180 tobacco line expressing autoregulated EsR without (top) or after treatment with 100 ng/mL (~0.24 μM) Es (bottom), 48 h after introduction of 2nd instar *Helicoverpa zea* larvae. An intact leaf fragment indicates expression of the insecticidal protein IP2-127. Partially created in BioRender. Looger, L. (2026) https://BioRender.com/38dpfdy.

After demonstrating the engineered repressors’ general usability in multiple plant systems, we focused on making improvements to the expression system to increase responsivity to ligand. For this, we decided to use a negative autoregulation approach whereby the repressor regulates its own expression level through operator sequences incorporated into the promoter driving its transcription. This ensures an optimal repressor concentration level that requires less inducer to saturate the pool of repressor molecules, cause operator derepression, and induce expression of the reporter gene. This method has been shown to improve response amplitude and kinetics in theory^53^ and experimentally in *E. coli*^54^ and yeast^55^, the latter application employing TetR. To test this approach in plants, we created two vectors with the *35S-Op*:*DsRed-Express* fluorescent reporter, constitutively expressing HRA, and either a constitutive (pVER7385, Fig. 4a, Supplementary Table 3) or autoregulated (pVER7384, Fig. 4b, Supplementary Table 3) EsR (L13-23) repressor.

Each vector was introduced into *A. tumefaciens* strain EHA105 and used to transform tobacco (*N. tabacum* cv. Xanthi NN) leaves. Transformed leaf pieces were selected with imazapyr—a non-SU herbicide that inhibits ALS (but not HRA) but does not interact with our designed repressors (and thus will not affect expression of genes driven by *Tet*-regulated promoters). Transformed lines that showed imazapyr resistance but no background DsRed-Express expression (thus active repression) were developed into plants. Leaf discs from plants with a single copy of the vector were then tested for DsRed-Express induction after exposure to one of eight Es concentrations for 24 or 72 h (Fig. 4c). Disks from plants with autoregulated repressor gene expression (pVER7384) were robustly derepressed by as low as 5 ng/mL (~12 nM) Es, whereas disks from plants with constitutively expressing repressor (pVER7385) required much higher Es levels.

We then tested the effect of SUR autoregulation in intact plants. Stable transformants from both constructs were treated with Muster® at the registered application level (200 mg Es/L (~490 μM)) through leaf spraying or base watering. Control plants were left untreated. Plants with the autoregulated repressor (pVER7384) demonstrated dramatically increased reporter gene activation in both treatment modalities (Fig. 4d). Untreated pVER7385 plants were dark; control pVER7384 plants showed very dim, but non-zero, fluorescence (Fig. 4d, bottom row). Thus, the negative autoregulation strategy indeed increased the system’s sensitivity to the ligand but at a cost of slightly increased leakiness.

### Deployment of the SURs in *Arabidopsis*

*Arabidopsis* is by far the most popular basic research plant model. While TetR-controlled gene expression has been successfully used in *Arabidopsis* before^56^, and the use of antibiotics is less of a concern in the laboratory setup, the plant biology field still might benefit from an alternative inducer, given the photosensitivity of Tc and its derivatives.

To test the SURs in *Arabidopsis*, we used the same pVER7384 vector (Fig. 4b, Supplementary Table 3) containing an autoregulated repressor. Homozygous T2 seedlings of inducible *A. thaliana* transformants with a single-copy insertion were sprayed at the rosette stage with Ally® (Ms), Classic® (Ci), Glean® (Cs), or Muster® (Es) at 0.25× application level (50 mg/L). A null segregant treated with Classic® was used as a control. Interestingly, Ally®, Classic®, and Glean®, but not Muster® (which contains the best ligand for the used EsR L13-23), induced widespread DsRed-Express expression within 3 days of application (Supplementary Fig. 18), similar to Muster®-sprayed tobacco (Fig. 4d, top row, right column).

### Controlling field traits using autoregulated SURs

Insects are a serious threat to crop plants. Fortunately, the superfamily of *Bacillus thuringiensis* (Bt) toxins has been widely developed for use in diverse crops to protect against insects^57^. While constitutive expression of Bt toxin has been useful for crop protection, its inducible expression could reduce metabolic demands on the plant and reduce concerns about off-target effects on beneficial species.

**Fig. 5:**
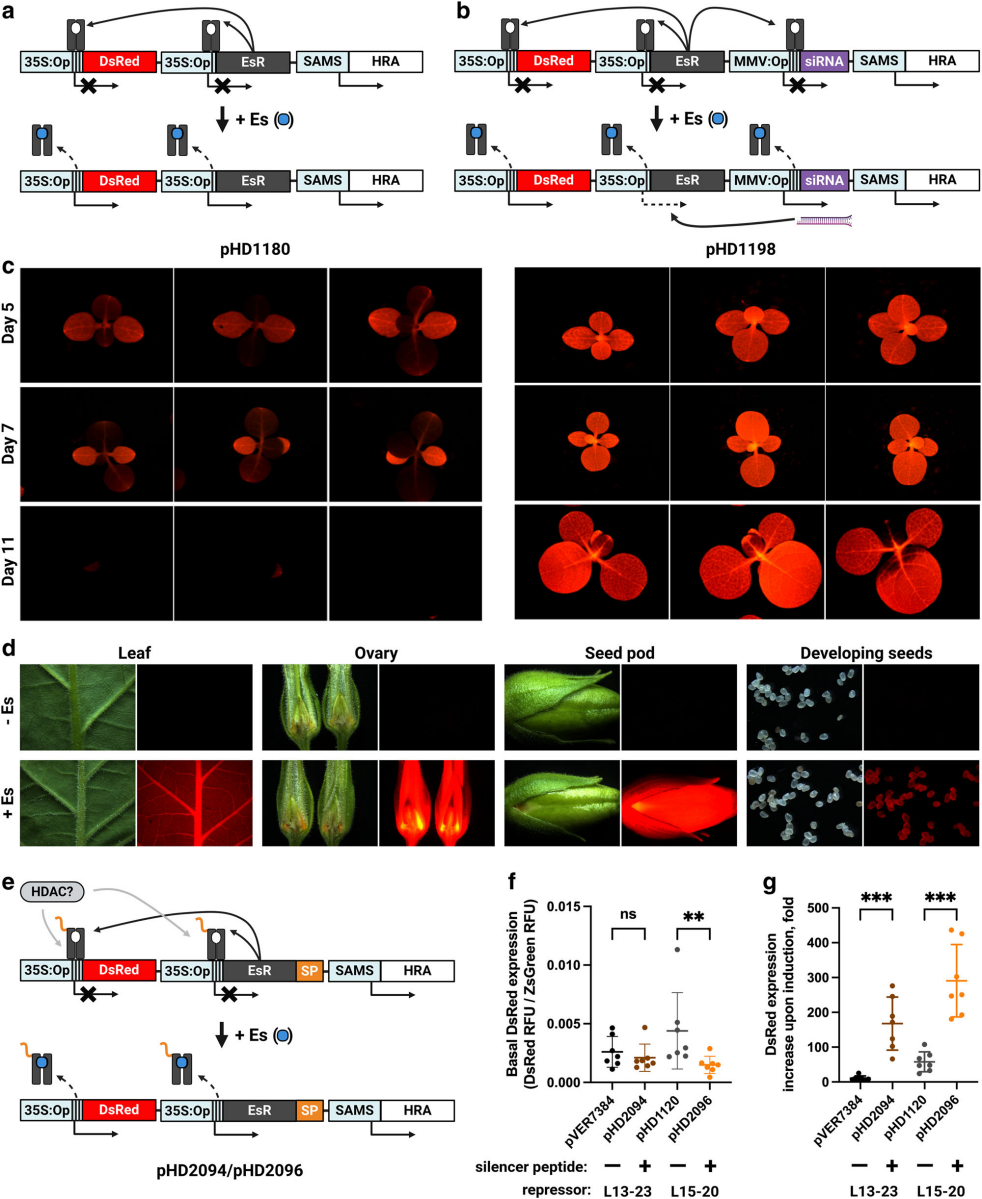
Modifications to the SUR expression system. Design of vectors pHD1180 (**a**) and pHD1198 (**b**). Created in BioRender. Looger, L. (2026) https://BioRender.com/vbv5r80. Light blue: promoters. Red: DsRed-Express reporter gene. Gray: repressor. White: *HRA* selection marker. Dark blue circle: Es. **a** Top: without Es, EsR occupies the *35S-Op* promoters, blocking DsRed-Express expression as well as expression of itself. Bottom: upon binding Es, EsR dissociates from the *35S-Op* promoters, allowing both DsRed-Express and repressor expression. **b** Purple: siRNA targeting repressor. Top: without Es, EsR occupies the *35S-Op* and *MMV-Op* promoters, blocking DsRed-Express, siRNA_REP_, and repressor expression. Bottom: upon binding Es, residual EsR dissociates from the *35S-Op* and *MMV-Op* promoters, allowing DsRed-Express, EsR, and siRNA expression. siRNA, in turn, eliminates old and new EsR transcripts. **c** Es-induced derepression of DsRed-Express expression in tobacco seedlings from three pHD1180 (no siRNA) and three pHD1198 (with siRNA) events on days 5, 7, and 11 post-transplantation. Each column represents the same plant. Shown are three representative plants out of 12 tested for each group. Imaging settings for fluorescence micrographs were identical. **d** DsRed-Express expression in various plant tissues of pHD1198-2 plants at different stages of development following water (top) or Muster® (bottom) treatment. Imaging settings for micrographs were identical within each tissue type. Left—bright field, right—DsRed filter set. **e** Design of vectors pHD2094 and pHD2096. Light blue: promoters. Red: DsRed-Express reporter gene. Gray: repressor. Orange: silencer peptide. White: *HRA* selection marker. Dark blue circle: Es. Top: without Es, EsR occupies the *35S-Op* promoters, blocking DsRed-Express expression and inhibiting its own expression. The silencer peptide of the repressor also recruits additional silencing agents (HDAC, light gray), which induce further locus silencing. Bottom: upon binding Es, EsR dissociates from the *35S-Op* promoters, allowing expression of DsRed-Express and additional EsR. Created in BioRender. Looger, L. (2026) https://BioRender.com/vbv5r80. **f**, **g** Effect of added silencer peptide on EsRs L13-23 and L15-20. Background signal (**f**) and derepression magnitude (**g**) were assessed in tobacco leaves. The plots show *n* = 7 experimental replicates. Shown are the mean and std. dev.; ns not significant, **P  <  0.01, ***P < 0.001, two-tailed Mann–Whitney test; *P* values are 0.535, 0.004, 0.0006, and 0.0006, respectively. Source data are provided as a Source data file.

Having established that we could robustly and on-demand induce expression of reporter genes, we next sought to establish inducible expression of the Cry2A Bt toxin IP2-127^58^, which is highly active against lepidopteran insects such as corn earworm (*Helicoverpa zea*). We created two expression vectors, pHD4155 and pHD4157, driving the AcGFP-Bt fusion or just a control AcGFP^59^, respectively, from a *35S-Op* promoter. Both vectors also contained an *Ef1a:nptII* cassette to facilitate selection on kanamycin (Fig. 4e, top, Supplementary Table 3). Tobacco plants stably transformed with vector pHD1180 (autoregulated EsR controlling DsRed-Express production, Fig. 5a, Supplementary Table 3) were retransformed with *Agrobacterium* carrying pHD4155 or pHD4157. Single-copy transformants with inducible AcGFP expression were then identified for both lines. Selected single-copy events (three events for each line, four technical replicates for each event) were tested for insect resistance by introducing 2nd instar *H. zea* larvae to leaf disks on agar with or without 100 ng/mL (~0.24 μM) Es for 48 h. In the absence of the inducer, all leaf disks were consumed, whereas in the presence of Es, pHD4155 leaf disks survived predation, while pHD4157 controls were consumed (Fig. 4e). Thus, important field traits can be readily controlled in crop plants with the SURs.

### Increasing temporal and spatial ligand-induced derepression using siRNA

An inducer applied a single time will eventually break down, resulting in re-repression of the target gene expression. While this can be beneficial for many applications, in some cases, longer activation might be desired. Moreover, induction propagation in large, complex tissues, in which penetration and distribution of small molecule inducers can be uneven (sometimes dramatically so), also presents a challenge. To achieve a more prolonged and penetrating induction, we added a short inhibitory RNA (siRNA)^60^ targeting the repressor (siRNA_REP_) under a repressor-regulated *MMV-Op* promoter, creating vector pHD1198 (Fig. 5b, Supplementary Table 3). The vector pHD1198 was made from pHD1180, essentially identical to pVER7384, but with only a single *TetO* sequence in the inducible *35S-Op* promoter driving EsR (opposed to three in pVER7384; Figs. 4b and 5a, Supplementary Table 3). We chose pHD1180 over pVER7384 as the parental vector for the siRNA experiments, as it should provide a slightly higher basal level of SUR expression and, hence, reduce the background reporter gene (as well as siRNA_REP_) expression that was observed in the case of pVER7384. In the absence of an inducer, no siRNA is produced, repressor expression is low, and the reporter locus is silenced. In the presence of an inducer, however, anti-repressor siRNA is produced, decreasing levels of repressor, and ensuring that even small amounts of inducer are sufficient to bind to the remaining repressor molecules, leading to full derepression. Importantly, siRNA signals can become systemic in plants, spreading to all tissues^61–63^. Vector pHD1180 was used as the control vector for pHD1198 experiments.

The vectors were transferred to *A. tumefaciens* strain EHA105 and transformed into leaf explants of wild-type tobacco (*N. tabacum* cv. Xanthi NN), followed by selection on 50 ng/mL imazapyr. Duplicate excised leaf disks from each transformant were screened for DsRed-Express gene expression in the absence and presence of Es, and then quantified for integration copy number by qPCR. Seeds of single-copy inducible T0 events were germinated on filter paper soaked with 50 ng/mL (~0.12 μM) Es, transplanted to soil without inducer, and imaged at 5, 7, and 11 days. DsRed-Express expression was modest and diminished over time (effectively gone after 11 days) in pHD1180 events, but was high and remained so over time in lines with the *MMV-Op:siRNA*_*REP*_ cassette (Fig. 5c), confirming the expected siRNA-driven spatial and temporal amplification of derepression.

In a follow-up experiment, T1 seeds of siRNA_REP_-containing line pHD1198-2 were planted in soil, and seedlings at the 4-leaf stage were treated with water or a one-time application of 20 mL 0.125× Muster® solution (500 μg Es). After 11 days, the pots were flushed extensively with clean water, and the plants were transferred to 1-gallon pots and allowed to grow to maturity. The DsRed-Express phenotype was fully activated in the induced plants throughout their lifespan in all tissues, including root, leaf, stamen, anther, ovary, and developing and mature seeds (Fig. 5d, bottom), but was silent in plants treated with just water (Fig. 5d, top). Thus, the addition of the anti-repressor siRNA dramatically increased the magnitude and extent of the derepression in intact plants, across tissue types and time.

### Enhancing autorepression with silencer peptides

While the reduction of the number of *TetO* sequences to one in the SUR promoter of the autoregulated repressor system seemed to reduce basal DsRed-Express expression compared to vector pVER7384 with three *TetO* sequences (Fig. 5d, top; Fig. 4d, bottom row), it should also reduce the system’s sensitivity to the ligand. As an alternative way to reduce basal reporter gene expression, we tested the fusion of a small transcriptional silencer peptide element from *Arabidopsis* transcription factor AtMYBL2, responsible for transcriptional silencing of anthocyanin biosynthesis^64^, to the C-terminus of the repressor. Thus, in addition to blocking transcription by physically occupying the *TetO* elements surrounding inducible promoters, these bifunctional repressors would also recruit additional silencing agents (most likely histone deacetylase (HDAC), analogous to Ethylene-responsive element binding factor-associated Amphiphilic Repression (EAR) motifs^65^).

EsR L13-23 or EsR L15-20, in plasmids pVER7384 or pHD1120, respectively, were extended with a 6-amino acid transcriptional silencer peptide (TLLLFR). The resulting vectors, pHD2094 and pHD2096 (Fig. 5e, Supplementary Table 3), respectively, were tested for basal expression and derepression alongside control vectors pVER7384 and pHD1120 using a rapid *Agrobacterium*-based *N. benthamiana* leaf infiltration assay. Interestingly, only the construct encoding EsR L15-20 with the silencer peptide motif showed decreased baseline expression (Fig. 5f), but both showed 6-10-fold increased derepression (Fig. 5g). The latter might be explained by more stringent autorepression, which reduces the total concentration of repressor that needs to be titrated by the added ligand.

## Discussion

Inducible expression systems are workhorses in biology. Several naturally evolved systems have been deployed in almost every organism amenable to heterologous expression^16,66^. However, no available technology is suitable for deployment in crop plants. As such, current genetically modified crops express transgenes constitutively, with inducibility limited to the use of tissue-specific promoters. This restricts traits that would be useful on an as-needed basis during the growing season, or that negatively impact seed production or viability.

We sought to address this need by reengineering wtTetR^67^, which evolved naturally to recognize antibiotics, to respond to more practical inducers. While the TetR-Tc pair works in plants, Tc and related antibiotic TetR inducers are incompatible with field use because they are photosensitive and toxic to plants and soil biota^68^, and, hence, not registered for field use. We established an *E. coli*-based screening system for evolving mutant TetR-ligand pairs, and used it to engineer robust repressors inducible by commercially registered SU herbicides^24^, which are characterized by rapid uptake and movement throughout plant tissues. We developed high-quality repressors responding to the herbicides Es (Muster®) and Cs (Glean®). SUR-SU pairs developed in *E. coli* were validated by biophysical characterization, high-resolution X-ray crystallography, cell culture, and ultimately in plants.

Structure-based computational design was used to significantly reduce the sequence diversity tested in the initial round of library design. However, substantial rearrangement of the flexible loops around the ligand-binding pocket (causing the repositioning of key interacting residues) and ligand flexibility would have made it difficult to predict successful mutation combinations purely computationally. This emphasizes the utility of combining in silico modeling with directed evolution. This is especially critical in systems like TetR, where protein function depends on dramatic ligand-induced conformational changes, and where a second, conditional interaction (with DNA in the case of TetR) also plays a crucial role.

The baseline repressor efficiency and ligand-driven induction of the best SUR-SU pairs approach that of wtTetR-Tc. Structural analysis confirmed that the robust activity of the SUR-SU switches results from complex networks of hydrogen bonds and hydrophobic interactions between the proteins and SUs. However, it remains unclear whether the SUR-SU derepression mechanism operates at the atomic level in the same way as TetR-Tc, particularly in the case of CsR-Cs. This again emphasizes that SUR design would likely not be achievable with computational modeling alone.

Some optimization steps were required to convert designed EsRs into a robust inducible expression system with low background, high induction, and broad spread throughout plant tissue. Critically, the expression of repressors was placed under its own control (negative autoregulation), which improved sensitivity to the ligand. Indeed, this is how the TetR-mediated response to Tc is regulated in nature^69^. Next, we incorporated a second feedback loop, where the repressors regulate not only their own expression but also that of repressor-targeting siRNA molecules. This enhancement resulted not only in rapid and strong induction but also in the spread of the induction throughout the plant and the persistence of induction over time, through seed formation. This technology has the potential to enable single-season traits and eliminate environmental persistence, addressing many safety concerns of transgenic crops. Finally, we further reduced reporter gene background expression and increased induction strength by adding a small transcriptional silencer peptide element to the C-terminus of the repressor. Thus, the repressors not only block transcription by occupying binding elements in inducible promoters, but also by recruiting additional silencing agents (most likely HDAC).

We showed that the EsR-Es switch functioned well in tobacco and soybean plants, as well as in vitro in cultured maize and rice. However, in *Arabidopsis*, the switch responded poorly to Es, while showing strong induction by other SUs such as Cs, Ms, and Ci. The failure to induce expression in maize via Es spraying and the weak induction of expression in *Arabidopsis* by Muster® (a SU herbicide registered for oilseed rape, a close relative of *Arabidopsis*) may stem from these plants’ rapid metabolization of Es, which likely limits systemic compound spread. A similar behavior has been previously reported for the rice SU herbicide pyrazosulfuron-ethyl^70^. Hence, ligands that were sub-optimal in vitro but are not metabolized may function more effectively in vivo than expected. Thus, the existing EsR switch may work well in many crops, with Es as the preferred ligand in species that do not rapidly metabolize it, and with other SU herbicides in plants that do. To enable robust field deployment, optimal combinations of crop, compound, and repressor will need to be identified for each specific case.

In its current form, EsR usage requires co-expression of HRA to fully tolerate inducer application. Such alleles have been used as selectable markers in a range of plant species (e.g., *Arabidopsis*^71^, tobacco^72,73^, rice^74^, canola^75^, maize^76^, potato^77^, soybean^25,78^). Luckily, many ALS-targeting herbicides do not interact with EsR. Hence, the *HRA* gene can be used as a selectable marker for transgenic variants without causing derepression. For example, we were able to select transformants using imazapyr, an imidazolinone ALS inhibitor. This enables recovery of transformants having repressed transgenes, whose expression would be detrimental to growth or development.

Thus far, we have observed no deleterious effects on plant growth or development from the resting repressor gene. However, we occasionally saw stunting of growth in some clones after herbicide treatment. Since the herbicide-resistance gene *HRA* was usually placed under the control of a relatively weak promoter in the vectors used, it is likely that some clones did not produce enough HRA to resist the herbicide dose used. Also, during transformation, gene constructs are inserted into random locations of the chromosome, which can result in higher or lower expression of the transgenes. Low expression resulting from insertion position effects may not be detected during event selection but may show up later in whole-plant herbicide treatment experiments. Placing *HRA* under a stronger promoter may address this problem. This is especially important with root soak experiments, where the herbicide is present at higher doses for extended periods of time. Alternatively, it might be possible to eliminate the need for the *HRA* marker entirely by evolving repressor specificity towards a non-herbicidal (yet registered) SU variant or another agrochemical. It should be possible to achieve this using the pipeline we developed here.

The repressor system developed here can be useful for many applications. This technology can facilitate conditional regulation of beneficial trait genes (as we demonstrated with Cry2A Bt toxin expression), while simultaneously controlling weeds to optimize crop performance. We also showed an increase in transformation efficiency in monocots with inducible Wuschel2 (Wus2) and Baby boom (Bbm) expression with no deleterious effects on plant growth. With latent gene activation, it may also be possible to unlock high-value traits incompatible with commercial seed production or to maintain dominant male-sterile parents for hybrid seed production. Alongside other inducible expression systems, SURs can be useful for facilitating work with difficult-to-transform species (like monocots), increasing the efficiency of producing marker-free transgenic plants^79^, annotating the function of genes whose overexpression or silencing prevents recovery of transgenic plants or production of viable seeds^80–82^, and other basic plant biology studies.

It is possible that the benefits of the developed induction system could lead to the field application of SU herbicides beyond their current applications in weed control. While this is not ideal from an environmental perspective, for induction purposes, SU compounds could be applied at even lower concentrations than those currently used in standard agricultural practice, and they are among the lower-toxicity herbicides. Moreover, this work establishes the feasibility of substantial shifts in substrate specificity, which we expect will ultimately enable the development of switches responsive to even safer, more selective, and/or non-herbicidal inducers in future iterations.

Additionally, the engineered repressors could find application in many fields of biotechnology and basic research beyond plants. The toxicity of the SU herbicides to animals is listed as quite low^83^. Indeed, similar molecules are used as drugs in humans to treat type II diabetes^84^, and we did not notice any toxicity in our cell culture experiments. Engineered SURs should be easily convertible into Tet-Off^85^ system analogs through fusion with a transcriptional activation domain. It may also be possible to reverse the repressors engineered here such that they bind the *TetO* operator only in the presence of ligand and dissociate from it in its absence. Such reverse repressor variants have been previously made from wtTetR and form the basis of the Tet-On systems^86–90^. The Tet-On/Tet-Off systems are widely applied in many model organisms^16,91^. Given the robustness of SURs in mammalian cells, the SU-On/SU-Off system might be a great complement to Tet-On/Tet-Off or even a replacement for experiments where doxycycline’s toxicity^92^ or photosensitivity becomes significant.

## Methods

### SU docking and initial computational design

PDB structure 1DU7 was used for the redesign of the TetR binding pocket. Although the entry is annotated as containing 4-epi-tetracycline, the ligand in the structure is 7-chlortetracycline. This structure has since been obsoleted and superseded by PDB entry 2X9D^93^, which contains iso-7-chlortetracycline, a degradation product of 7-chlortetracycline. Despite its obsolete status, the 1DU7 coordinates remain accessible from the PDB. Alternative 7-chlortetracycline/TetR structures (e.g., 2TCT^94^) may serve as suitable starting points. However, slight differences in protein and ligand conformations may lead to non-equivalent modeling outcomes.

Residues interacting with 7-chlortetracycline, as well as residues that could interact with a somewhat translated and/or rotated Ts molecules, were identified by inspection, yielding 17 positions (60, 64, 82, 86, 100, 104, 105, 113, 116, 134, 135, 138, and 139 from chain A; 147, 151, 174, and 177 from chain B; Supplementary Fig. 1b). To create a protein scaffold for ligand docking, 7-chlortetracycline was removed, and these side-chains were trimmed in silico to alanine.

A starting atomic model of Ts was created with Chem3D (PerkinElmer). To model realistic internal SU conformations, we gathered all available structures of sulfonylurea herbicides in the Protein Data Bank: bensulfuron-methyl (5FEM, 6DEM); Ci (1N0H, 1YBH, 6DEL); Cs (1T9B, 1YHZ); iodomuron-ethyl (6DEN); monosulfuron (3E9Y); monosulfuron ester (3EA4); Ms (1T9D, 1YHY); Sm (1T9C, 1YI0, 6DEP); and Tb (1T9A, 1YI1) and superimposed all molecules at the SU moiety. This revealed that: (1) the triazine ring is coplanar with the SU, consistent with its pi conjugation, (2) alkoxy groups on triazine rings extend this coplanar system; (3) by contrast, benzoate esters lie roughly perpendicular to the phenyl ring; (4) the phenyl ring can extend at 60° or 120° from the sulfonylurea moiety; and (5) the phenyl ring can rotate around the C–S bond. pK_a_ data suggested that the amide proton closest to the sulfonyl group (i.e., the SU proton) would be protonated at physiological pH, whereas the adjacent proton would be deprotonated. We modeled Ts accordingly. Given the observed dihedral angles of crystallized SUs, we created a library of Ts conformers for modeling: (1) the methoxy group on the triazine ring was coplanar with the ring, either cis or trans; (2) the methyl ester on the thiophene ring was perpendicular to the ring, either 90° or 270°; (3) the thiophene ring extended 60°, 120°, 240°, or 300° relative to the N-S bond; (4) the thiophene ring extended 120°, 240°, or 300° relative to the sulfonyl; and (5) the thiophene ring rotated 0°, 30°, 60°, 90°, 120°, 150°, 180°, 210°, 240°, 270°, 300°, or 330° relative to the S-C bond. This produced a total of 144 Ts conformers.

Each of the 144 Ts internal conformers was both rotated and translated within the trimmed TetR scaffold to find ligand poses (consisting of a conformation, a rotation, and a translation) consistent with the protein backbone and possible side-chain placements. Rotations were modeled using quaternions^95^ using 10 intervals for *s*, 15 intervals for *q*_1_, and 30 intervals for *q*_2_; of these 4500 possible rotations, 1320 were <0.01 distance of (*w,x,y,z*) on the unit sphere of another rotation and deemed redundant, leaving 3180 rotations. Within the trimmed binding pocket, a cubic lattice of points was created, centered at the center of mass of 7-chlortetracycline, with grid spacing of 0.25 Å, and a range of ±2.5 Å from the local origin. This produced a total of 4913 possible translations of the Ts. Thus, there were a total of 144 × 3180 × 4913 = 2.3 × 10^9^ total possible Ts placements in the binding pocket.

First, sequence redesign of the ligand-free protein scaffold was performed (allowing all 17 positions to mutate to any amino acid except cysteine), optimizing a molecular mechanics potential with hydrophobic, electrostatic, hydrogen-bonding, and solvation terms using our protein design software Chameleon. Analysis of the resulting designs ensemble yielded a library of scaffold-compatible amino acid identities and their side-chain conformations for each of 17 positions.

For each Ts placement, the ligand-scaffold interaction energy was first calculated using the same molecular mechanical potential, and the placement was discarded if the calculated energy was above 0.0 kcal/mol. For the remaining placements, for each of 17 ligand-adjacent amino acid positions, the side-chain conformation most favorably interacting with the ligand was selected from the previously precalculated scaffold-compatible library, with no regard for side-chain–side-chain interactions. The sum of these interaction energies represents a best-case scenario for possible ligand-protein interaction. Ligand placements with this best-case interaction energy above −90 kcal/mol (chosen to produce a rich yet reasonably sized set of placements) were eliminated. This left 1811 ligand placements deemed possibly compatible with the protein scaffold.

These ligand poses were quite diverse. We used hierarchical clustering to sort them into 20 clusters (Supplementary Fig. 2a). For each cluster, the median of all ligand poses was computed, and the ligand closest to that median position was selected to represent the cluster (Supplementary Fig. 2b). For each cluster, this representative ligand placement was used for full binding pocket sequence redesign (allowing all side-chains but cysteine) using a derivative of the dead-end elimination^96,97^ and Fast and Accurate Side-chain Topology and Energy Refinement algorithms^98^ that output 1000 low-energy designs. For each ligand placement, at each of the 17 mutated positions, a set of favorable amino acids was determined based on their average and best energy in the 1000-member sequence ensemble. Then, for each of the 17 mutated positions, 20 sets of amino acids (one for each ligand placement) were merged, taking amino acids that appeared as top choices for multiple ligand placements. This produced a final computational design amino-acid library with 4, 5, 4, 4, 5, 3, 8, 11, 10, 10, 8, 8, 7, 9, 6, 7, and 5 amino acids, respectively (Supplementary Fig. 3, column 1), deemed likely to favorably contribute to interactions with Ts, yielding a theoretical library size of ~4 × 10^13^ for the initial computational design.

### Construction of *E. coli* host strain KM3

*E. coli* Top10 [genotype F- *mcrA* Δ(*mrr-hsdRMS*-*mcrBC*) φ80*lacZ*ΔM15 Δ*lacX74 recA1 araΔ*139 Δ(*ara-leu*)7697 *galU galK rpsL* (StrR) *endA1 nupG*] (Invitrogen) was subjected to two additional chromosomal modifications via PCR-based gene replacement^99^. First, the *tolC* locus was replaced with a *Plac*::*nptIII* fusion (neomycin phosphotransferase III, a more active mutant of the more commonly used *nptII*). Deletion of *tolC*, a component of many efflux pumps, ensures maximal penetration of SU compounds^100^. Top10 cells carrying the desired replacement were selected by kanamycin (Kan) resistance. Second, the *malE* locus was replaced with a bidirectional *PtetR*::*lacI* (leftward direction of transcription)/*PtetA*::*lacZ*::*aadA* (rightward direction; *aadA* encodes spectinomycin (Sp) adenylyltransferase and thus Sp resistance) operon. The desired cells were selected by Sp resistance. The resulting strain *E. coli* Top10 [Δ*tolC* (*Plac*::*nptIII*), Δ*malE* (*PtetR*::*lacI*/*PtetA*::*lacZ*::*aadA*)], denoted KM3, is lacI^+^, lacZ^+^, Sp^r^, and Kan^s^. Transformation of KM3 with a plasmid expressing functional repressor results in blocking expression of *lacZ*, *aadA*, and *lacI* genes, relief of lacI repression of the *Plac*-controlled *nptIII* gene, and thus yields a lacI^−^/lacZ^−^/Sp^s^/Kan^r^ phenotype, which can be reverted back to lacI^+^/lacZ^+^/Sp^r^/Kan^s^ phenotype by addition of a ligand causing the dissociation of the repressor from the DNA (Supplementary Table 4).

### Vectors for bacterial screening

For initial library screening, a synthetic version of the Tn10 transposable element *tetR*(B) gene (GenBank X00694) was cloned downstream of the arabinose-inducible promoter (*P*_*BAD*_) of a medium-copy number, ampicillin-resistant, pBR322-derived vector utilizing NcoI/AscI restriction sites. The *tetR*(B) gene was designed with a SacI restriction site (CGAGCTCTG – Arg-Ala-Leu) bisecting the DNA-binding domain (DBD) and ligand-binding domain (LBD) to enable independent cloning of LBD (amino acids 50-207) libraries. This vector was denoted pVER7314. For later rounds of screening, vector pVER7334 was constructed by replacing the DBD of pVER7314 with its plant codon-optimized version to streamline *in planta* hit testing. Finally, a third vector, pVER7571, was made introducing a mutation to the Shine-Dalgarno ribosome-binding site (AggAGA → AcAGA) that we discovered decreases TetR expression level and thus facilitates the detection of leaky variants.

### DBD library construction

Oligonucleotides encoding the targeted amino acid variations (Supplementary Data 1, 3, 5, 7, 8, 10–15, 18 and 19) were assembled by overlap-extension PCR^101^ in six identical 25 μL reactions containing 0.5-1.0 μM pooled library oligos, 0.5 μM of each flanking primer, 200 μM dNTPs, and Herculase II (Stratagene) polymerase. Thermocycling was performed as follows: 98 °C for 1 min; 25 cycles of 95 °C for 20 s, 45–55 °C for 45 s, 72 °C for 30 s; and 72 °C for 5 min. The correct size band (~500 bp) was digested with SacI/AscI and ligated into the appropriate vector (pVER7314/pVER7334/pVER7571). The ligation mixture was then transformed into *E. coli* strain KM3 and plated onto LB agar supplemented with 50 μg/mL carbenicillin and 40 μg/mL kanamycin. Since we found TetR expression from the P_BAD_ promoter to be slightly leaky, no arabinose was used for the outgrowth or plating. In some cases (libraries L4, L10, L11, and L8), assembly was done using two sets of oligonucleotides: one encoding the parental construct (Supplementary Data 4, 9 and 16) and another encoding the desired diversity (Supplementary Data 3, 7, 8 and 15). First, equal amounts of oligonucleotides encoding diversity at the same annealing location were pooled to 10 μM mixes. Next, equal volumes of these oligonucleotide mixes were combined to obtain a 10 μM full library oligonucleotide stock. Likewise, oligonucleotides encoding the parental construct (and covering the same base positions as the diversity set) were combined to make a separate 10 μM stock. The assembly reaction was then carried out by mixing the diversity and the parental construct oligonucleotide stocks at different ratios.

### Site-saturation mutagenesis

Oligonucleotides containing NNK degenerate codons (Supplementary Data 6 and 17) were used to obtain clones with all possible amino acid substitutions at each of the targeted positions using QuickChange (Stratagene) mutagenesis. Desired variants substituted for all 20 possible amino acids at each target position were identified by DNA sequencing.

### Colorimetric colony screen

Following the transformation of *E. coli* KM3 with each library, cells were grown without selection for 2 h to allow for the repressor-conditional expression of the kanamycin resistance marker. After that, cells were plated onto LB agar supplemented with 50 μg/mL carbenicillin and 40 μg/mL kanamycin and incubated overnight at 37 °C to allow sufficient time for colony development. Colonies were picked using a Q-Pix robot (Genetix) into 384-well plates containing 60 μL of LB supplemented with 50 μg/mL carbenicillin, 40 μg/mL kanamycin, and 10% glycerol. The 384-well plates were incubated overnight at 37 °C, and the cultures were replica-plated onto M9 agar supplemented with 0.1% glycerol, 0.04% casamino acids (to prevent branched-chain amino acid starvation induced by SU herbicides^102^), 50 μg/mL carbenicillin, and 0.004% X-gal, with or without SU at the indicated concentration. For screening of round 1 and 2 libraries, the assay plates with SU were incubated at 30 °C for 48 h; for all later rounds, they were incubated at 37 °C overnight. Assay plates without SU were generally incubated at 37 °C overnight, followed by an additional 2–5 days at ambient temperature, to allow for more color development to better assess the leakiness of the repressor.

Colony color was either scored visually or imaged using a Nikon ACT-2U camera with a Canon EF 100 mm 1:2.8 macro lens and analyzed using a custom ImageJ^103^ script. Colonies exhibiting either improved induction (more intense blue colony color in the presence of ligand) or repression (less intense blue colony color in the absence of ligand, i.e., less leaky) relative to the parental clone were picked into 96-well plates containing 150 μL LB supplemented with 50 μg/mL carbenicillin and 10% glycerol. These hits were re-assayed to confirm improved activity and re-scored. In parallel, all hits as well as random clones were sequenced. Discovered trends in sequence correlation with repression and/or induction activities were used to select amino acid diversity for the next round of shuffling.

### β-galactosidase activity assays

For routine library screening, starter cultures were grown overnight in 96-well plates with 150 μL LB supplemented with 50 μg/mL carbenicillin, and 10–15 μL were sub-cultured into 150 μL of LB supplemented with 50 μg/mL carbenicillin with or without the ligand. After thorough mixing, the cultures were incubated at 37 °C in a shaking incubator at 190 rpm for approximately 2 h. After that, the cells were permeabilized by the addition of Polymyxin B Sulfate (PMBS) to a final concentration of 20 μg/mL. Five to twenty-five microliter aliquots of the treated cells were then added to 150 μL of 1 mM 4-methylumbelliferyl-β-D-galactopyranoside (MUG) in 50 mM sodium phosphate buffer, pH 7.0, with 1 mM MgCl_2_ and 0.1% β-mercaptoethanol and incubated at ambient temperature. Assay plates were read at 365 nm excitation/455 nm emission in a SpectraMax XS spectrofluorometer (Molecular Devices, SOFTmax PRO v4.6) using the kinetic format.

To compare hits from different libraries, the LBDs of all library hits were codon-optimized for *E. coli* expression, synthesized (Twist Bioscience), and cloned into pVER7314. The night before the experiment, eight small cultures (2 mL of LB supplemented with 100 μg/mL carbenicillin) were inoculated with a single colony from fresh plates: positive and negative controls, and six TetR variants for testing. As a positive control, we used KM3 *E. coli* constitutively expressing β-galactosidase transformed with a pVER7314 vector having the kanamycin resistance gene cloned in place of the repressor (pVER7314-Kan). Negative control consisted of Top10 *E. coli* (parental strain to KM3), also transformed with the pVER7314-Kan vector. The cultures were grown at 37 °C in a shaking incubator at 250 rpm for 15 h. The next morning, a 96-well microtiter plate with round-bottom wells was filled with 150 μL LB supplemented with 100 μg/mL carbenicillin and different concentrations of the evaluated ligand (0, 0.17, 0.5, 1.5, 4.6, 13.7, 41.2, 123.5, 370.4, 1111.1, 3333.3, and 10,000 ng/mL, obtained by serial threefold dilutions). All wells of each of the rows of the plate were inoculated with 10 μL from one of the overnight cultures. The cultures were then incubated at 37 °C in a shaking incubator at 250 rpm for another 2 h to induce the production of the β-galactosidase. Following incubation, samples were treated with 25 μL of 140 μg/mL PMBS for 5 min to permeabilize cells. 10 μL of the treated cultures were then transferred into a black flat-bottom plate containing 150 μL of the β-gal assay buffer (50 mM sodium phosphate, pH 7.0, 1 mM MgCl_2_, 0.1% β-mercaptoethanol, and 1 mM MUG). The fluorescence signal (Ex = 365 nm/Em = 455 nm) was read for 2 h on top of each minute (with 5 s shaking of the plate before each measurement) using a Tecan Spark microplate reader (SparkControl v3.1 SP1). The fragment of each resulting curve (signal <50,000 RFU) was fitted with a linear function, and the slope of the line was plotted as a function of ligand concentration. The EC_50_ was calculated (where possible) after fitting the latter to the 1:1 binding model using custom Python 3 scripts.

### Protein expression and purification

Proteins were cloned into a pET30-like vector containing a T7 promoter and an N-terminal 6xHis-tag followed by a thrombin protease cleavage site. The constructs were transformed into *E. coli* BL21(DE3), and 50 mL cultures (2xYT media with 50 μg/mL carbenicillin) were inoculated with a single colony. The cultures were then grown at 37 °C in a shaking incubator at 250 rpm until reaching an OD_600_ of 0.6, at which point IPTG was added to 1 mM and the temperature was lowered to 16 °C. After overnight expression, cells were harvested, resuspended in 3 mL of lysis buffer (30 mM imidazole, 50 mM sodium phosphate, 300 mM NaCl, 5 mM MgCl_2_, 1 mg/mL lysozyme, 50 μg/mL PMBS, 2 U/mL endonuclease, EDTA-free protease inhibitor cocktail), and incubated at 37 °C, 250 rpm for 1 h. Then 30 μL of Triton X-100 was added (to 1% v/v final concentration), and cells were incubated for an additional 30 min. Lysates were cleared by centrifugation at 4 °C for 30 min at 11,000 × *g*. 1 mL of Ni-NTA resin (Qiagen) was washed 3× with 10 mL of 50 mM sodium phosphate buffer, pH 8.0, mixed with the prepared lysates, and incubated at 4 °C, shaken, for 1 h. After incubation, resin was washed 2× with 10 mL of wash buffer 1 (50 mM sodium phosphate, 300 mM NaCl, 1% (v/v) Triton X-100, pH 8.0), 1× with 10 mL of wash buffer 2 (50 mM sodium phosphate, pH 8.0), and 2× with 10 mL of wash buffer 3 (50 mM sodium phosphate, 30 mM imidazole, pH 8.0). His-tagged proteins were eluted following 3 × 5-min incubations in 1.5 mL of elution buffer (50 mM sodium phosphate, 250 mM imidazole, pH 8.0). Combined elution fractions were dialyzed against wash buffer 2. The samples were quantified using the Bradford Assay and run on SDS-PAGE. The 6xHis-tags were then removed by incubating with thrombin protease, leaving a small Gly-Ser-His scar at the N-terminus.

### Protein structure determination

An additional size exclusion chromatography step was carried out immediately prior to crystallization to collect the dimeric species and buffer exchange the protein into 20 mM Tris, pH 8.0, 100 mM NaCl. Ligands were dissolved in DMSO and added to concentrated protein solutions to achieve a 5-fold molar excess of the ligand over SUR. Crystallization screening was carried out at ambient temperature using sitting-drop vapor diffusion with commercially available crystallization screens by mixing 1 μL of protein solution with 1 μL of precipitant solution and equilibrating against 100 μL of precipitant solution.

Crystals were grown and cryoprotected for X-ray data collection using the following conditions: EsR L7-D01 (6 mg/mL) crystallized from 1.6 M ammonium sulfate, 100 mM sodium citrate pH 5.5 and cryoprotected using 25% glycerol; EsR L11-C06-Es (10 mg/mL) crystallized from 200 mM ammonium sulfate, 100 mM sodium cacodylate pH 6.5, 30% w/v polyethylene glycol (PEG) 8,000 and cryoprotected using 20% ethylene glycol; CsR L4.2-20 (4.2 mg/mL) crystallized from 200 mM sodium acetate, 100 mM Tris pH 8.5, 30% PEG 4,000 and cryoprotected using 15% glycerol; CsR L4.2-20-Cs (6 mg/mL) crystallized from 50 mM ammonium sulfate, 50 mM Bis-Tris pH 6.5, 30% PEG 600 and cryoprotected using 10% glycerol.

X-ray diffraction data were collected at 100 K at beamline 4.2.2 of the Advanced Light Source or beamline 31-ID of the Advanced Photon Source. Diffraction data were reduced using d*Trek^104^ or MOSFLM^105^.

The structure of EsR L11-C06-Es was solved by molecular replacement (MR) using Phaser^106^ to search first for two dimers of the isolated ligand-binding domain (LBD). The search model employed was an ensemble of the LBDs of 16 unique dimers of class B or class D TetR structures (PDB IDs: 1A6I, 1BJ0, 1BJZ, 1DU7, 1ORK, 1QPI, 2NS7, 2NS8, 2O7O, 2TCT, 2TRT, 2VKE, 2VPR, and 2VKV) with non-identical side-chains truncated to C_γ_ or the last common atom. Consistent solutions were found for two dimers of the LBD, but subsequent molecular replacement searches for individual DBDs from single structures or ensembles failed. Inspection of difference electron density maps (*F*_o_−*F*_c_), calculated with the two-dimer LBD partial structure, allowed identification of a pattern of helical density matching one DBD, which was placed manually into the density and refined as a rigid body. Subsequent positional refinement of the partial structure comprising two LBD dimers and a single DBD allowed identification of the position of a second DBD in the difference electron density map, which was placed manually and refined. Two additional cycles of this process resulted in the correct placement of all four DBDs. At this stage, a clear positive difference density was visible for the Es in each LBD binding site.

The EsR L7-D01 apo structure was solved by MR using Phaser with the refined model of EsR L11-C06 as a search model. Consecutive MR solutions were found for one molecule of the LBD and one molecule of the DBD. The relatively high final R-factors for this model could not be improved, despite numerous efforts including refinement against multiple distinct datasets, refinement in lower symmetry space groups, and twinning refinement (no twinning was detected using standard tests). For this reason, no conclusions are drawn regarding the high-resolution atomic details of this structure, and it is employed only for qualitative comparisons with the EsR L11-C06-Es structure.

The CsR L4.2-20-Cs structure was solved by MR using Phaser to search for two copies of the EsR LBD dimer. The four corresponding DBDs were then placed manually into different electron density maps and rigid-body refined. The unliganded CsR L4.2-20 structure was similarly solved by MR searching for a single copy of the CsR LBD dimer from the liganded structure, then manually placing the two DBDs into difference density.

For all structures, iterative model building/adjustment in Coot^107^ and refinement in Refmac5^108^ within the CCP4 software suite^109^ led to the models described in Supplementary Table 2. All structural analyses, structure comparisons, and molecular graphics were performed using PyMOL (Schrödinger, LLC).

### Cloning of mammalian cell constructs

Human codon-optimized sequences of SUR LBDs were synthesized by Twist and inserted in place of the wtTetR LBD in the pcDNA6/TR (Tet Repressor) plasmid from the T-REx kit via Gibson assembly. The reporter sfGFP gene was inserted into the pcDNA4/TO (Tet Operator) vector of the kit via restriction cloning.

### Transient transfection of mammalian cells

HEK293 cells (ATCC ACS-4500) were plated in 24-well plates (30,000 cells per well). The next day, the cells were transfected with 0.5 μg of DNA mixture and 2 μL of TurboFect according to the manufacturer’s protocol. The DNA mixtures consisted of ~14:1 (w/w) pcDNA6/TR:pcDNA4/TO-sfGFP plasmid DNA ratio for experimental conditions and ~6:1 pcDNA4/TO:pcDNA4/TO-sfGFP plasmid DNA ratio for the single-stain control. Unstained/live-dead stain (propidium iodide, PI) single-stain control cells were treated similarly, but no DNA was added to the reaction mixture. Twenty-four hours after transfection, the medium was replaced with fresh medium supplemented with 1 μg/mL Tc, Es, Cs, or the appropriate amount of DMSO (carrier solvent, max 0.1%). Twenty-four hours after induction, the cells were harvested, resuspended in serum-free Opti-MEM with 3 μM PI, and analyzed by flow cytometry using a FACSymphony A1 Cell Analyzer (Becton Dickinson, FACSDiva v9.0.2). Data were analyzed using FlowJo 10.10.0.

### Fluorescence microscopy of mammalian cells

Fluorescence images of plates were taken on a Keyence BZ-X810 with a 10× objective and the Chroma 49002 EGFP fluorescence filter set (excitation: 470 nm/40 nm bandwidth; dichroic mirror 495 nm; emission: 525 nm/50 nm bandwidth) using BZ-X800 Viewer.

### Plant transformation vectors

Plant vectors were assembled using appropriate promoters, terminators, and selection markers for each species and application. All genes were plant codon-optimized. All vectors but PHP45473 were designed for random integration using *Agrobacterium* transformation and contained insertion flanked by T-DNA borders. Instead of T-DNA borders, vector PHP45473 has AscI restriction sites for excision and isolation of the DNA construct for particle-gun bombardment-based plant transformation. Vector designs are shown in Supplementary Table 3.

An inverted repeat of the TetR DBD without the ATG start codon was used as the anti-repressor siRNA (siRNA_REP_). These repeats were spaced by the potato *ST-LS1* gene intron IV2^110^.

### Soybean transformation

Wild-type plants of *Glycine max* (L.) Merrill cv. Jack were grown in growth chambers (Conviron) under 16/8 h photoperiod at 26/24 °C day/night temperatures in Redi-earth mix (Sun Gro Horticulture). Transgenic plants were produced from embryogenic cultures following the particle bombardment transformation protocol^111–113^. Briefly, soybean embryogenic suspension cultures were generated as described by Samoylov and colleagues^114^. The cultures were maintained in 250 mL flasks containing 50 mL of liquid media on rotary shakers at 26 °C under cool-white fluorescent lights with a 16/8 h day/night photoperiod. Fresh subcultures were bombarded with 0.6 μm gold particles coated with an 8.1 kb agarose gel-purified linear DNA fragment from the PHP45473 vector using the biolistic instrument PDS1000/HE (Bio-Rad). Transgenic events were selected with 100 ng/mL Cs under similar tissue culture conditions and analyzed for the copy number of inserted genes by qPCR. Single-copy transgenic plants were regenerated and maintained to maturity under the same conditions as the wild-type plants but in separate growth chambers.

Homozygous soybean seedlings harboring construct PHP45473 were treated with 20 mL Muster® dilutions containing 0.25, 0.5, or 1 mg of Es active ingredient or water. DsRed-Express fluorescence was visualized and quantified 6 days post-treatment.

### DsRed-Express protein quantification in soybean

Eight millimeter leaf punches were ground in 250 μL of Cell Culture Lysis Reagent (CCLR; 100 mM potassium phosphate, pH 7.8, 1 mM EDTA, 7 mM β-mercaptoethanol, 1% Triton X-100, 2 mM DTT, and 10% glycerol), and the homogenates were clarified by centrifugation. Fifty microliters of each clarified extract was placed into black-well, clear-bottom microtiter plates for fluorescence measurements. In parallel, samples for the standard curve were prepared using 0–50 ng/μL purified DsRed-Express protein (Clontech) in 50 μL of CCLR sample buffer. Fluorescence was then measured using a Typhoon Laser Scanning Imager (GE) set to excitation of 532 nm, emission at 580 nm with a 40 nm bandpass, and PMT of 300. Fluorescence reading of leaf extract from a non-transformed plant was subtracted from all plant samples. Readings were further normalized to total soluble protein concentration in each sample, determined by Bradford assay using BSA as the protein standard.

### Maize and rice transformation

Tissue culture media and transformation methods for maize and rice are described in Lowe et al.^47^. Briefly, immature embryos were isolated from the ears of greenhouse-grown plants at 9–12 days after pollination and incubated for 5 min in Murashige and Skoog (MS) medium^115^ with 68.5 g/L sucrose, 36 g/L glucose, 1.5 mg/L 2,4-dichlorophenoxyacetic acid (2,4-D), pH 5.8 containing *Agrobacterium* strain LBA4404 THY- (OD_550_ = 0.7) harboring the appropriate transformation vector. After that the embryos were cultured on solid MS medium containing 20 g/L sucrose, 10 g/L glucose, 2.0 mg/L 2,4-D, 100 μM acetosyringone, 50 mg/L thymidine, pH 5.8 for 3 days at 21 °C in the dark followed by culture on callus development medium (solid MS and Chu’s N6^116^ medium supplemented with 20 g/L sucrose, 0.6 g/L glucose, 0.8 mg/L 2,4-D, 1.2 mg/L dicamba, 100 mg/L carbenicillin) for 1 week at 26 °C in the dark, and on callus selection medium (solid MS and Chu’s N6 medium containing 20 g/L sucrose, 0.6 g/L glucose, 0.8 mg/L 2,4-D, 1.2 mg/L dicamba,100 mg/L cefotaxime, 150 mg/L Timentin (ticarcillin/clavulanic acid), pH 5.8 supplemented with 5 g/L maltose and 12.5 g/L mannose to select for PMI-containing transformants or 0.1 mg/L Es to select for HRA-containing transformants) for 2–2.5 months (subculturing every 2–3 weeks). After the selection, fluorescent calli were transferred onto regeneration medium (solid MS medium with 60 g/L sucrose, 0.5 mg/L zeatin, 0.1 mg/L thidiazuron, 1 mg/L 6-benzylaminopurine (BAP), 100 mg/L carbenicillin, pH 5.8 followed by hormone-free solid MS medium with 40 g/L sucrose) and cultured at 28 °C for 2–3 weeks. After the establishment of shoots and roots, plantlets were transferred to flats for 2 weeks for acclimation and then to pots for plant evaluation and seed production in a greenhouse.

### DsRed-Express protein and mRNA quantification in maize

Transgenic plants of Pioneer maize inbred PH184C hemizygous for a locus containing the derepressible DsRed-Express from vector PHP74334 were grown in the greenhouse and self-pollinated. Progeny were screened for zygosity of the DsRed-Express gene using qPCR. Both null and homozygous plants were grown to the V6 stage, and leaf punches were collected from the youngest leaf with a full collar. Punches were placed in multi-well plates containing either water or 1x Muster® (200 mg/L Es), vacuum-infiltrated for 5 min, and maintained in the light room with a 16/8 h light/dark cycle over 3 days at 28 °C. Tissue was collected at several time points for both RNA and protein concentration analysis.

DsRed-Express protein was quantified from leaf tissue collected before the treatment as well as 24, 48, and 72 h after vacuum-infiltration using an Octet Red 96 biolayer interferometer (ForteBio). Leaf discs (4 per sample) from each time point were pooled, frozen, and extracted in 800 μL phosphate-buffered saline, pH 7.4, with 0.05% Tween-20 using a Geno/Grinder (SPEX SamplePrep) set to 1650 rpm for 1 min. Extracts were centrifuged for 10 min at 4000 × *g*, and the supernatants were collected. Total protein concentration was determined via Bradford assay, and data were used to normalize all samples to 200 μg/mL protein concentration. Ten micrograms per microliter of Clontech anti-DsRed-Express polyclonal antibody (Product #632496) were loaded onto Protein G biosensors, and the normalized leaf extract samples or DsRed-Express purified protein standards in the same buffer were bound to the antibody-laden sensor tips. A standard curve was prepared by fitting the initial binding rate slopes of the purified DsRed-Express protein samples with an unweighted 4-parameter logistic regression model and used for determining the DsRed-Express concentrations in the leaf extracts.

DsRed-Express transcript levels were determined using quantitative reverse transcription polymerase chain reaction (qRT-PCR) relative to a constitutive internal control gene. For that, 600 ng of leaf tissue was frozen at −80 °C for each of the data points, and RNA was later prepared using a Qiagen RNeasy kit followed by DNaseI treatment. Sequence-specific forward and reverse primers and a FAM-labeled probe were designed for DsRed-Express, and sequence-specific forward and reverse primers and a VIC-labeled probe were designed for a moderately expressing endogenous gene for multiplexed reactions. Assay conditions were optimized, and expression of DsRed-Express was calculated relative to the endogenous expression standard.

### Tobacco transformation

Axillary buds of tobacco (*Nicotiana tabacum* cv. Xanthi NN) were sub-cultured on half-strength MS with 1.5% sucrose and 0.3% Gelrite under 16/8 h day/night cycle (65–80 mE m^−2^ s^−1^, cool-white fluorescent lamps) at 24 °C every 3–4 weeks. Tobacco plants were transformed by *A. tumefaciens* strain EHA105 harboring plant transformation vectors containing a selectable marker according to the modified method of Fisher and Guiltinan^117^. Young leaves were excised from plants after 2–3 weeks, sub-cultured, and cut into 3 × 3 mm segments. *A. tumefaciens* EHA105 strains were inoculated into LB medium with 100 μg/mL kanamycin and grown overnight to a density of *A*_600_ = 1.0. Cells were pelleted at 3200 × *g* for 5 min and resuspended in 3 volumes of liquid co-cultivation medium composed of MS medium (pH 5.2) with 2 mg/L N^6^-benzyladenine (BA), 1% glucose, and 400 mM acetosyringone. Leaf pieces were then fully submerged in 20 mL of *A. tumefaciens* suspension in 100 × 25 mm Petri dishes for 5 min, blotted with autoclaved filter paper, then placed on solid co-cultivation medium and incubated as described above. After 3 days of co-cultivation, 20–30 segments were transferred to basal shoot induction medium composed of MS solid medium (pH 5.7) supplemented with 2 mg/L BA, 3% sucrose, 0.3% Gelrite, 200 mg/L Timentin (ticarcillin/clavulanic acid; to suppress excess *Agrobacterium* growth^118^), and selection agent (50 ng/mL imazapyr or 100 mg/L kanamycin). Co-cultivated leaf tissue was transferred to fresh shoot induction medium every 3 weeks. Regenerated shoots were isolated and transferred to growth regulator-free half-strength MS solid medium (pH 5.7) containing 1.5% sucrose, 0.3% Gelrite, 200 mg/L Timentin, and the selection agent for shoot elongation and rooting.

### Transgenic tobacco leaf disks derepression assay

Duplicate leaf disks from rooted imazapyr-resistant tobacco plantlets transformed with either pVER7384 or pVER7385 were placed on MS agar with Es at the indicated concentration and incubated at 25 °C for 3 days under 16/8 h light/dark cycle, during which the samples were inspected daily for DsRed-Express expression. All photos were taken with a 0.5 s exposure and arranged in a 9 × 9 contact-sheet format using XnView software with brightness set to 9, contrast to 22, and gamma to 2. Original unprocessed image files are no longer available; therefore, these data are presented qualitatively.

### *Arabidopsis* transformation

*Arabidopsis thaliana* was transformed using a modification of the floral dip method^119^, whereby plants are sprayed with *Agrobacterium* transformation strains resuspended to an OD_600_ of 0.5 in a solution of 10 mM MgSO_4_ and 0.02% Silwet L-77 (silicone surfactant). T0 seeds harvested from sprayed plants were collected and plated onto Whatman filter paper soaked with 200 ng/mL Ci. Germinated seeds exhibiting DsRed-Express expression were transplanted to soil, and second-generation T1 seeds were collected.

### Insecticidal assay

Eight 6 mm leaf disks were excised from each tested transgenic tobacco plant and placed into 48-well microtiter plate wells containing 200 μL MS medium with or without 100 ng/mL Es for 3 days to induce expression of proteins under the control of the 35S-3xOp promoter, including AcGFP (transgenic plants containing pHD4157 vector) or AcGFP-IP2-127 fusion (transgenic plants containing pHD4155 vector). Simultaneously, *Helicoverpa zea* larvae were fed on an artificial diet until they reached the 2nd instar. Individual larvae were then transferred to each of the leaf disks. Plates were covered with a clear tape seal, perforated to enable gas exchange, and incubated for 48 h prior to imaging.

### *N. benthamiana* transformation and leaf fluorescence quantitation assay

*Nicotiana benthamiana* cv. TW17 (US Nicotiana Germplasm Collection, North Carolina State University) was grown in 3″ pots in the growth chamber under 16/8 h day/night photoperiod, at 25 °C. An *Agrobacterium*-based leaf infiltration^120^ assay was used to rapidly measure repression and derepression activities of engineered constructs *in planta*. Recombinant *Agrobacterium* strains with vectors pVER7384, pHD2094, pHD1120, and pHD2096 were mixed at a ratio of 9:1 with an *Agrobacterium* strain harboring a constitutively expressed ZsGreen control and pressure co-infiltrated into attached leaves—all four per each leaf (6 leaves total). After 24 h, the soil at the base of each plant was treated with either 20 mL of 1x Muster® solution (200 μg/mL Es) or water. Three days later, the leaves were excised and placed abaxial side down onto a Typhoon Laser Imager (GE), and fluorescence was measured in each infiltrated area using an excitation of 532 nm, emission of 580 nm with a 40 nm bandpass, and PMT set to 300 for DsRed-Express, and excitation and emission set to 488 and 520 nm for ZsGreen. Fluorescence reading from a non-transformed leaf was subtracted from all samples.

### Quantification of transgene copy number by qPCR

Transgene DNA copy number was estimated by qPCR^51^. DNA was extracted from 200 ng fresh leaf tissue via a modified alkaline lysis method HotSHOT^121^. Genes encoding DsRed-Express and/or HRA were quantified using sequence-specific forward and reverse primers and fluorogenic probes (FAM and MGB-based, correspondingly). Each assay was multiplexed and normalized with an endogenous gene using sequence-specific forward and reverse primers and a VIC-labeled probe. Each multiplexed assay was primer-titrated to near equal efficiencies, and reactions for test and endogenous genes were run simultaneously in a single-tube optimized reaction. Upon completion of real-time qPCR, all raw data were used to calculate the ΔCT, and copy numbers were determined using the 2^−ΔΔCT^ method^122^.

### Plant imaging

All plants were imaged with a Leica M165 fluorescent stereoscope fitted with a DsRed filter set and CCD camera. For plants in a comparison group, all photographs were taken with identical exposure times and camera settings.

### Herbicide application

Dosages of test SU herbicides were set at 4× (800 mg/L), 2× (400 mg/L), 1× (200 mg/L), 0.5× (100 mg/L), or 0.25× (50 mg/L) active ingredient. Surfactant Optima (Helena Agri-Enterprises, LLC) was included in each solution at 2.5 μL/mL. Spray application was carried out at a flow rate of 100 L/Ha at 2 mph and 18 inches above the tops of test plants using a Generation III Research Sprayer (DeVries Manufacturing). These settings deliver 20 g of active ingredient per Ha when using a 1x herbicide solution. Following treatment, plants were not watered for 1 day, and then watered from the base of the plant thereafter to enable full penetration of the herbicide.

For soak treatment, 20–30 mL of SU solutions without surfactant were added to the soil surrounding seedlings in 4″ pots. Following treatment, plants were not watered for 1 day, and then were watered from the base of the plant thereafter to enable full penetration of the herbicide. Plants, along with soil plugs from 4″ pots, were then rinsed, and the soil plug transplanted to 2-gallon pots.

### Reporting summary

Further information on research design is available in the Nature Portfolio Reporting Summary linked to this article.

## Supplementary information

Supplementary Information

Peer Review file

Description of Additional Supplementary Files

Supplementary Data 1

Supplementary Data 2

Supplementary Data 3

Supplementary Data 4

Supplementary Data 5

Supplementary Data 6

Supplementary Data 7

Supplementary Data 8

Supplementary Data 9

Supplementary Data 10

Supplementary Data 11

Supplementary Data 12

Supplementary Data 13

Supplementary Data 14

Supplementary Data 15

Supplementary Data 16

Supplementary Data 17

Supplementary Data 18

Supplementary Data 19

Reporting Summary

## Source data

Source data

## Data Availability

The crystal structures of EsR L7-D01 apo, EsR L11-C06-Es, CsR L4.2-20 apo, and CsR L4.2-20-Cs generated in this study have been deposited in the PDB under accession 9DT2, 9DT3, 9DT4, and 9DT5, respectively. Previously published structures used in this study are available in the PBD under accession codes 1A6I, 1BJ0, 1BJZ, 1DU7, 1N0H, 1ORK, 1QPI, 1T9A, 1T9B, 1T9C, 1T9D, 1YBH, 1YHY, 1YHZ, 1YI0, 1YI1, 2NS7, 2NS8, 2O7O, 2TCT, 2TRT, 2VKE, 2VPR, 2VKV, 3E9Y, 3EA4, 4AC0, 5FEM, 6DEL, 6DEM, 6DEN, 6DEP, 7STQ, 7U1D, 7Y0L, 7YD2, 8GOL, 8IVE, 8IVM, 8IVN, 8IVS, 8IVT, 8IW3, 8IW6, 8J7I, and 8J7L. Bacterial pRSET-based vectors encoding N-terminal 6xHis-tagged wtTetR, L12-11, L13-9, L13-23, L15-20, CsL4.2-15, and CsL4.2-20 as well as mammalian vectors pcDNA6/L13-9, pcDNA6/L15-20, pcDNA6/CsL4.2-15, pcDNA6/CsL4.2-20, and pcDNA4/TO-sfGFP. Contact L.L.L. (llooger@ucsd.edu) for additional bacterial and mammalian plasmid requests. Requests will receive a response within 14 days. Corteva Agriscience will provide plant plasmids to academic investigators for non-commercial research under an applicable material transfer agreement, subject to proof of permission from any third-party owners of all or parts of the material and to governmental regulation considerations. Completion of a stewardship plan is also required. Contact M.A. (maren.arling@corteva.com) for plant vectors. Requests will be answered within 30 days. Source data are provided with this paper.

## Code availability

Code for running computational protein modeling and design software Chameleon and the ImageJ script can be found in the Looger lab GitHub repository [https://github.com/LorenLoogerUCSD/SURepressors]. A snapshot of the code is also provided on Zenodo^123^.
