# Supplementary material for: Design and evolution of the tetracycline repressor into sulfonylurea herbicide-responsive gene switches for field crops
Source: Nat Commun. 2026 Jun 8;17:7278. doi: 10.1038/s41467-026-73848-w (PMC13402354; doi:10.1038/s41467-026-73848-w)
Supplement: Supplementary file 1 — Supplementary Information [file 41467_2026_73848_MOESM1_ESM.docx]

**Design and evolution of the tetracycline repressor into sulfonylurea herbicide-responsive gene switches for field crops**

McBride, Kakani and Bozhanova *et al.*

Supplementary Table 1. Physicochemical properties of SU herbicides and (anhydro)tetracycline.

| **Compound;**  **registered trademark** | **MW (g/mol)** | **logP** | **pKa** | **pI** | **Approved crops** |
| --- | --- | --- | --- | --- | --- |
| Anhydrotetracycline (Atc) | 426.4 | 1.2 | 3.5, 8.3 | 5.9 | N/A |
| Tetracycline (Tc) | 444.4 | -0.6 | 3.3, 9.0 | 5.2 | N/A |
| Chlorimuron-ethyl (Ci);  Classic^®^ | 414.8 | 2.9 | 4.8 | 2.2 | Soy, peanut, field corn |
| Chlorsulfuron (Cs);  Glean^®^ | 357.8 | 2.6 | 4.3 | 2.7 | Cereals (barley, oat, sorghum, millet), soybean, cotton |
| Ethametsulfuron-methyl (Es);  Muster^®^ | 410.4 | 2.1 | 4.8 | 3.6 | Canola |
| Metsulfuron-methyl (Ms);  Ally^®^ | 381.4 | 2.1 | 4.8 | 3.0 | Cereals, pasture, trees |
| Nicosulfuron (Ns);  Accent^®^ | 410.4 | 0.8 | 5.0 | 2.8 | Corn (field, sweet, pop-), hay |
| Rimsulfuron (Rs);  Matrix^®^ | 431.4 | 0.8 | 4.9 | 2.6 | Corn, tomato, potato, grape, citrus, nuts, fruits, roots |
| Sulfometuron-methyl (Sm);  Oust^®^ | 364.4 | 1.5 | 4.8 | 4.0 | Vegetation management |
| Thifensulfuron-methyl (Ts);  Harmony^®^ | 387.4 | 2.0 | 5.2 | 3.2 | Cereals (barley, sorghum, flax), corn, soy, rice, sugar beet |
| Tribenuron-methyl (Tb);  Express^®^ | 395.4 | 2.0 | 4.7 | 2.3 | Cereals (barley, alfalfa, sorghum, flax, triticale, wheat), sunflower, peas, beans, rice, berries, vegetables, potato, tobacco, tomato |

Supplementary Table 2. X-ray data collection and refinement statistics for SUR crystal structures.

|  | **EsR L7-D01 apo**  **PDB 9DT2** | **EsR L11-C06-Es**  **PDB 9DT3** | **CsR L4.2-20 apo**  **PDB 9DT4** | **CsR L4.2-20-Cs**  **PDB 9DT5** |
| --- | --- | --- | --- | --- |
| **Data collection** |  |  |  |  |
| Space group | P4_1_2_1_2 | P2_1_ | P2_1_ | P2_1_2_1_2_1_ |
| Unit cell dimensions |  |  |  |  |
| a, b, c (Å) | 72.4, 72.4, 95.2 | 65.8, 71.0, 90.5 | 37.1, 56.8, 106.0 | 63.0, 109.1, 129.2 |
| α, β, γ (°) | 90, 90, 90 | 90, 92.2, 90 | 90, 90.3, 90 | 90, 90, 90 |
| Resolution (Å) | 45.11-3.10 (3.31-3.10) | 30-2.80 (2.90-2.80) | 20-2.40 (2.53-2.40) | 20-2.00 (2.11-2.00) |
| *R*_sym_^a^ | 0.122 (0.583) | 0.105 (0.462) | 0.120 (0.616) | 0.128 (0.578) |
| I / σI^b^ | 12.8 (3.4) | 7.9 (2.3) | 10.5 (2.5) | 12.8 (4.3) |
| Completeness (%)^b^ | 100 (100) | 99.6 (99.4) | 95.1 (73.3) | 99.7 (99.8) |
| Redundancy^b^ | 7.5 (7.7) | 5.6 (5.7) | 5.4 (4.4) | 10.7 (10.3) |
| **Refinement** |  |  |  |  |
| Resolution (Å) | 3.10 | 2.80 | 2.4 | 2.00 |
| Number of reflections | 4694 | 19165 | 16581 | 57672 |
| *R*_work_/*R*_free_ (%)^c^ | 23.7/29.8 | 19.0/27.8 | 18.2/24.1 | 17.8/23.4 |
| Number of atoms |  |  |  |  |
| Protein/ligands/water | 1365/0/0 | 6041/112/12 | 3201/0/17 | 6405/92/414 |
| *B*-factors (Å^2^) |  |  |  |  |
| Protein/ligands/water | 64.7/-/- | 29.7/37.4/15.7 | 36.9/-/28.8 | 27.5/26.8/32.0 |
| RMSD values |  |  |  |  |
| Bonds (Å)/angles (°) | 0.006/1.745 | 0.014/1.490 | 0.008/1.788 | 0.024/1.826 |
| Ramachandran plot (%) |  |  |  |  |
| Favored/allowed/disallowed | 87.4/8.4/4.2 | 94.3/5.0/0.7 | 99.24/0.76/0 | 99.75/0.25/0 |
| Molprobity Clashscore (percentile) | 16.72 (96) | 4.49 (100) | 3.27 (100) | 3.89 (99) |
| Molprobity score (percentile) | 3.08(76) | 2.28 (97) | 1.76 (98) | 1.63 (93) |

^a^*R*_sym_ = Σ*^i^_hkl_* |*I^i^*_(_*_hkl_*_)_ - <*I*_(_*_hkl_*_)_>| / Σ*_hkl_* <*I*_(_*_hkl_*_)_>, where *I^i^*_(_*_hkl_*_)_ is the *i^th^* measured diffraction intensity and <*I*_(_*_hkl_*_)_> is the mean of the intensity for the Miller index (*hkl*).

^b^The number in parentheses is for the highest resolution shell.

^c^*R*_work_ = Σ*_hkl_* ||*F*_o_(*hkl*)| - |*F*_c_(*hkl*)|| / Σ*_hkl_* |*F*_o_(*hkl*)|. *R*_free_ = *R*_work_ for 5% of reflections not included in refinement.

Supplementary Table 3. Vector designs and transformation statistics.

| **Vector name** | **Vector insertion design** | **Target species** |
| --- | --- | --- |
| PHP45473 | 35S-3xOp_pro_:DsRed-Express:_term_CaMV 35S + GmEF1a2_pro_:EsR(L15-20):_term_CaMV 35S + GmSAMS_pro_:HRA:_term_GmAls | *Glycine max* (L.) Merrill cv. Jack |
| PHP74334 | RB + 35S-3xOp_pro_:DsRed-Express:_term_PinII + SbALS_pro_:HRA:_term_SbPEPC1 + ZmUbi_pro_:EsR(L15-20):_term_SbSAG12 + ZmUbi_pro_:PMI:_term_PinII + LB | Pioneer *Zea mays* inbred PH184C |
| PHP63015 | RB + Nos-1xOp_pro_:ZmWUS2:_term_PinII + ZmUbi-3xOp_pro_:ZmBBM:_term_PinII + ZmUbi_pro_:EsR(L15-20):_term_PinII + SbALS_pro_:HRA:_term_PinII + CaMV 35S enhancer-HvLTP2_pro_:ZsYellow:_term_PinII + LB | Pioneer *Zea mays* inbred PH184C |
| PHP60850 | RB + ZmGz_pro_:amCyan:_term_ZmGz + Nos-1xOp_pro_:ZmWUS2:_term_PinII + ZmUbi-3xOp_pro_:ZmBBM:_term_PinII + ZmUbi_pro_:EsR(L15-20):_term_PinII + SbALS_pro_:HRA:_term_PinII + LB | *Oryza* *sativa* cv. Kitaake |
| PHP32269 | RB + ZmUbi_pro_:PMI:_term_PinII + ZmUbi_pro_:moPAT-ZsYellow:_term_PinII + LB | *Oryza* *sativa* cv. Kitaake with PHP60850 |
| pVER7384 | RB + 35S-3xOp_pro_:DsRed-Express:_term_UBQ3 + 35S-3xOp_pro_:EsR(L13-23):_term_UBQ14 + GmSAMS_pro_:HRA:_term_GmAls + LB | *Nicotiana tabacum* cv. Xanthi NN |
| pVER7385 | RB + 35S-3xOp_pro_:DsRed-Express:_term_UBQ3 + 35S_pro_:EsR(L13-23):_term_UBQ14 + GmSAMS_pro_:HRA:_term_GmAls + LB | *Nicotiana tabacum* cv. Xanthi NN |
| pVER7384 | RB + 35S-3xOp_pro_:DsRed-Express:_term_UBQ3 + 35S-3xOp_pro_:EsR(L13-23):_term_UBQ14 + GmSAMS_pro_:HRA:_term_GmAls + LB | *Arabidopsis thaliana* |
| pHD4155 | RB + 35S-3xOp_pro_:acGFP-IP2-127:_term_UBQ3 + AtEF1a_pro_:NptII:_term_AtEF1a + LB | wild-type *N. tabacum* with pHD1180 |
| pHD4157 | RB + 35S-3xOp_pro_:acGFP:_term_UBQ3 + AtEF1a_pro_:NptII:_term_AtEF1a + LB | wild-type *N. tabacum* with pHD1180 |
| pHD1180 | RB + 35S-3xOp_pro_:DsRed-Express:_term_UBQ3 + 35S-1xOp_pro_:EsR(L13-23):_term_UBQ14 + GmSAMS_pro_:HRA:_term_GmAls + LB | *Nicotiana tabacum* cv. Xanthi NN |
| pHD1198 | RB + 35S-3xOp_pro_:DsRed-Express:_term_UBQ3 + 35S-1xOp_pro_:EsR(L13-23):_term_UBQ14 + MMV-3xOp_pro_:siRNA_REP_:_term_PinII + GmSAMS_pro_:HRA:_term_GmAls + LB | *Nicotiana tabacum* cv. Xanthi NN |
| pVER7384 | RB + 35S-3xOp_pro_:DsRed-Express:_term_UBQ3 + 35S-3xOp_pro_:EsR(L13-23):_term_UBQ14 + GmSAMS_pro_:HRA:_term_GmAls + LB | *N. benthamiana* cv. TW17 |
| pHD1120 | RB + 35S-3xOp_pro_:DsRed-Express:_term_UBQ3 + 35S-3xOp_pro_:EsR(L15-20):_term_UBQ14 + GmSAMS_pro_:HRA:_term_GmAls + LB | *N. benthamiana* cv. TW17 |
| pHD2094 | RB + 35S-3xOp_pro_:DsRed-Express:_term_UBQ3 + 35S-3xOp_pro_:EsR(L13-23)-SP:_term_UBQ14 + GmSAMS_pro_:HRA:_term_GmAls + LB | *N. benthamiana* cv. TW17 |
| pHD2096 | RB + 35S-3xOp_pro_:DsRed-Express:_term_UBQ3 + 35S-3xOp_pro_:EsR(L15-20)-SP:_term_UBQ14 + GmSAMS_pro_:HRA:_term_GmAls + LB | *N. benthamiana* cv. TW17 |

RB - right border T-DNA repeat

LB - left border T-DNA repeat

acGFP^1^ - *Aequorea coerulescens* green fluorescent protein, a reporter

amCyan - *Anemonia majano* cyan fluorescent protein, a reporter

DsRed-Express^2^ - red fluorescent protein, a reporter

EsR(L13-23), EsR(L15-20) - sulfonylurea (Ethametsulfuron-methyl) repressors designed in this study

HRA - high-resistance allele, a selectable marker

IP2-127 - Cry2A Bt toxin IP2-127^3^

moPAT - maize-optimized phosphinothricin acetyltransferase structural gene^4^

NptII - neomycin phosphotransferase II, a selectable marker

PMI - phosphomannose isomerase, a selectable marker

siRNA_REP_ - short inhibitory RNA targeting the repressor

SP - 6-amino acid silencer peptide (TLLLFR) from *Arabidopsis* transcription factor AtMYBL2^5^

ZmWUS2 - *Zea mays* morphogenic factor Wuschel2

ZmBBM - *Zea mays* morphogenic factor Baby boom

ZsYellow^6^ - yellow fluorescent protein, a reporter

CaMV 35S enhancer - Cauliflower Mosaic virus 35S enhancer

35S_pro_ - constitutive cauliflower mosaic virus 35S promoter^7^

35S-1xOp_pro_ - cauliflower mosaic virus 35S promoter with one *TetO* sequence inserted, an inducible promoter

35S-3xOp_pro_ - cauliflower mosaic virus 35S promoter with three *TetO* sequences inserted^8^, an inducible promoter

AtEF1a_pro_ - constitutive *Arabidopsis thaliana* translation elongation factor *EF1A2* promoter

GmEF1a2_pro_ - constitutive *Glycine max* translation elongation factor *EF1A2* promoter^9^

GmSAMS_pro_ - constitutive *Glycine max* S-adenosylmethionine synthase promoter

HvLTP2_pro_ - constitutive *Hordeum vulgare* putative lipid transfer 2 protein promoter^10^

MMV-3xOp_pro_ - Mirabilis Mosaic virus promoter with three *TetO* sequences inserted, an inducible promoter

Nos-1xOp_pro_ - *Agrobacterium* nopaline synthase promoter with one *TetO* sequence inserted, an inducible promoter

SbALS_pro_ - constitutive *Sorghum bicolor* acetolactate synthase promoter

ZmGz_pro_ - constitutive *Zea mays* gamma zein promoter

ZmUbi_pro_ - constitutive *Zea mays* ubiquitin 1 promoter

ZmUbi-3xOp_pro_ - *Zea mays* ubiquitin 1 promoter with one *TetO* sequence inserted, an inducible promoter

_term_AtEF1a - *Arabidopsis thaliana* translation elongation factor *EF1A2* terminator

_term_CaMV 35S - Cauliflower Mosaic virus 35S terminator

_term_GmAls - *Glycine max* acetolactate synthase terminator

_term_PinII - potato proteinase inhibitor II terminator^11^

_term_SbPEPC1 - *Sorghum bicolor* phosphoenolpyruvate carboxylase 1 terminator

_term_SbSAG12 - *Sorghum bicolor* senescence-associated gene *SAG12* terminator

_term_UBQ3 - *Arabidopsis thaliana* polyubiquitin 3 terminator^12^

_term_UBQ14 - *Arabidopsis thaliana* polyubiquitin 14 terminator^12^

_term_ZmGz - *Zea mays* gamma zein terminator

Supplementary Table 4. Phenotypes of KM3 cells ± functional TetR ± tetracycline (Tc).

|  | PtetA::lacZ::aadA  lacZ = β-galactosidase  aadA = spectinomycinR | PtetR::lacI  lacI = lac repressor | Plac::nptIII  nptIII = kanamycinR |
| --- | --- | --- | --- |
| KM3 strain | Z^+^, Sp^r^ | lacI^+^ | Kan^s^ |
| Functional TetR, no Tc | Z^-^, Sp^s^ | lacI^-^ | Kan^r^ |
| non-DNA-binding TetR mutant | Z^+^, Sp^r^ | lacI^+^ | Kan^s^ |
| Functional TetR + Tc | Z^+^, Sp^r^ | lacI^+^ | Kan^s^ |


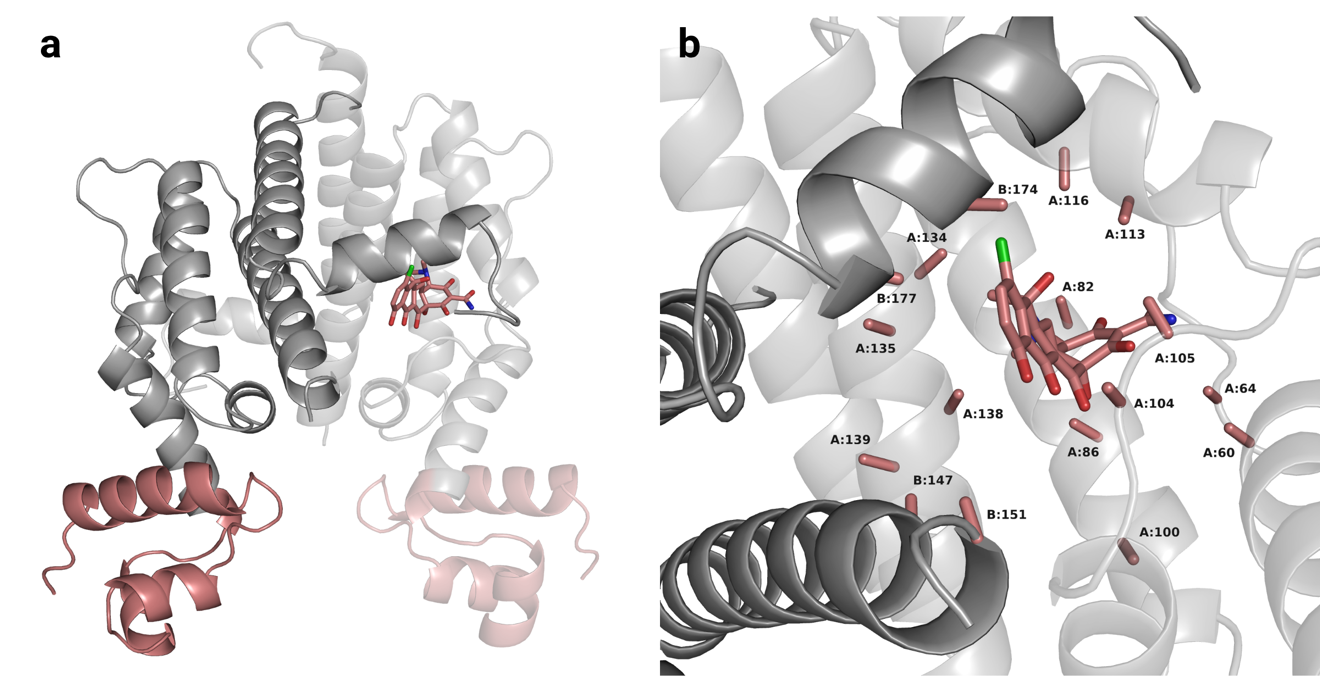


Supplementary Fig. 1. Structure of the class D TetR in complex with 7-chlortetracycline (PDB 1DU7).

a Overall structure of the TetR dimer. The protein is shown as cartoon. LBDs are colored gray; DBDs are colored salmon. 7-chlortetracycline is shown as salmon sticks and bound at the interface of two chains (light and dark). Only one out of two ligand molecules is shown for simplicity. b Close-up of the ligand-binding pocket. Side chains of amino acids at 17 positions chosen for computational design are trimmed to alanine and shown as salmon sticks. Labels show chain identity and amino acid position.


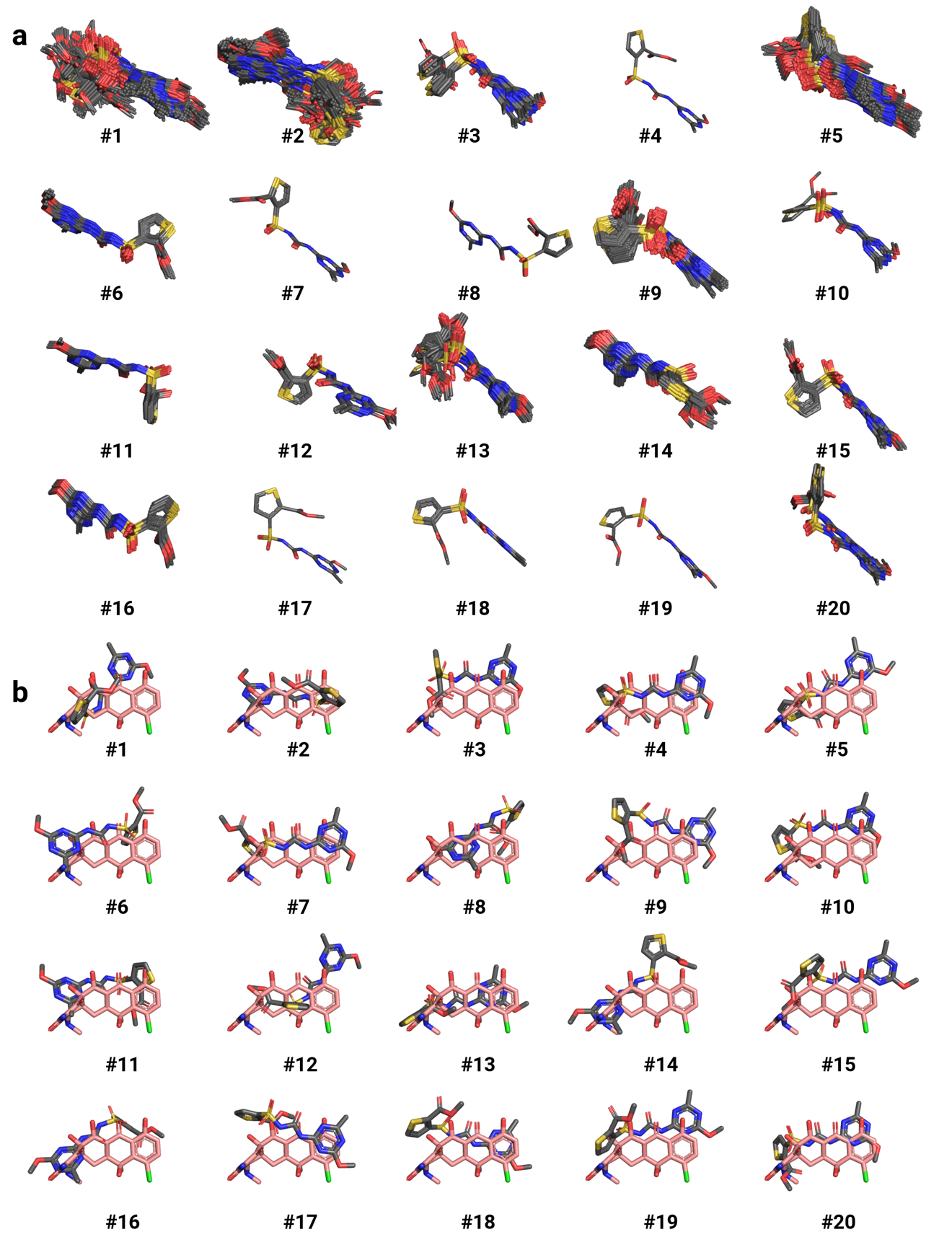


Supplementary Fig. 2. Clustering of 1,811 Ts placements deemed possibly compatible with the TetR scaffold. a All ligand structures assigned to 20 clusters. b Position of the representative pose from each cluster (gray) relative to the 7-chlortetracycline (salmon) from 1DU7.


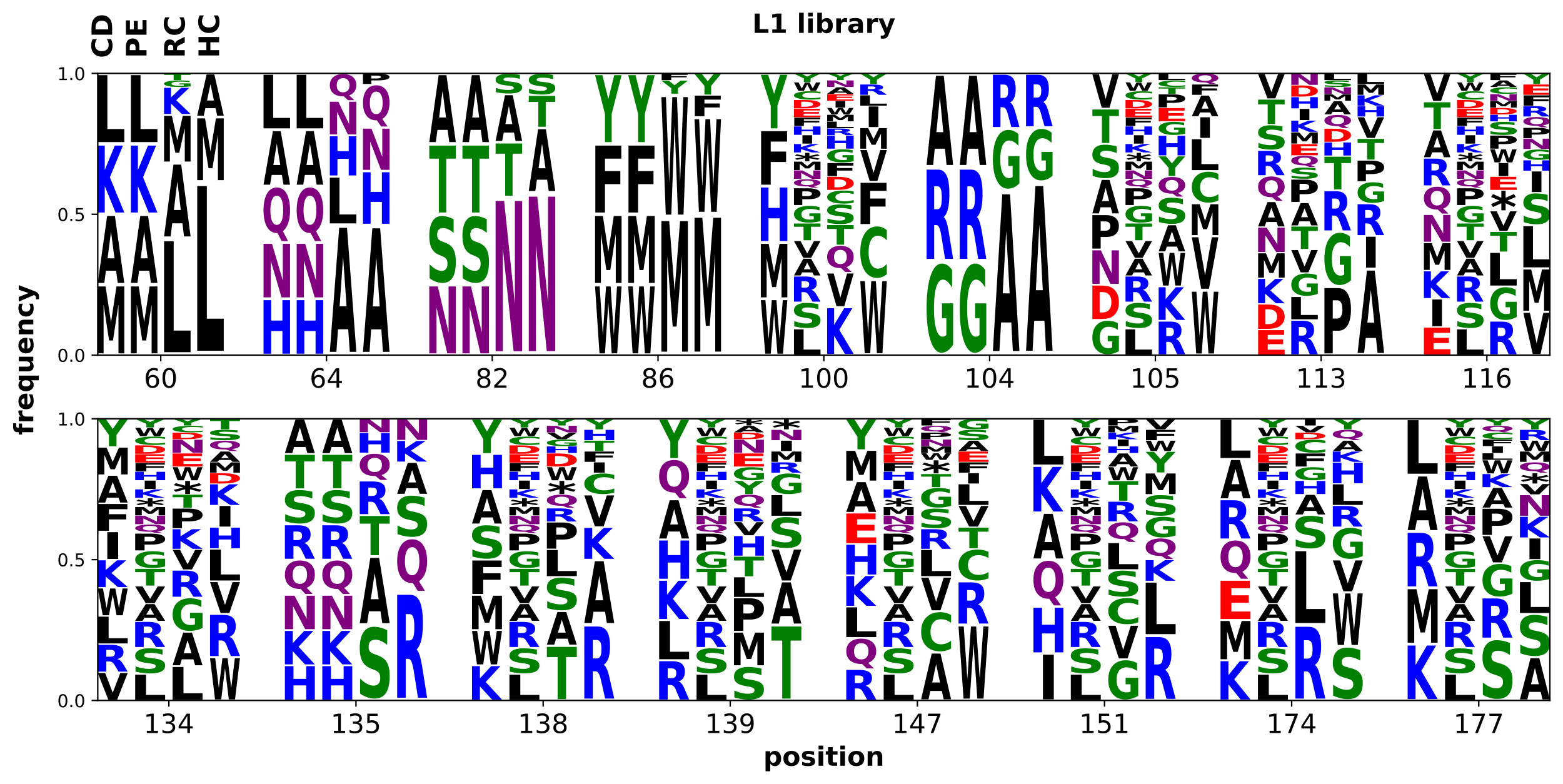


Supplementary Fig. 3. Round 1 library design, construction, and screening results. For each of the 17 mutated positions, shown are amino acid frequencies of the computationally designed library (column 1, CD), primer-encoded library (column 2, PE), randomly picked clones (column 3, RC), and selected hits (column 4, HC).

**
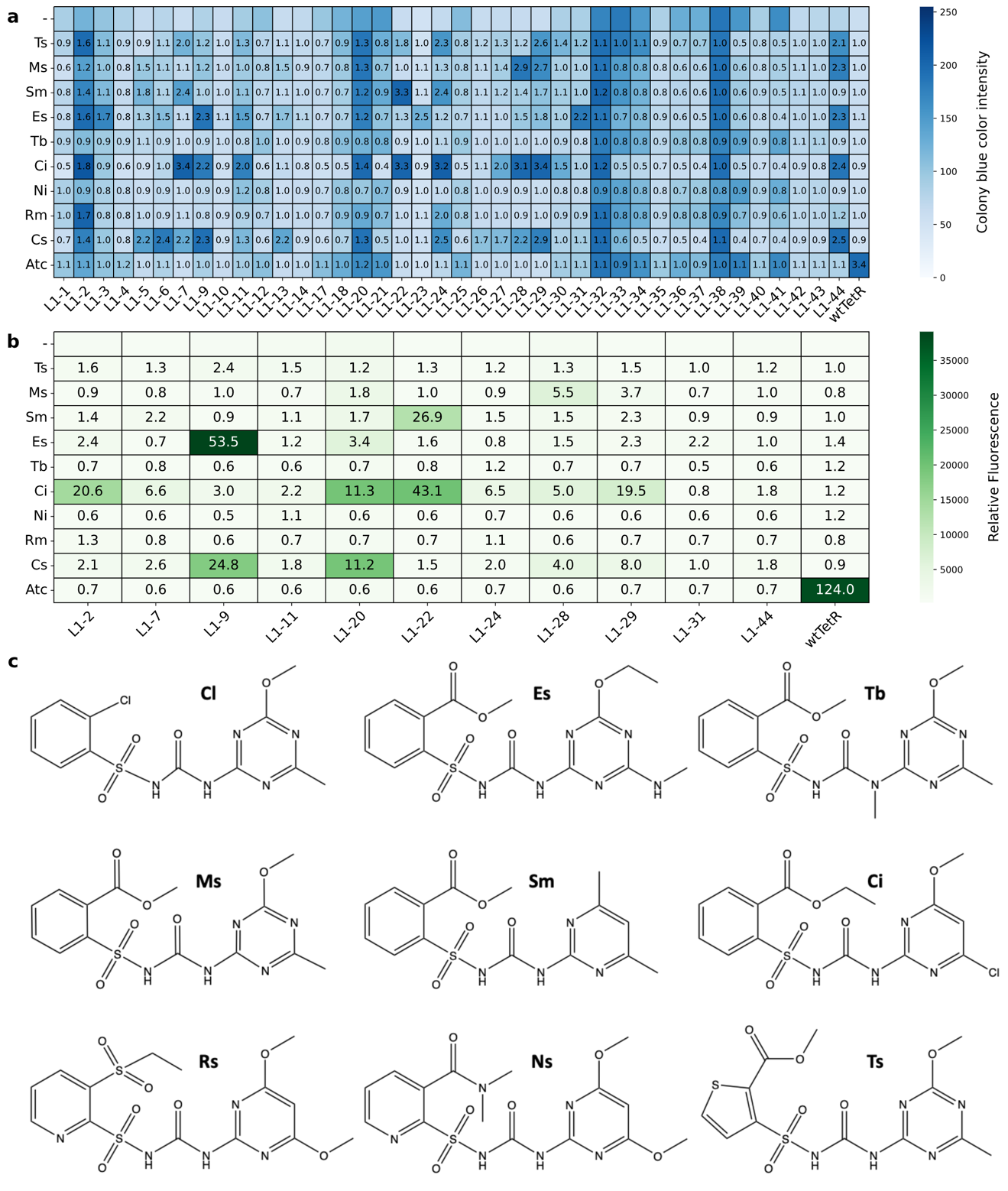
**

Supplementary Fig. 4. Derepression of 1^st^ round hits by nine SUs and Atc. a,b β-gal activity of KM3 cells expressing an L1 library hit or wild-type TetR assessed (a) based on intensity of the blue color of the colonies after 60 hours of incubation on plates or (b) in bacterial lysate using fluorogenic substrate 4-methylumbelliferyl-β-D-galactopyranoside (MUG) after 2 hours of incubation with chlorsulfuron (Cs), rimsulfuron (Rs), nicosulfuron (Ns), chlorimuron-ethyl (Ci), tribenuron-methyl (Tb), ethametsulfuron-methyl (Es), sulfometuron-methyl (Sm), metsulfuron-methyl (Ms), thifensulfuron-methyl (Ts) (all at 10 μg/mL (~25 μM)), anhydrotetracycline (atc) at 4 μM, or without any ligand (-). Numbers in cells represent the ratio of β-gal activity in the presence of the compound *vs.* in the absence of any ligand. c Chemical structures of the SU compounds used in screening.


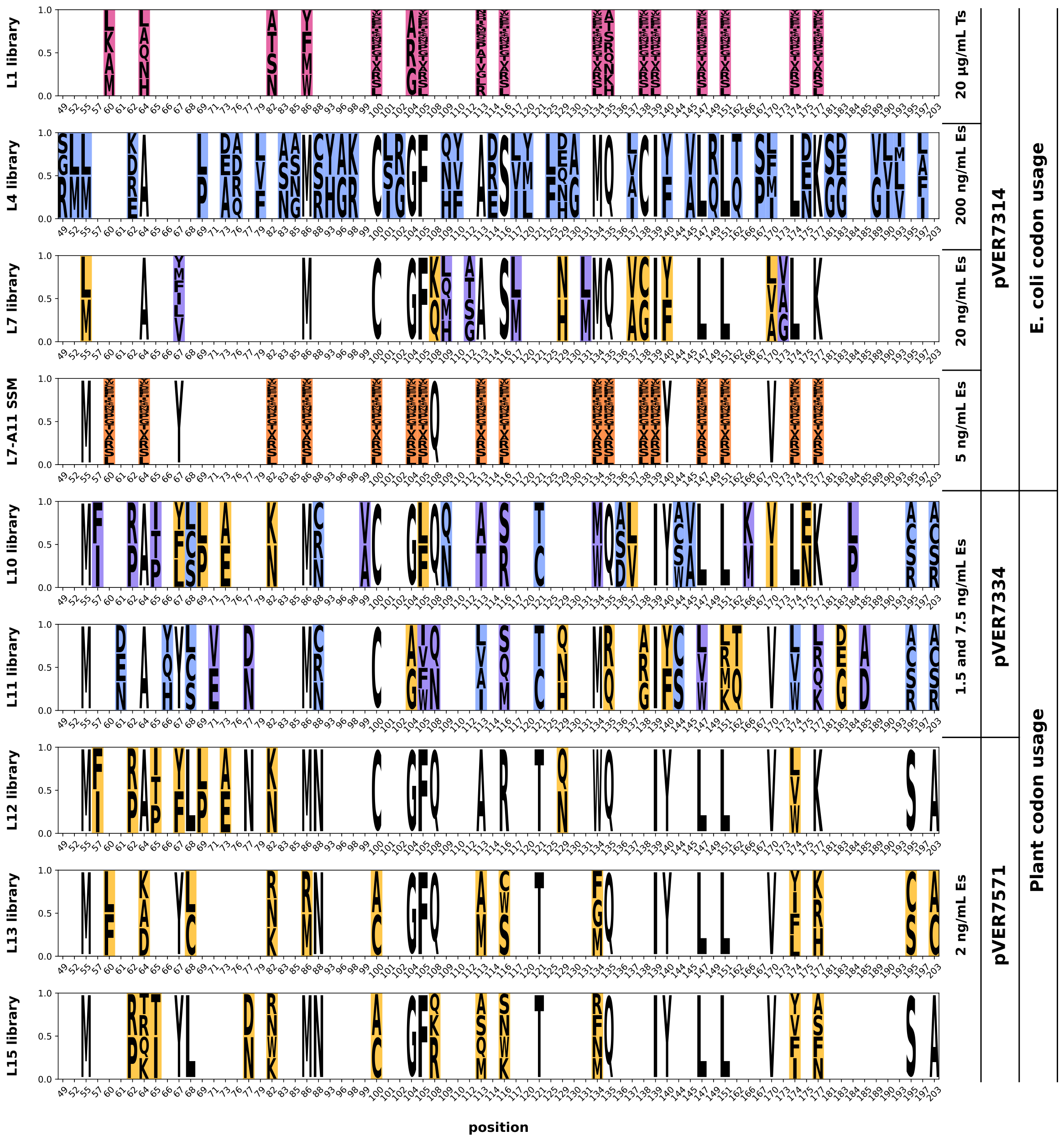


Supplementary Fig. 5. Summary of the design process of EsRs. For each library, shown are theoretical ratios of the incorporated amino acids (excluding parental sequence contribution, when applicable). Only positions that were mutated at least once during the design and evolution process are shown. Blank space means that the wild-type amino acid was kept. Highlighted pink – mutations inspired by initial computational modeling, yellow – by results of the previous round(s), blue – by natural TetR family diversity (Supplementary Fig. 7), purple – by manual inspection of the docked models, orange – positions screened by site-saturation mutagenesis.


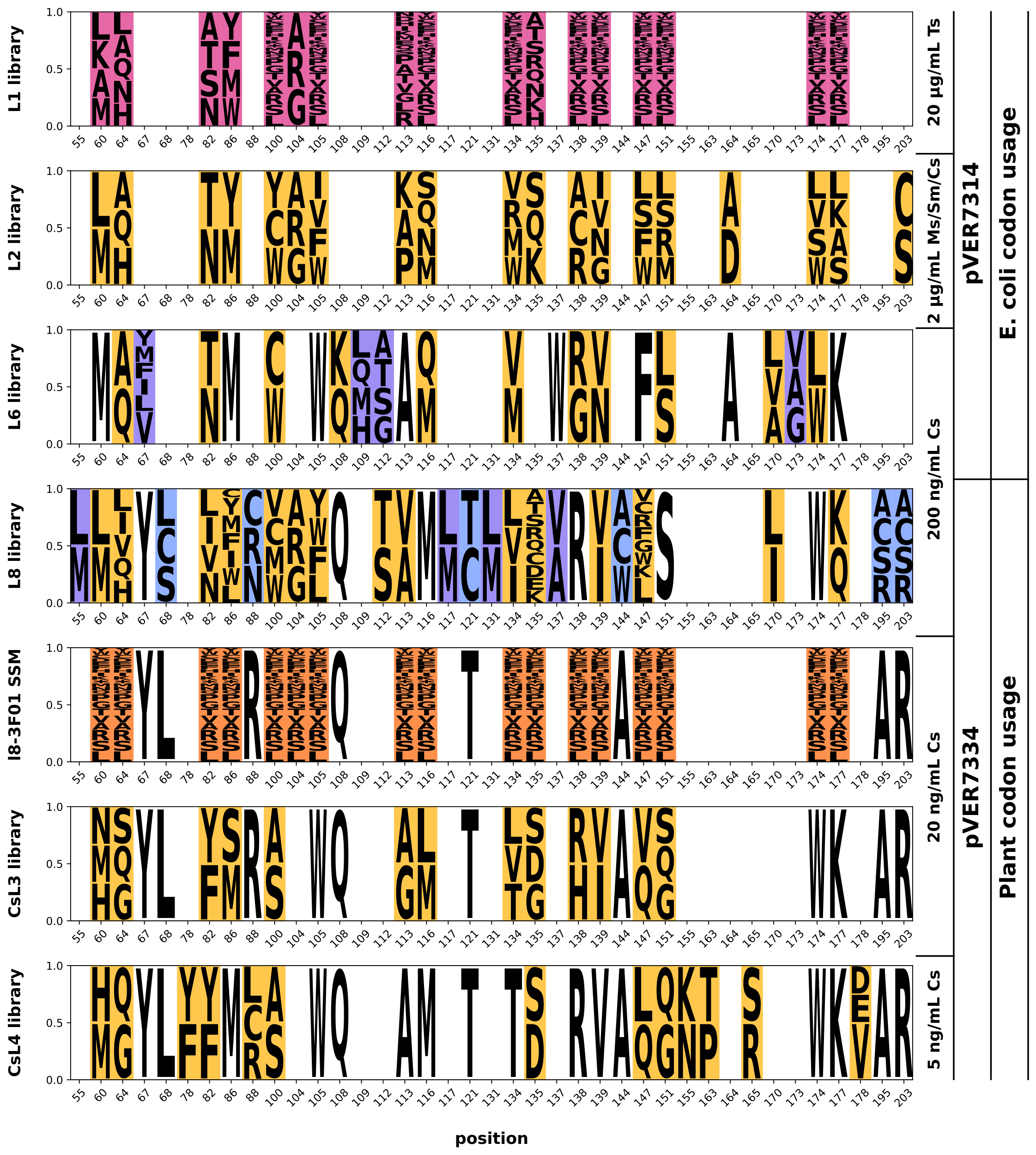


Supplementary Fig. 6. Summary of the design process of CsRs. For each library, shown are theoretical ratios of the incorporated amino acids (excluding parental sequence contribution, when applicable). Only positions that were mutated at least once during the design and evolution process are shown. Blank space means that the wild-type amino acid was kept. Highlighted pink – mutations inspired by initial computational modeling, yellow – by results of the previous round(s), blue – by natural TetR family diversity (Supplementary Fig. 7), purple – by manual inspection of the docked models, orange – positions screened by site-saturation mutagenesis.


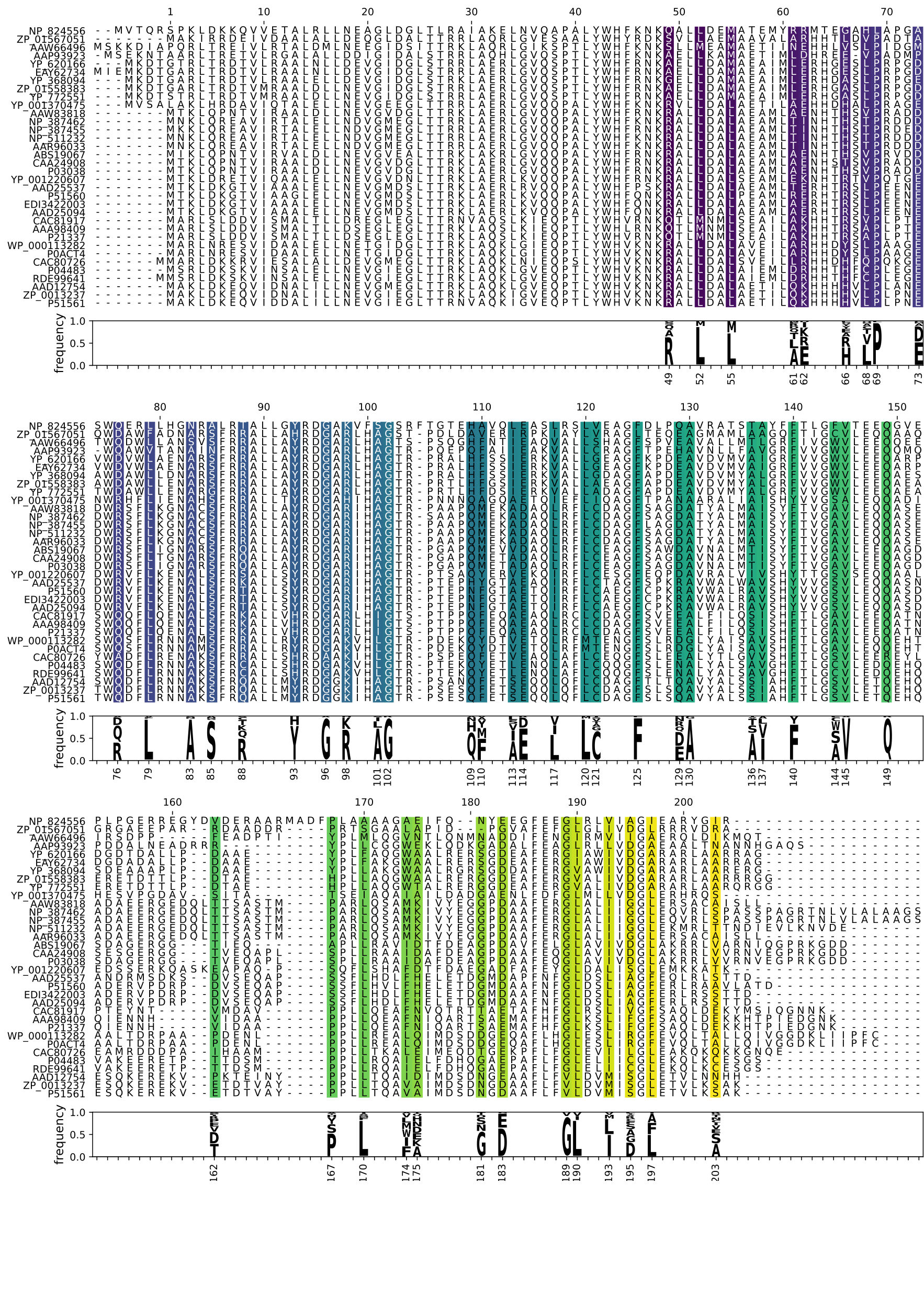


Supplementary Fig. 7. Alignment of 34 TetR homologs. 48 positions whose diversity was used to select amino acid mutations tested in the libraries are highlighted, and their amino acid frequencies are summarized below. Amino acid numbering is based on Class B TetR (P04483).


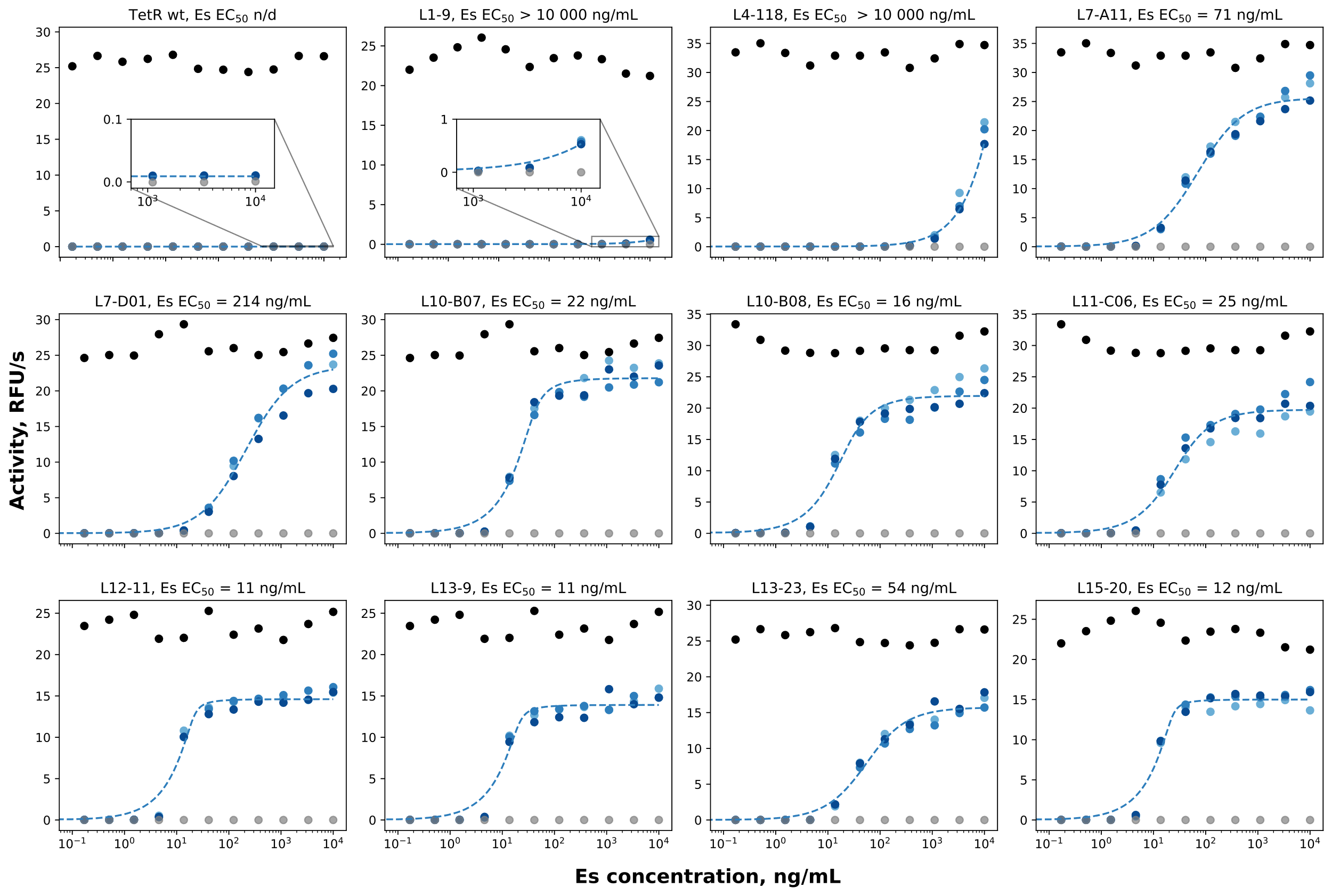


Supplementary Fig. 8. Derepression of selected EsRs by Es assessed using the β-galactosidase activity assay. Black - KM3 (positive control, constitutive β-galactosidase expression), gray - Top10 (negative control, no β-galactosidase expression), blue - three independent measurements of KM3 cells transformed with pVER7314 vector encoding wt TetR or one of its mutants. The dashed lines show the fitted curves used for determining EC_50_ values. Source data are provided as a Source Data file.


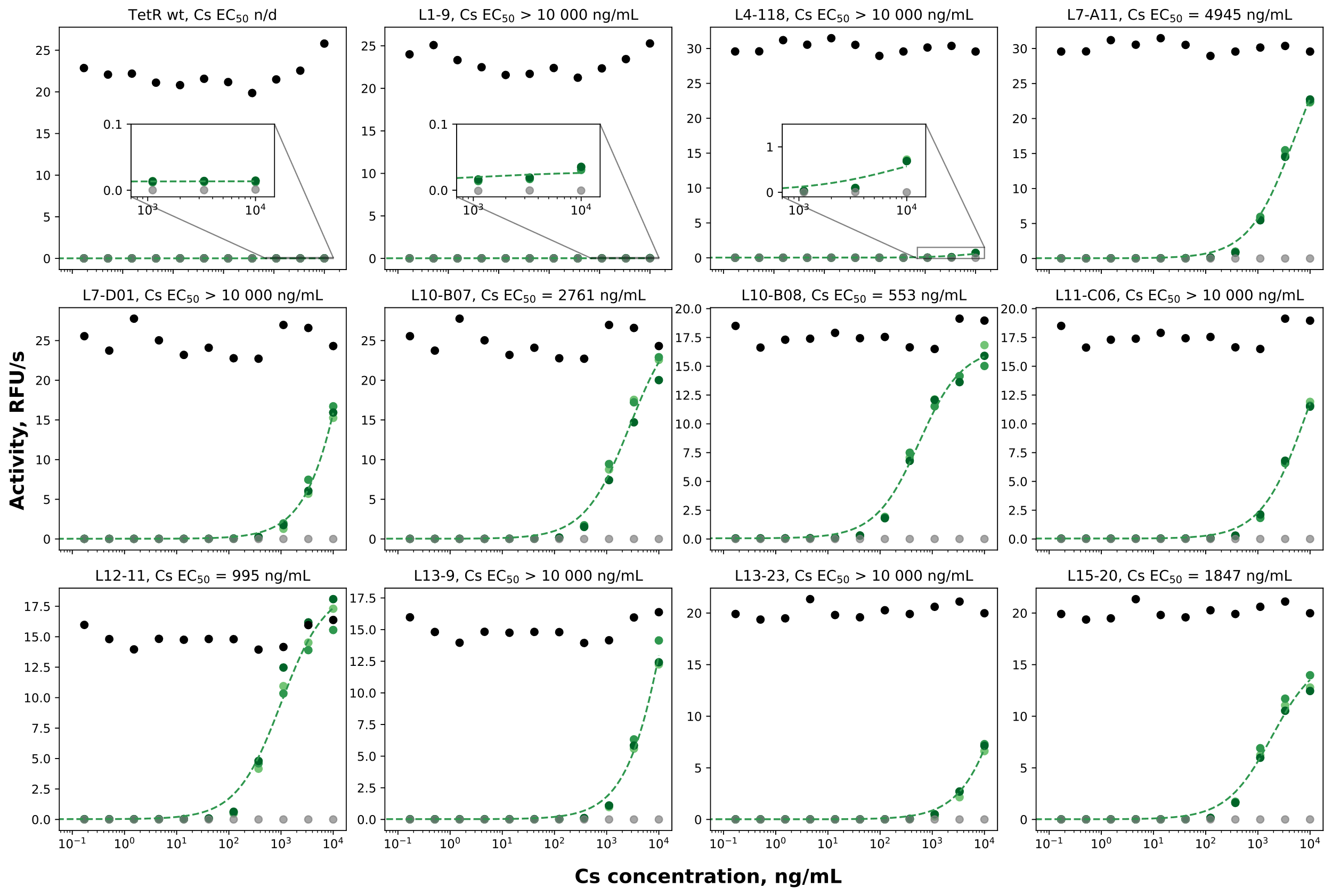


Supplementary Fig. 9. Derepression of selected EsRs by Cs assessed using the β-galactosidase activity assay. Black - KM3 (positive control, constitutive β-galactosidase expression), gray - Top10 (negative control, no β-galactosidase expression), green - three independent measurements of KM3 cells transformed with pVER7314 vector encoding wt TetR or one of its mutants. The dashed lines show the fitted curves used for determining EC_50_ values. Source data are provided as a Source Data file.

**
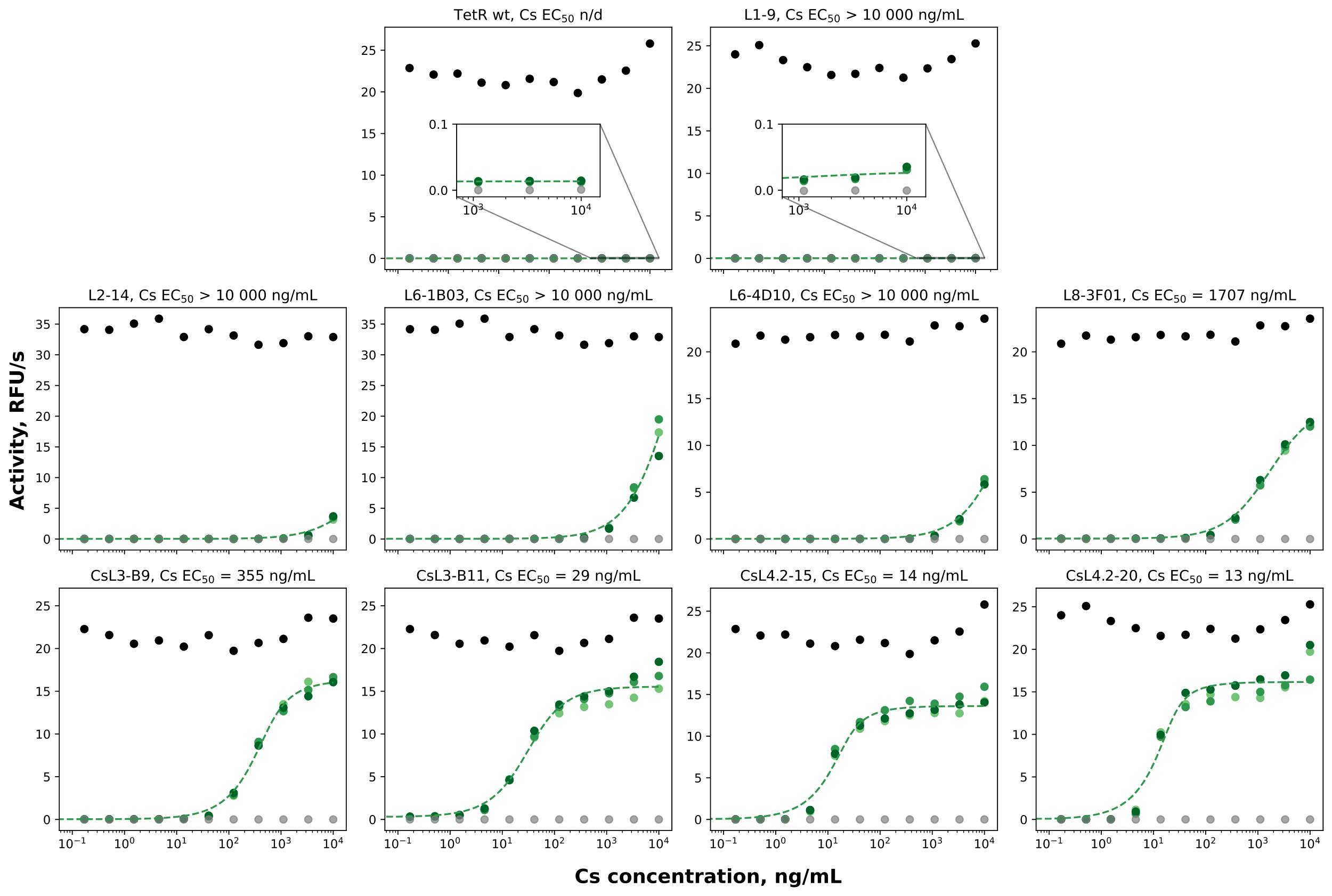
**

Supplementary Fig. 10. Derepression of selected CsRs by Cs assessed using the β-galactosidase activity assay. Black - KM3 (positive control, constitutive β-galactosidase expression), gray - Top10 (negative control, no β-galactosidase expression), green - three independent measurements of KM3 cells transformed with pVER7314 vector encoding wt TetR or one of its mutants. The dashed lines show the fitted curves used for determining EC_50_ values. Source data are provided as a Source Data file.


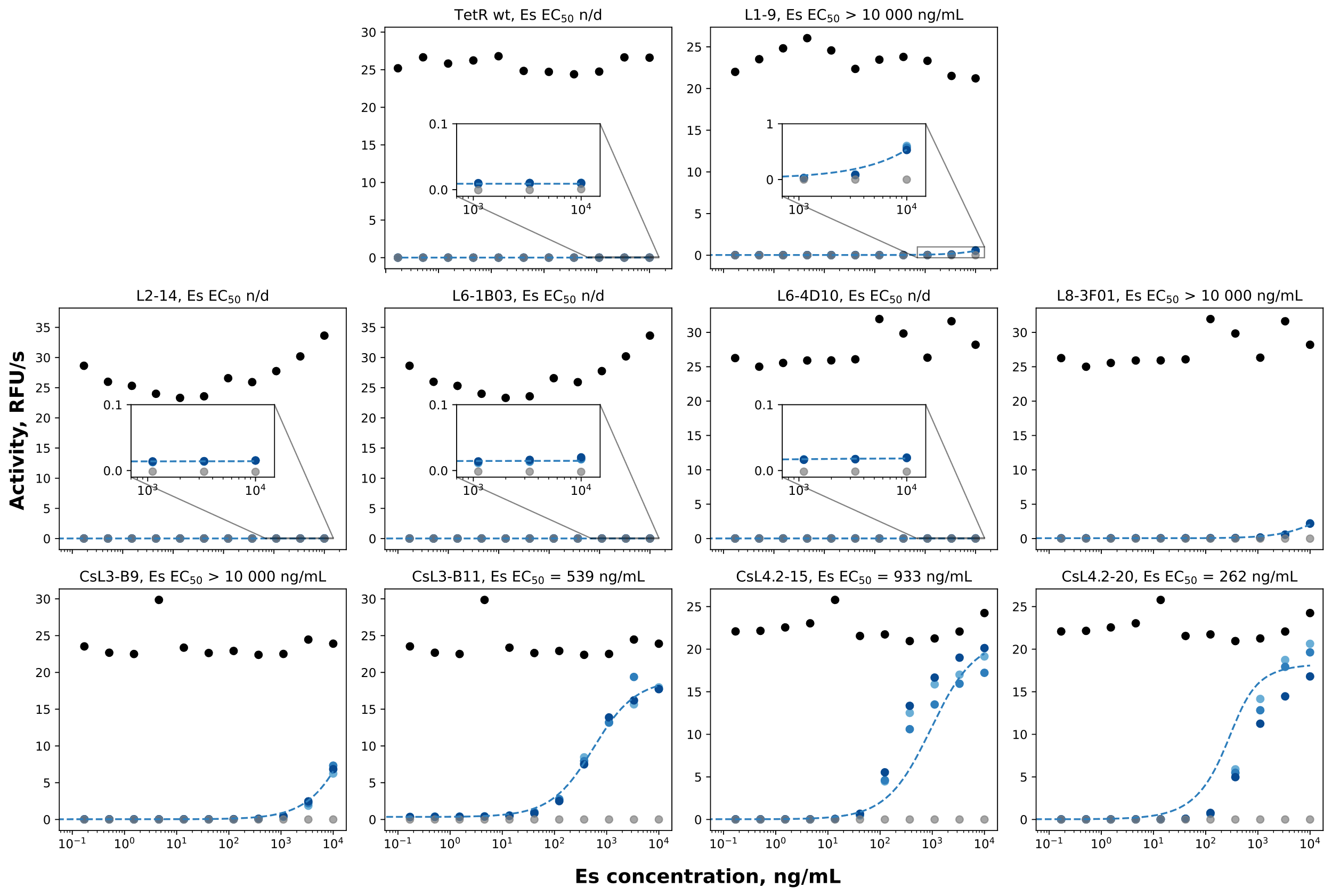


Supplementary Fig. 11. Derepression of selected CsRs by Es assessed using the β-galactosidase activity assay. Black - KM3 (positive control, constitutive β-galactosidase expression), gray - Top10 (negative control, no β-galactosidase expression), blue - three independent measurements of KM3 cells transformed with pVER7314 vector encoding wt TetR or one of its mutants. The dashed lines show the fitted curves used for determining EC_50_ values. Source data are provided as a Source Data file.


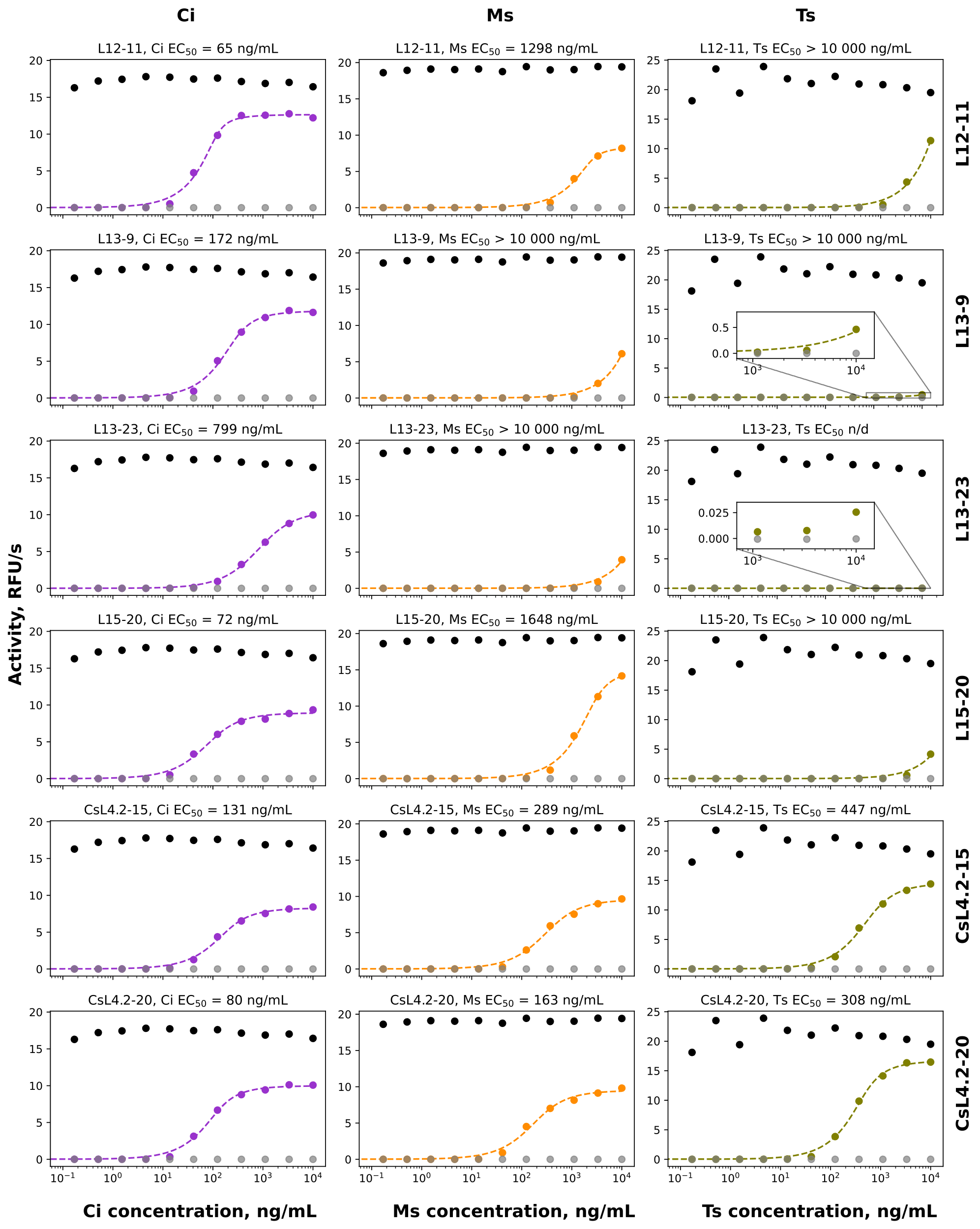


Supplementary Fig. 12. Derepression of selected EsRs and CsRs by Ci, Ms, or Ts assessed using the β-galactosidase activity assay. Black - KM3 (positive control, constitutive β-galactosidase expression), gray - Top10 (negative control, no β-galactosidase expression), colored - KM3 cells transformed with pVER7314 vector encoding one of the TetR mutants. The dashed lines show the fitted curves used for determining EC_50_ values. Source data are provided as a Source Data file.


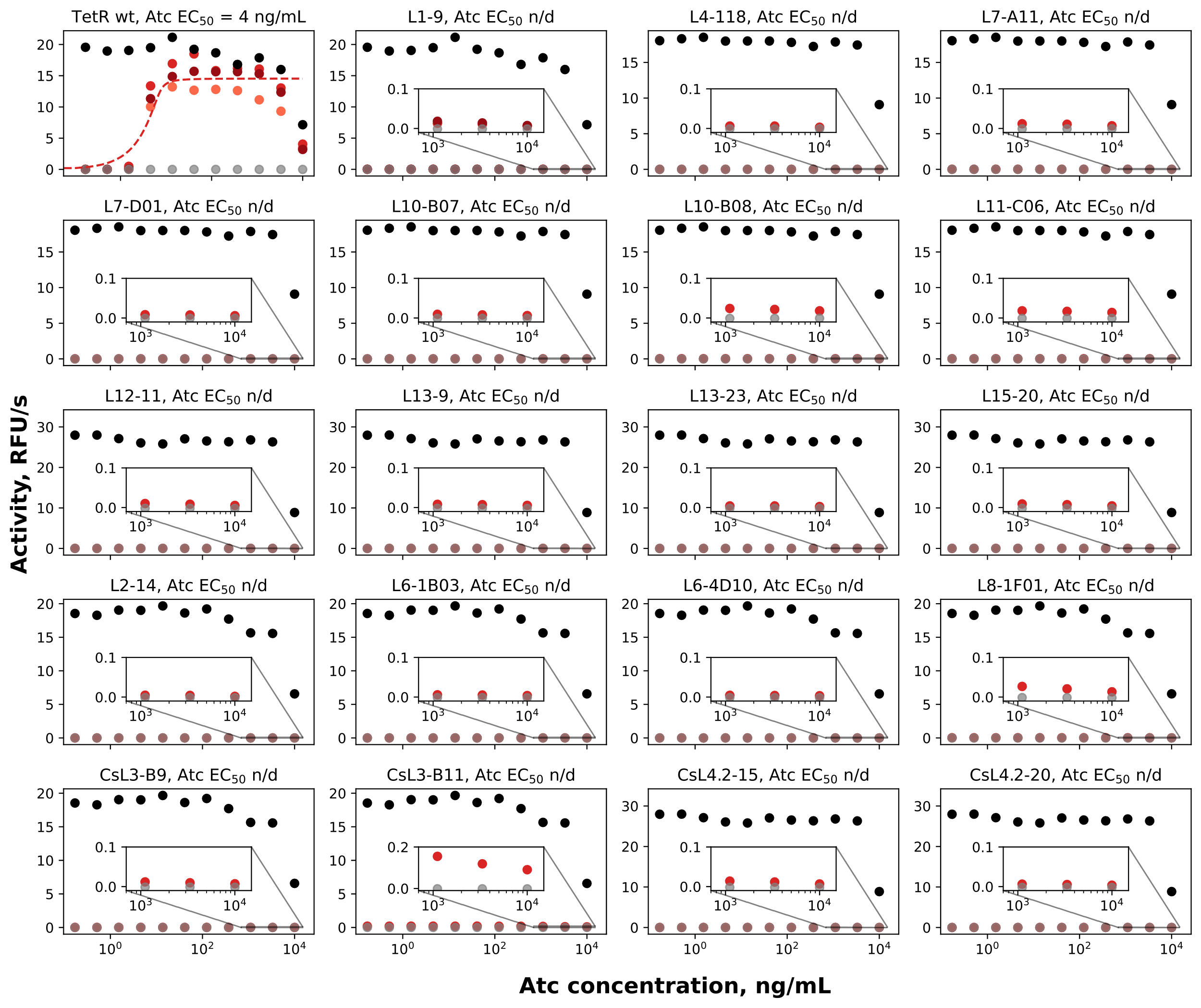


Supplementary Fig. 13. Derepression of selected EsRs and CsRs by Atc assessed using the β-galactosidase activity assay. Black - KM3 (positive control, constitutive β-galactosidase expression), gray - Top10 (negative control, no β-galactosidase expression), red - KM3 cells transformed with pVER7314 vector encoding wt TetR or one of its mutants. Three independent measurements were done only for wtTetR and L1-9. The dashed line shows the fitted curve used for determining EC_50_ values. The last two points (3,300 and 10,000 ng/mL) were not used for fitting as these concentrations of Atc seem to be toxic to the bacteria. Source data are provided as a Source Data file.


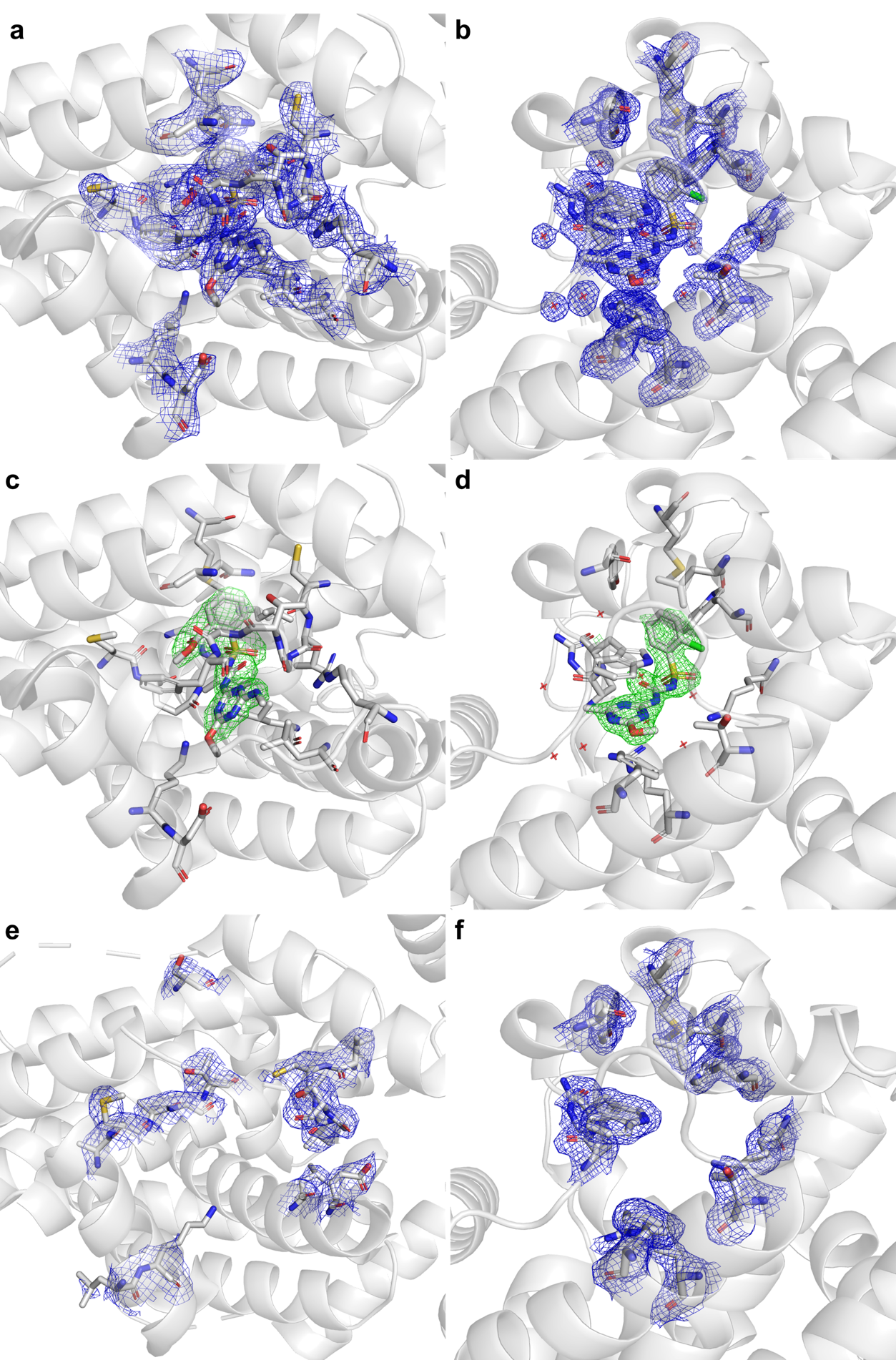


Supplementary Fig. 14. Representative electron density for the structures solved in this study. ****a,b**** 2F_o_–F_c_ electron density maps (blue, contoured at 1.0 σ) shown for the ligand and surrounding residues and water molecules within 4 Å of the ligand. ****c,d**** Ligand-omit F_o_–F_c_ difference maps (green, contoured at +3.0 σ) shown for the same regions as in ****a,b****. ****e,f**** 2F_o_–F_c_ maps (blue, contoured at 1.0 σ) shown for the corresponding regions in the apo structures. ****a,c**** Round four EsR hit L11-C06 in complex with Es. ****b,d**** Round six CsR hit CsL4.2-20 in complex with Cs. ****e**** Round three EsR hit L7-D01 in the apo form. ****f**** Round six CsR hit CsL4.2-20 in the apo form.


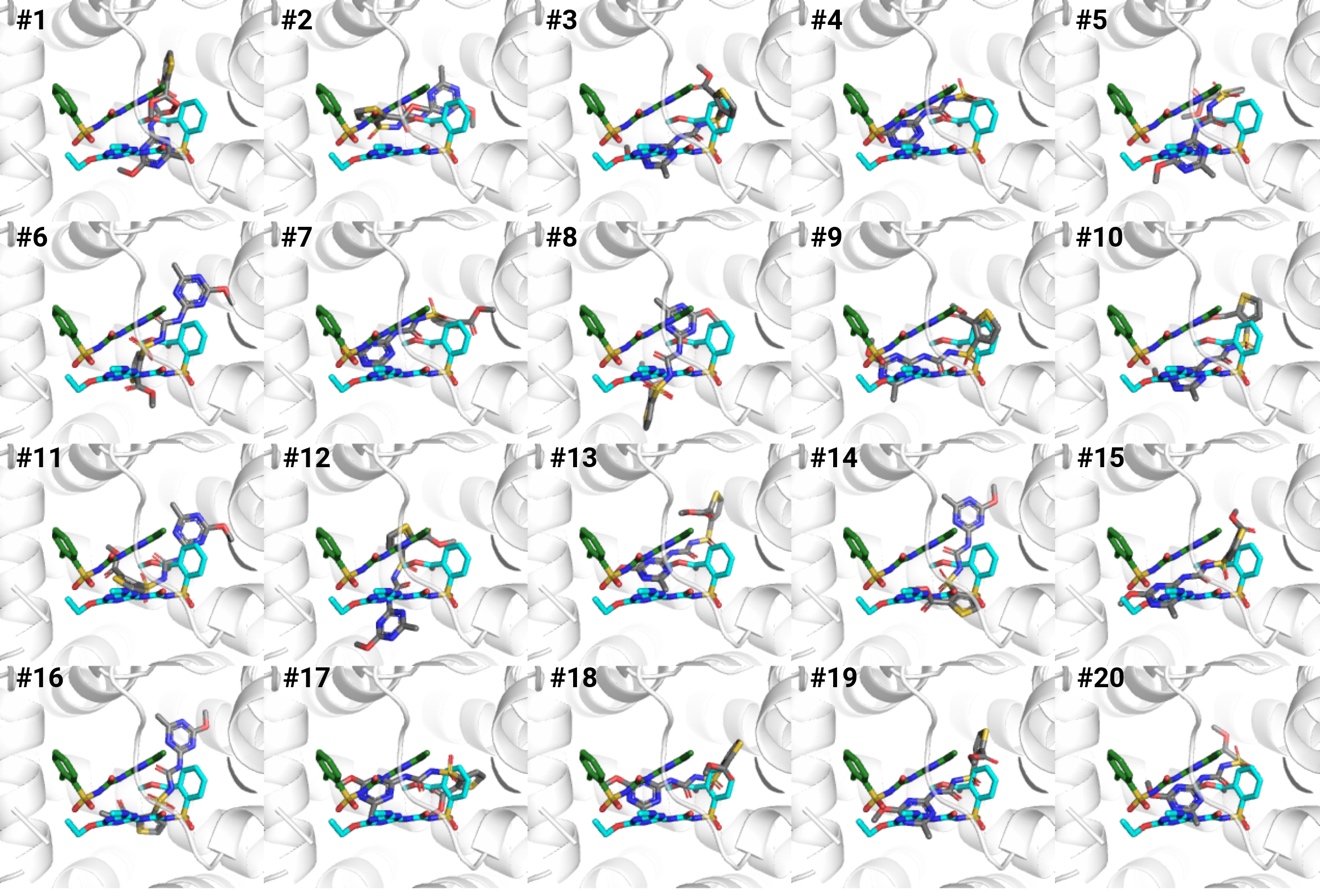


Supplementary Fig. 15. Position of the representative pose from each of 20 docked Ts clusters (gray) relative to the positions of Es and Cs (showed as cyan and green sticks, respectively) in the TetR binding pocket.


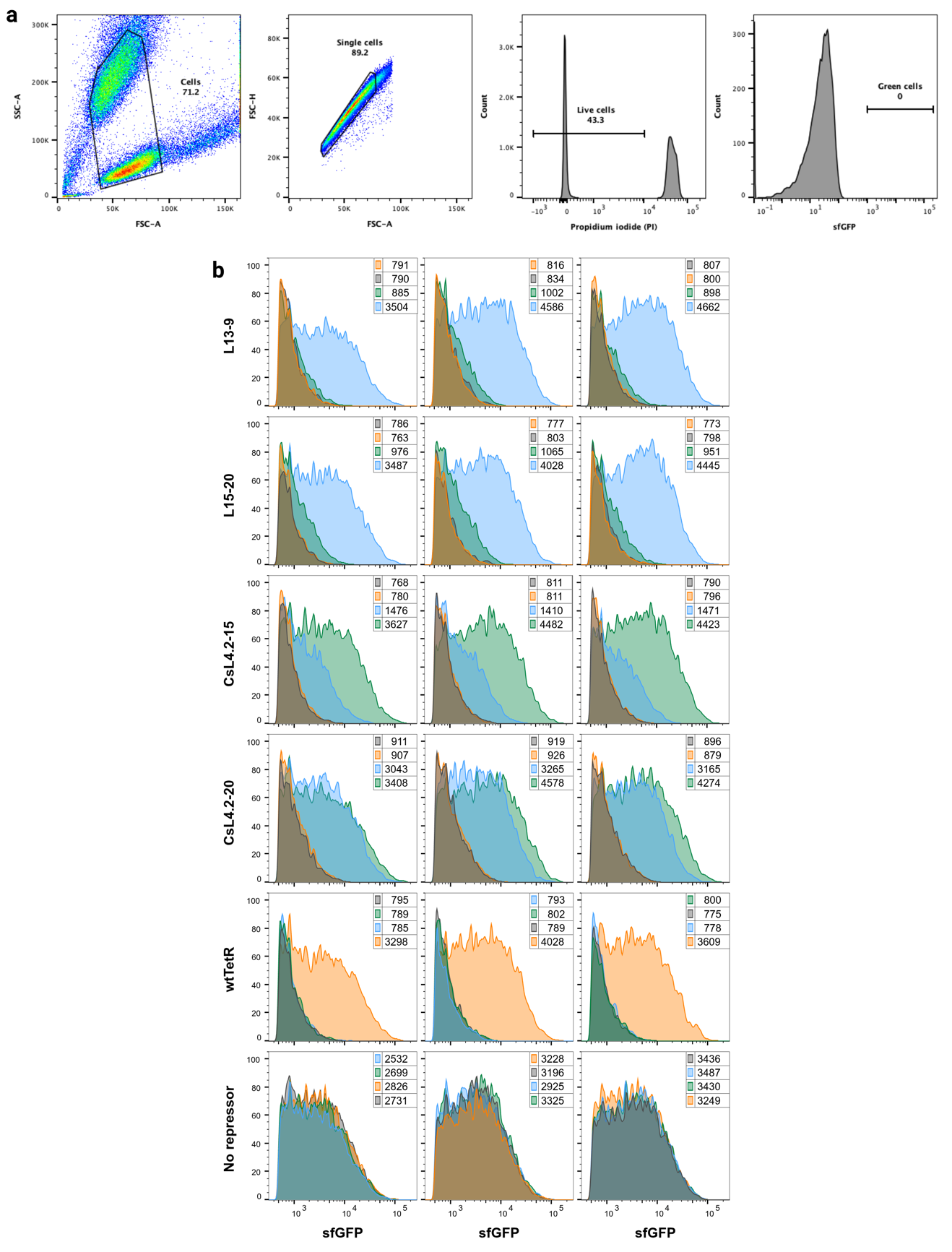


Supplementary Fig. 16. SUR function in mammalian cells. a Gating strategy for live, sfGFP-expressing cells. b Flow cytometry histogram plots showing the sfGFP fluorescence intensity of cells transiently co-transfected with the *TetO*-sfGFP reporter plasmid and one of the repressor-expressing plasmids or an empty vector 24 hours after the addition of 1 μg/mL Tc (orange), Es (blue), Cs (green), or DMSO (0.1%, carrier solution, gray). Shown are 3 independent experiments (columns). Numbers represent the median intensity.


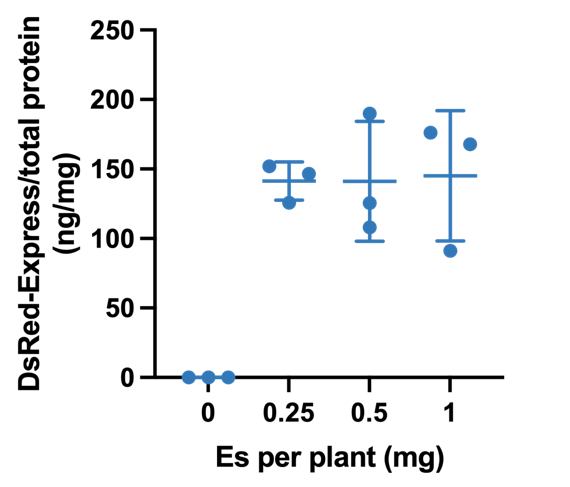


Supplementary Fig. 17. Induction of DsRed-Express in soy plants following root soak with Es. Soybean seedlings harboring construct PHP45473 were treated with 20 mL Muster^®^ dilutions containing 0.25, 0.5, or 1 mg of Es active ingredient or water. DsRed-Express protein expression was quantified 6 days post-treatment. *n*=3, shown are mean and std. dev. Source data are provided as a Source Data file.


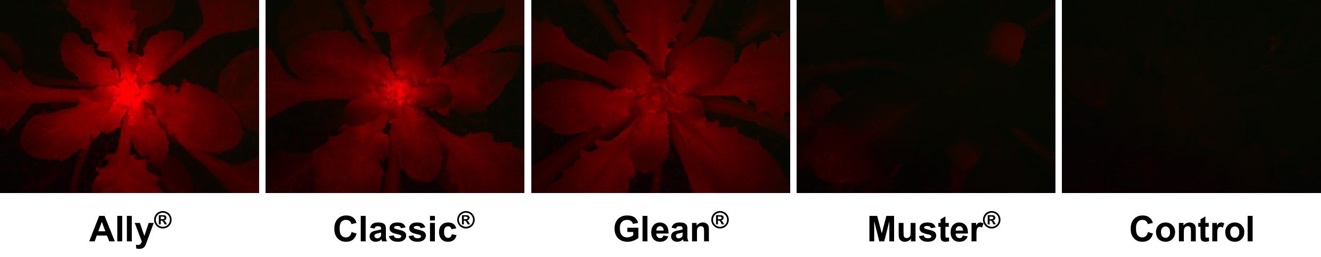


Supplementary Fig. 18. Induction of DsRed-Express in *Arabidopsis* following spray application of different SU herbicides. T2 seedlings of inducible *A. thaliana* transformants harboring construct pVER7384 were sprayed at the rosette stage with Ally^®^ (Ms), Classic^®^ (Ci), Glean^®^ (Cs), or Muster^®^ (Es) at the 0.25x application rate (50 mg/L). A null segregant treated with Classic^®^ was used as a control. Plants were imaged 3 days after SU application using a Leica M165 fluorescent stereoscope fitted with a DsRed filter set and CCD camera. All photographs were taken with identical exposure times and camera settings. Shown are representative images of three independent experiments.


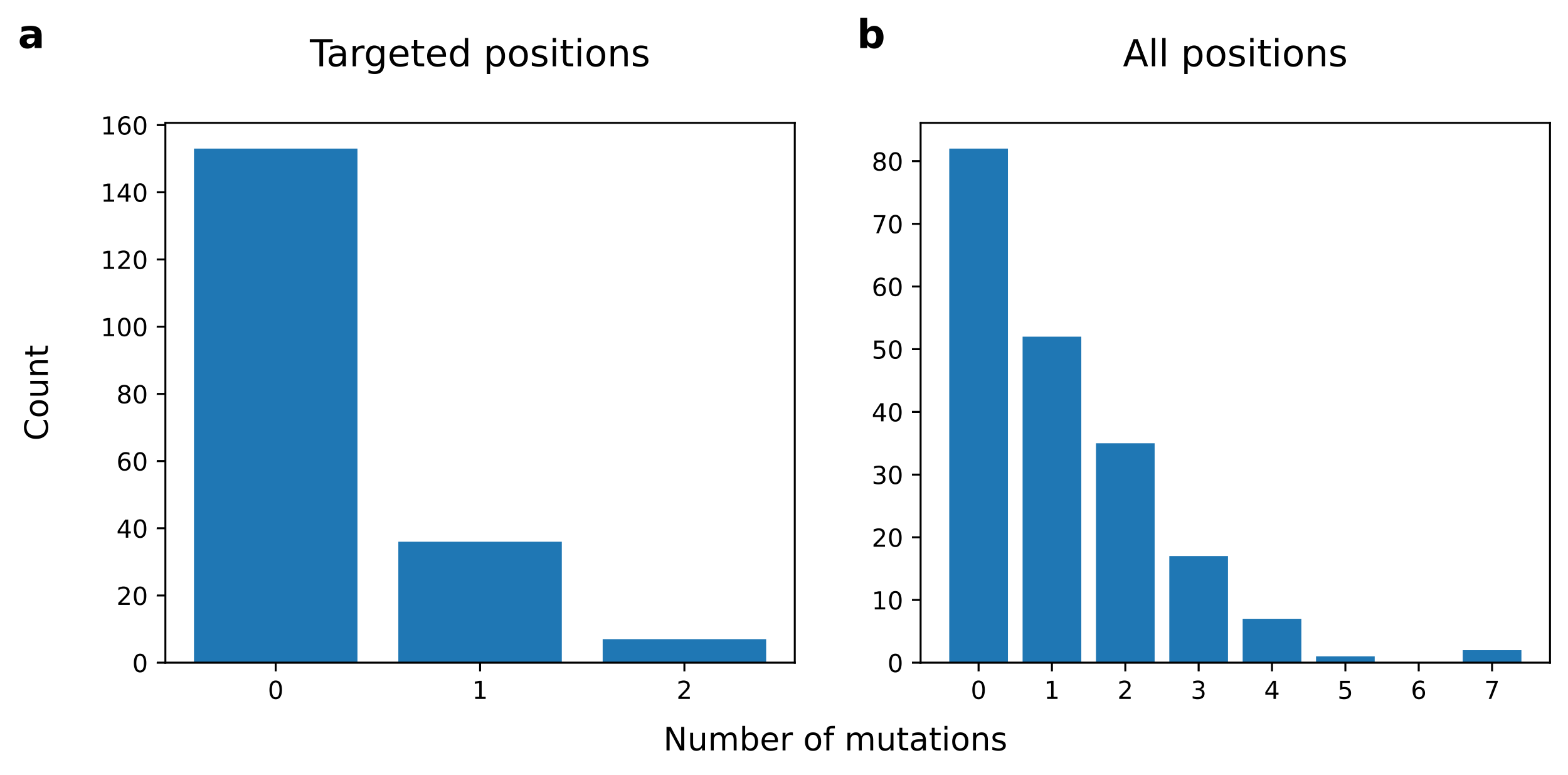


Supplementary Fig. 19. Incorporation of amino acid mutations into parental clone L1-9 at targeted (a) and all (b) positions in the LBD.

**
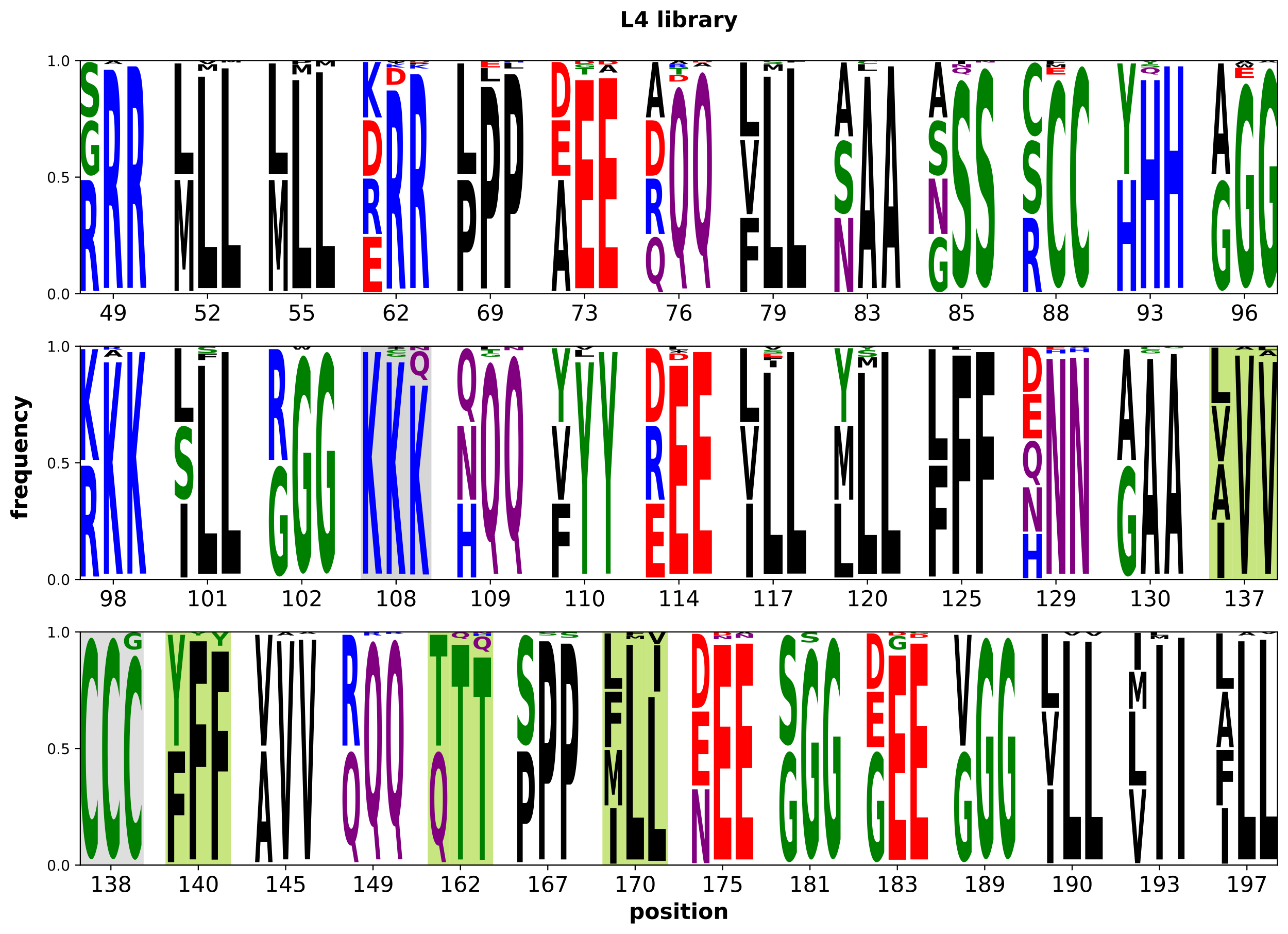
**

Supplementary Fig. 20. Round 2 library design, construction, and screening results. Shown are theoretical primer-encoded amino acid frequencies (column 1), and experimental frequencies of random colonies (10% of the diversity oligos, column 2) and hits (column 3). Positions with noticeable enrichment in hits at targeted positions (137, 140, 162, and 170) have a light green background; those that occurred randomly (108 and 138) have a light gray background.


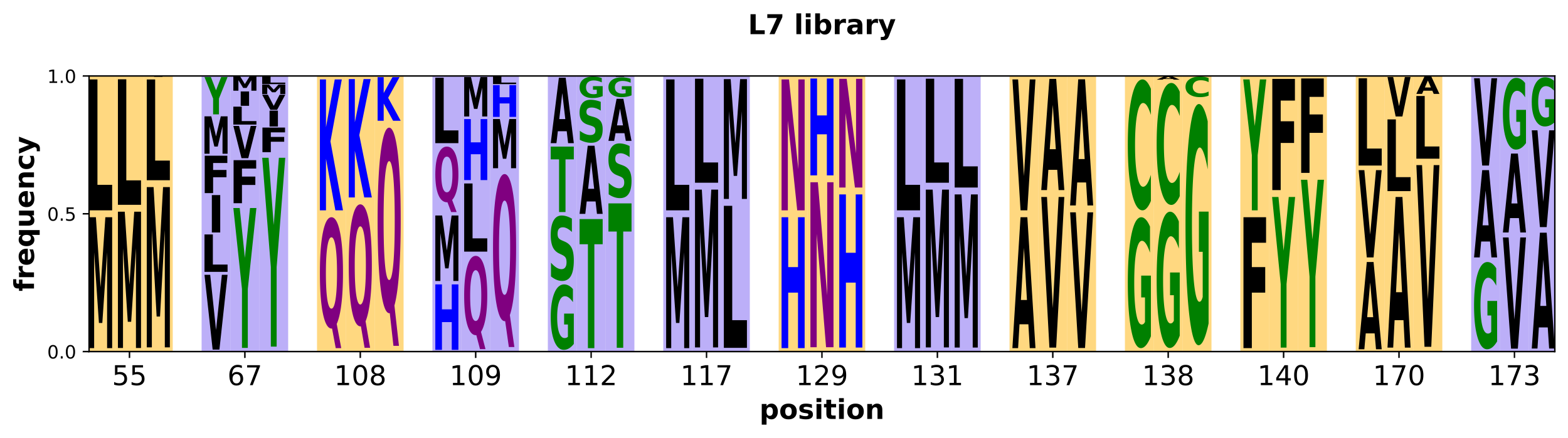


Supplementary Fig. 21. Round 3 library design, construction, and screening results. For each of the 13 positions selected for mutation, shown are the theoretical amino acid frequencies of the encoded library (column 1), and experimentally observed frequencies of random colonies (column 2) and hits (column 3). Positions at which the encoded diversity was inspired by L4 library results (55, 108, 129, 137, 138, 140, and 170) have a yellow background; those inspired by model inspection (67, 109, 112, 117, 131, and 173) have a purple background.


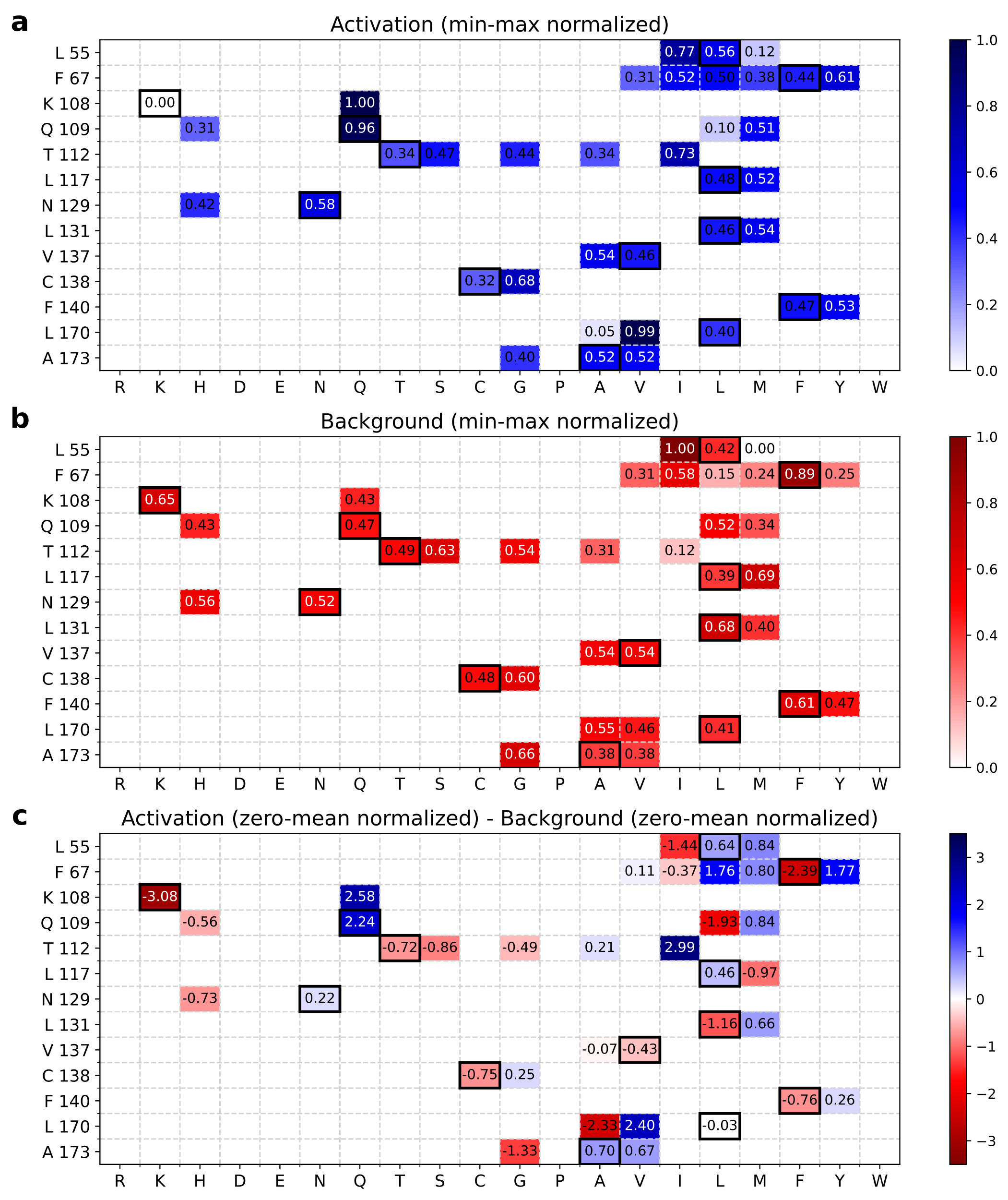
Supplementary Fig. 22. Apparent contribution of individual L7 library mutations to activation by Es (a), leakiness (b), and overall performance (c) based on regression analysis. The y-axis shows positions and L1-9 sequence. Columns represent the 20 canonical amino acids. Numbers in cells represent the normalized calculated contribution of having that amino acid in the specified position of L1-9 on the β-gal activity plate assay score. Mutations with predicted favorable contribution (increased activation and overall fitness, and decreased leakiness) are colored darker blue, mutations with unfavorable contribution (decreased activation and overall fitness, and increased leakiness) are colored darker red. L1-9 amino acids are outlined in black.


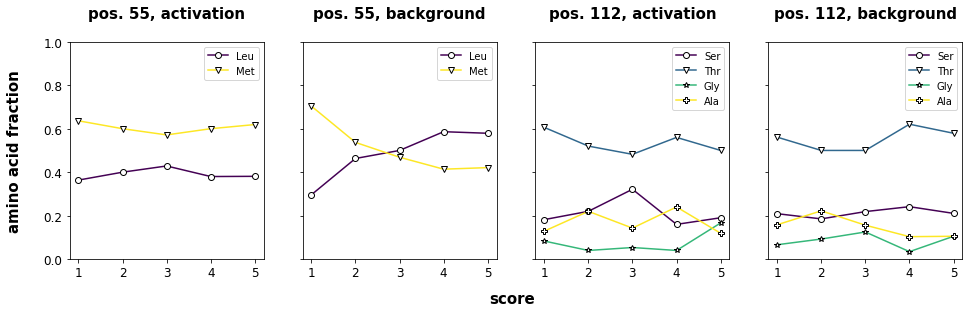


Supplementary Fig. 23. Observed fraction of amino acids in each β-gal activity score bin at positions 55 and 112. Only amino acids seen in >1% of hits are shown. Source data are provided as a Source Data file.


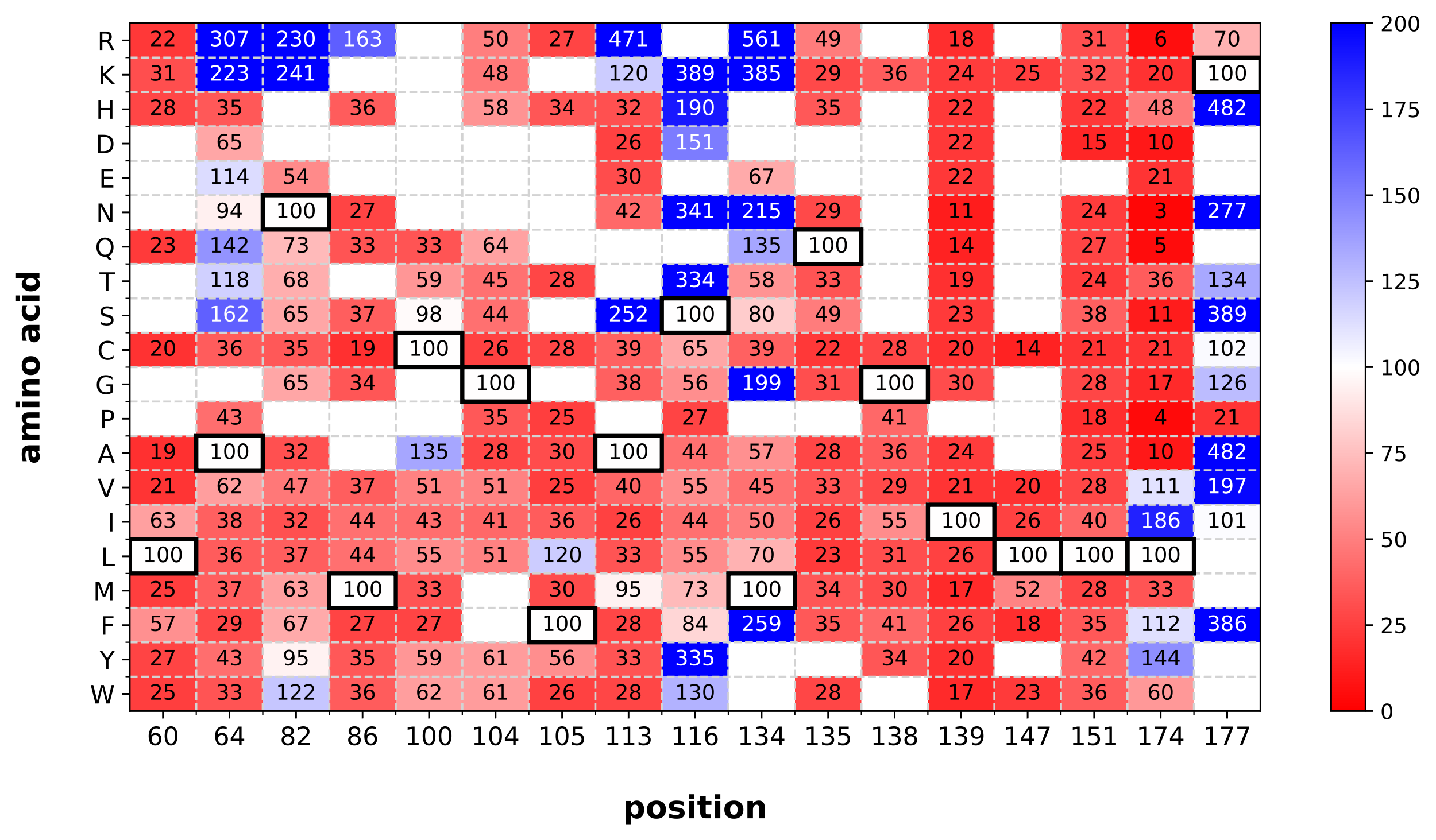


Supplementary Fig. 24. Site-saturation mutagenesis of L7-A11. Rows represent the 20 amino acids. Columns represent the 17 tested positions. Cells show response to 5 ng/mL Es as a percent of L7-A11 response. Mutations increasing activation are colored blue; those decreasing it are colored red. L7-A11 amino acids are outlined in black. Mutations increasing leakiness were not characterized for activation and are shown as blank cells.


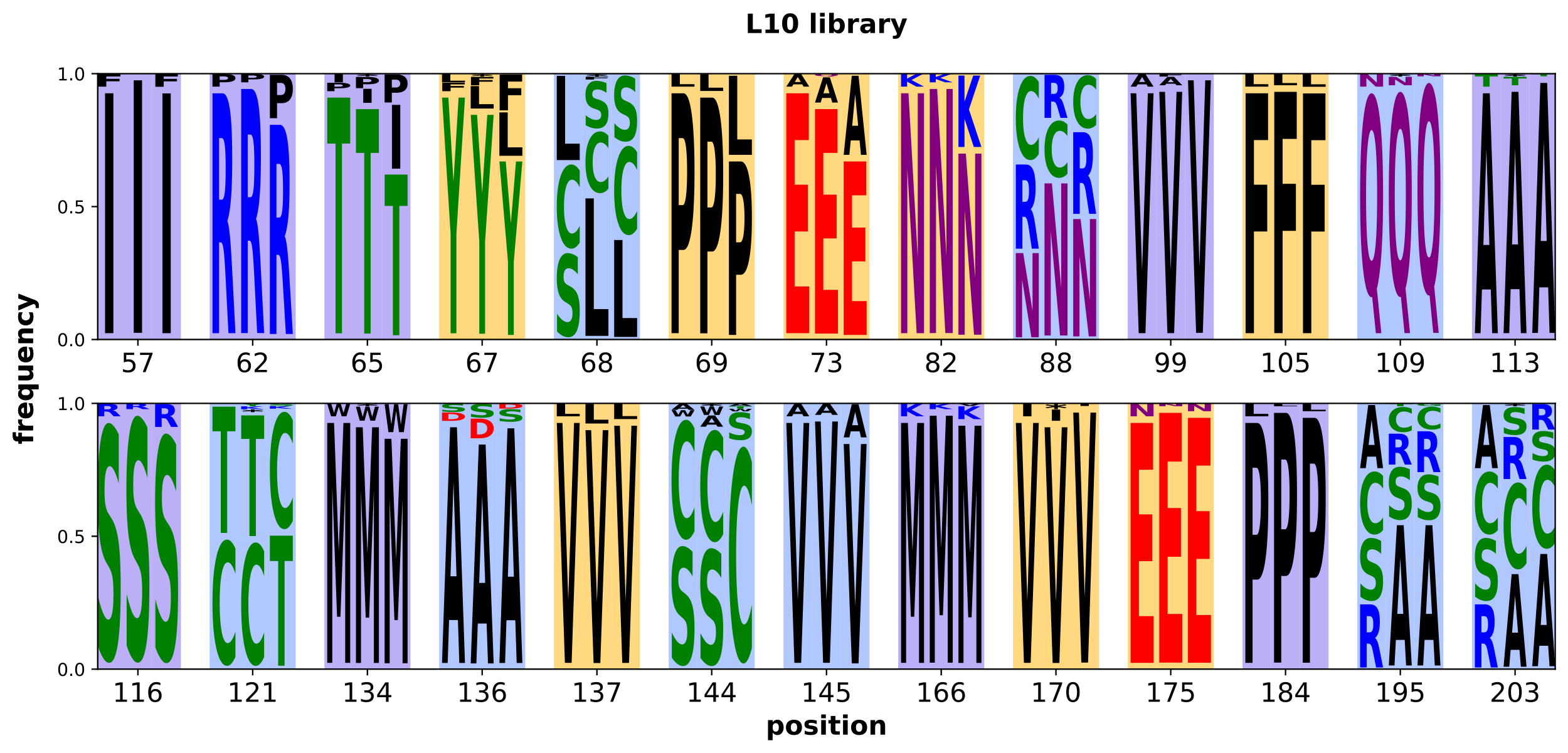


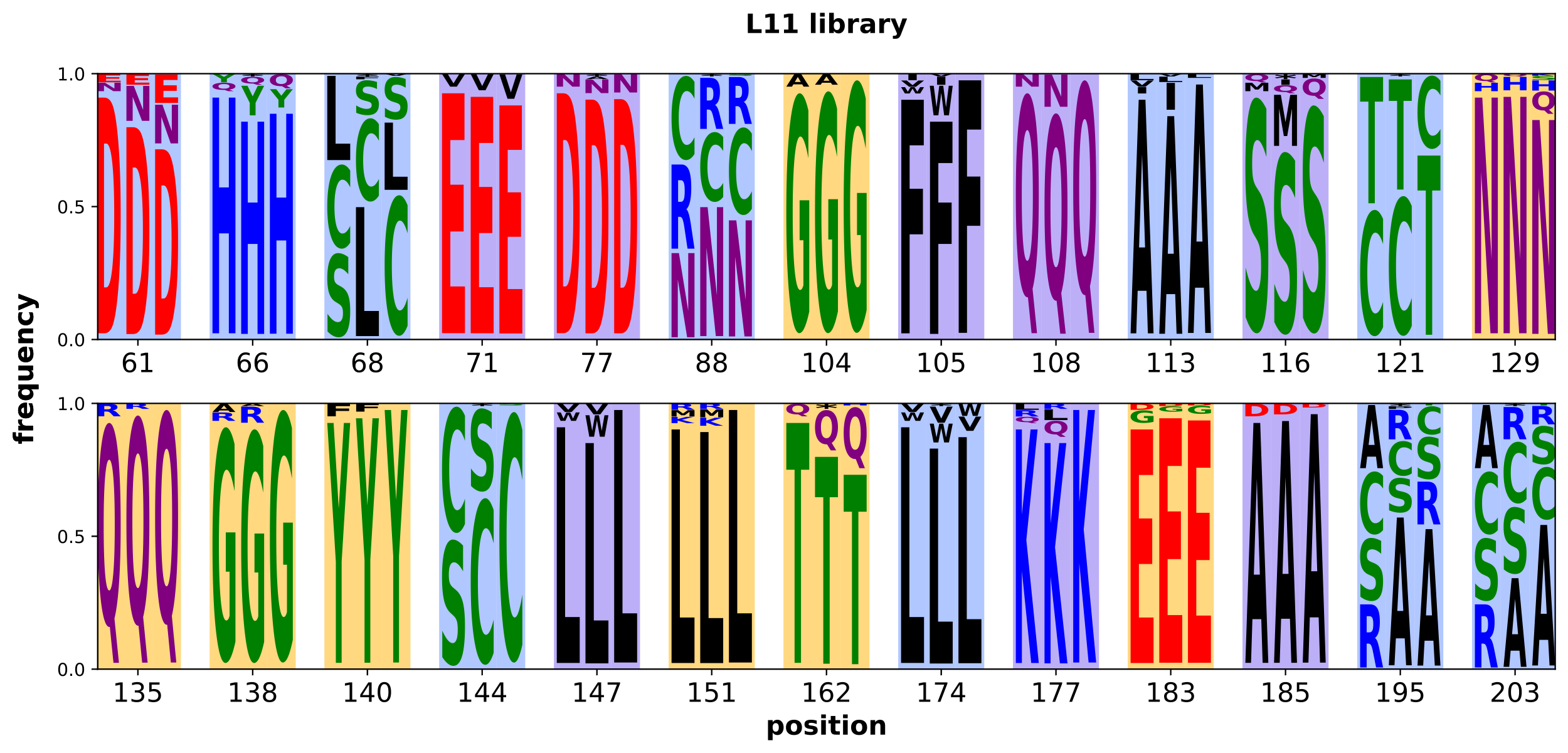


Supplementary Fig. 25. Libraries L10 and L11 design, construction, and screening results. (Top) Library L10 summary. (Bottom) Library L11 summary. At each of the 26 mutated positions, shown are the theoretical amino acid frequencies of the library containing 10% of the diversity oligonucleotides (column 1), and observed frequencies of the amino acids of random colonies (column 2) and selected hits (column 3). Positions in which the tested diversity was inspired by previous library screening results have a yellow background, by docked model inspection - a purple background, and by TetR family phylogenetic information - a blue background.


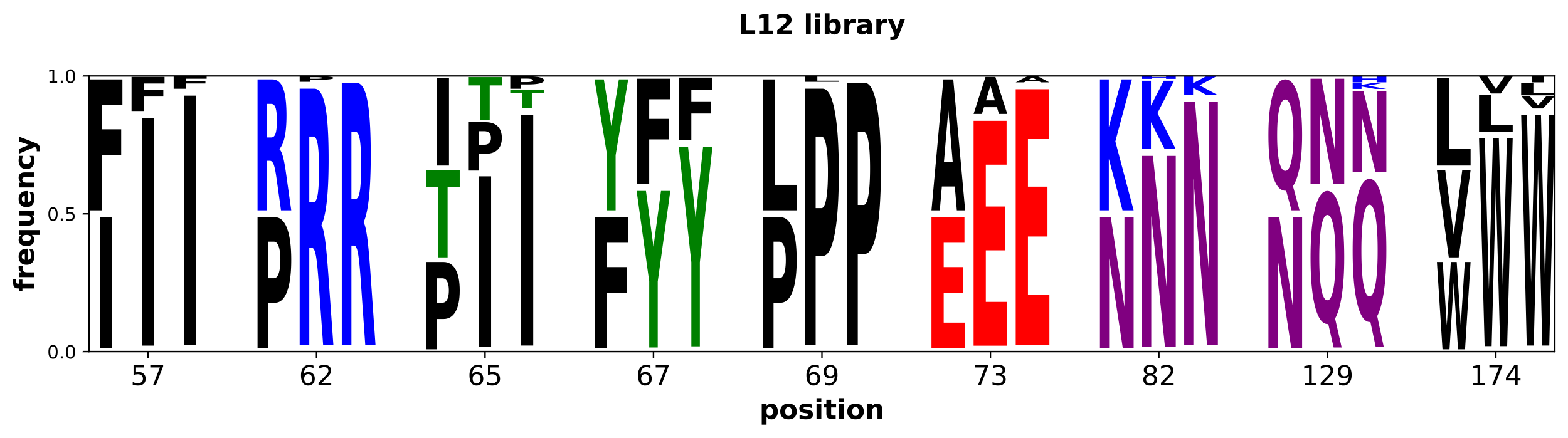


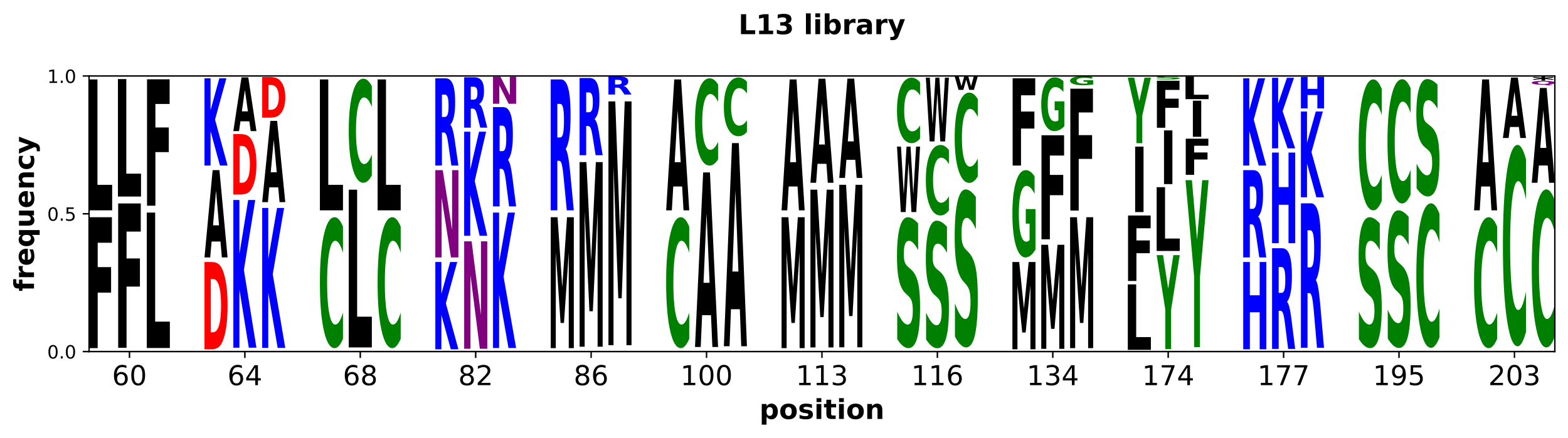


Supplementary Fig. 26. Round 5 library design, construction, and screening results. (Top) Library L12 summary. (Bottom) Library L13 summary. At each of the mutated positions, shown are the theoretical amino acid frequencies of the encoded library (column 1), and experimental frequencies of random clones (column 2) and selected hits (column 3).


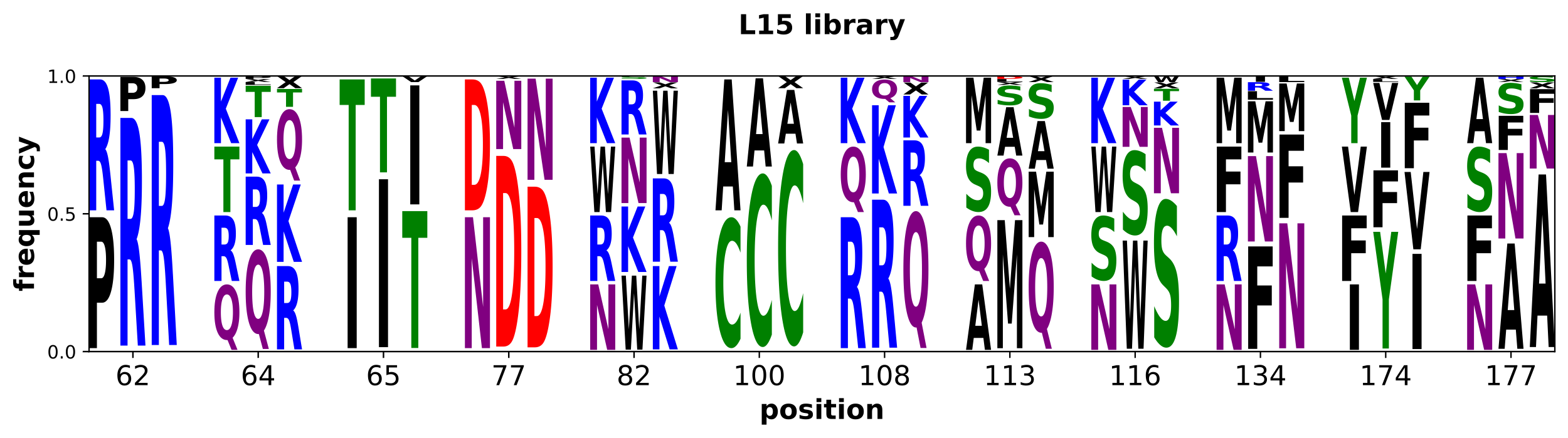


Supplementary Fig. 27. Round 6 library design, construction, and screening results. For each of the 12 positions selected for being mutated, shown are the theoretical amino acid frequencies of the encoded library (column 1), and experimental frequencies of the randomly picked colonies (column 2) and selected hits (column 3).


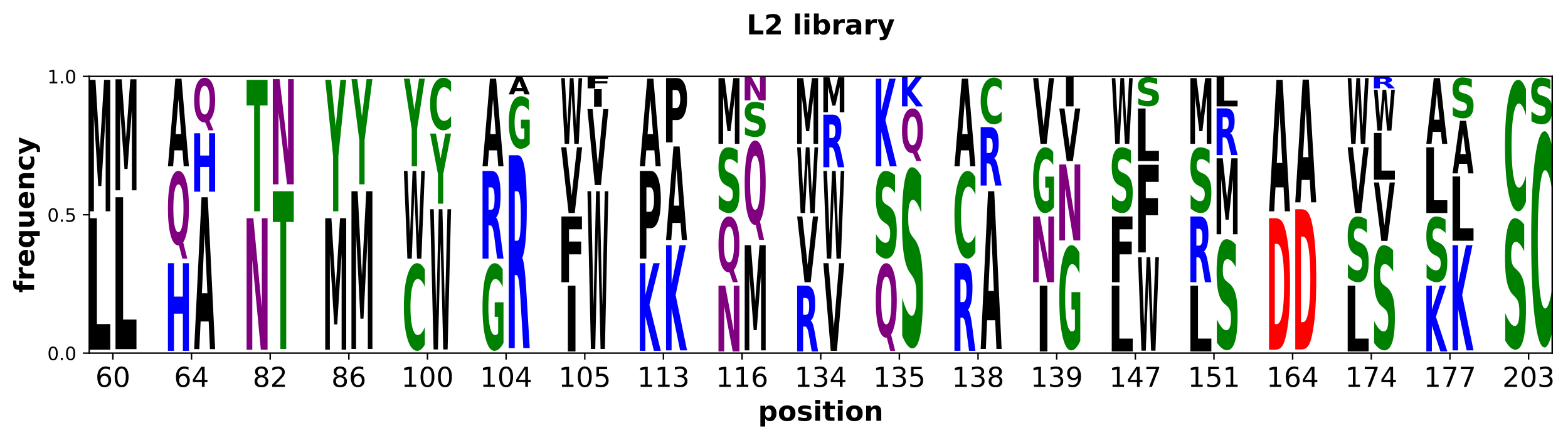


Supplementary Fig. 28. L2 library design and construction. For each of the 19 positions mutated at least in one of the four parental 1^st^ round library clones, shown are the theoretical amino acid frequencies of the encoded library (column 1), and experimental frequencies of random colonies (column 2).


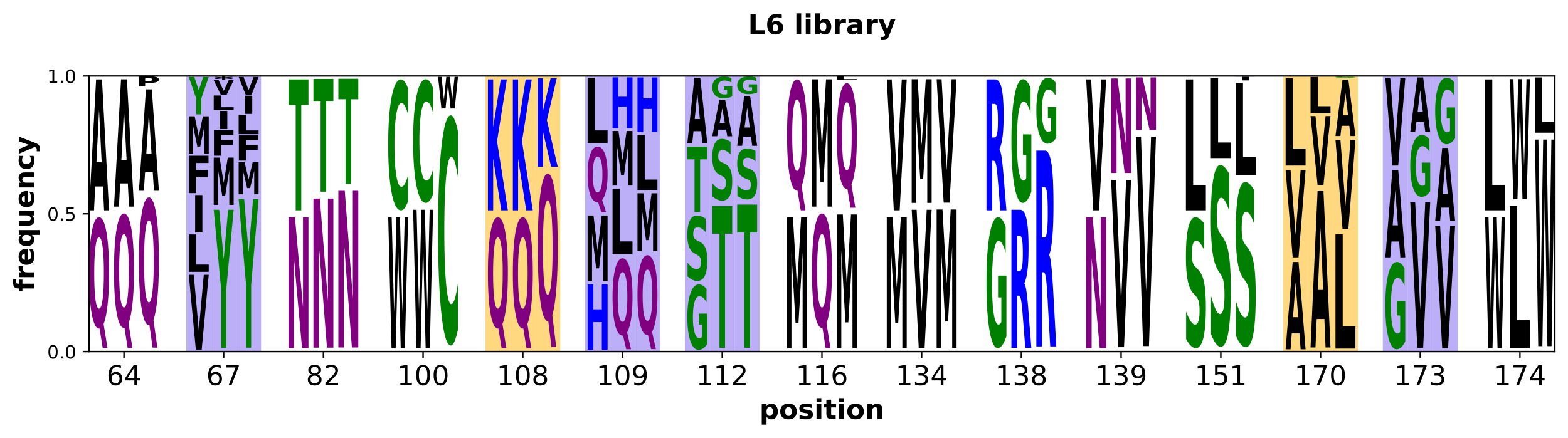


Supplementary Fig. 29. Round 3 library design, construction, and screening results. For each of the 15 positions mutated, shown are the theoretical amino acid frequencies of the encoded library (column 1), and experimental frequencies of random colonies (column 2) and selected hits (column 3). Positions in which the tested diversity was inspired by L4 library results have a yellow background, L7 library - a purple background, and by L2-14 and L2-18 hits - a white background.


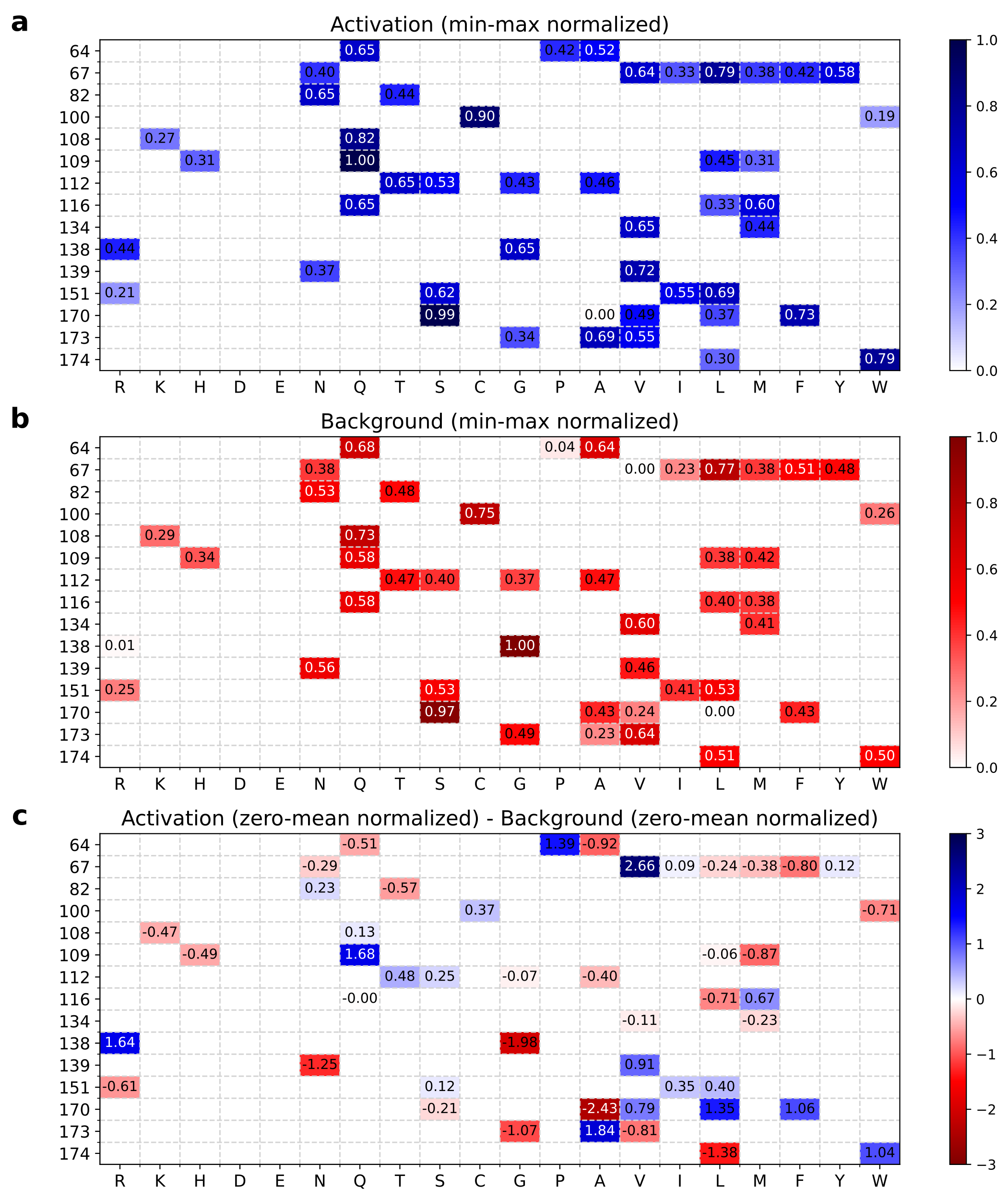


Supplementary Fig. 30. Apparent contribution of individual L6 library mutations to activation by Cs (a), leakiness (b), and overall performance (c) based on regression analysis. The y-axis shows amino acid positions. Columns represent the 20 canonical amino acids. Numbers in cells represent the normalized calculated contribution of having that amino acid in the specified position on the β-gal activity plate assay score. Mutations with predicted favorable contribution (increased activation and overall fitness, and decreased leakiness) are highlighted in darker blue, mutations with unfavorable contribution (decreased activation and overall fitness, and increased leakiness) are highlighted in darker red. Data used: leakiness after ~84 hours of incubation at 30 °C, activation after ~48 hours at 30 °C with 0.2 μg/mL Cs.


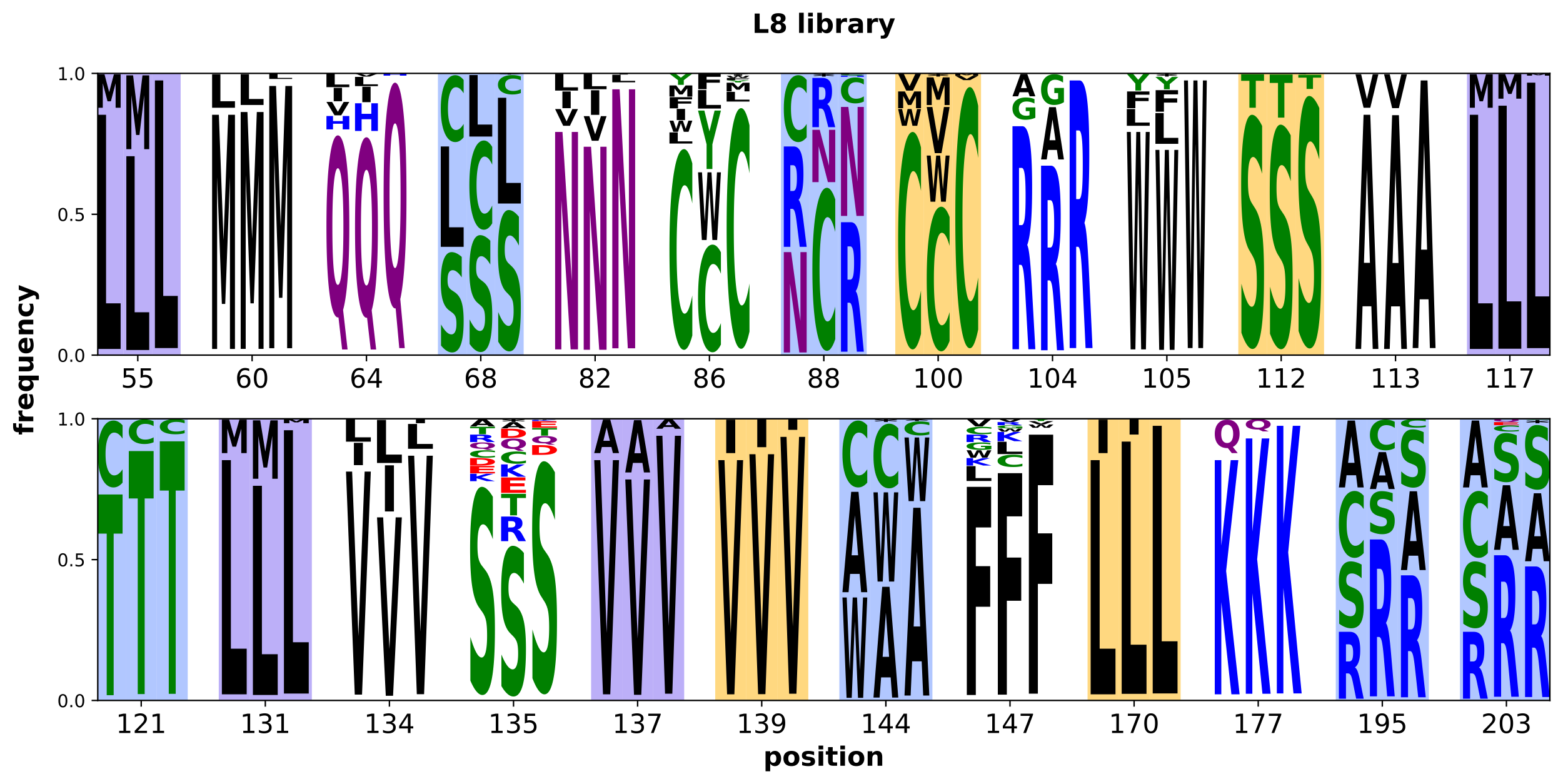


Supplementary Fig. 31. Library L8 design, construction, and screening results. For each of the 25 positions selected to mutate, shown are the theoretical amino acid frequencies of the library containing 25% of the diversity oligonucleotides (column 1), and observed frequencies of the amino acids in randomly picked colonies (column 2) and selected hits (column 3). Positions in which the tested diversity was inspired by L6 library results have a yellow background, by L7 library design - a purple background, by TetR family phylogenetic information (cysteine residues) - a blue background, and by initial binding pocket residues screening – a white background.


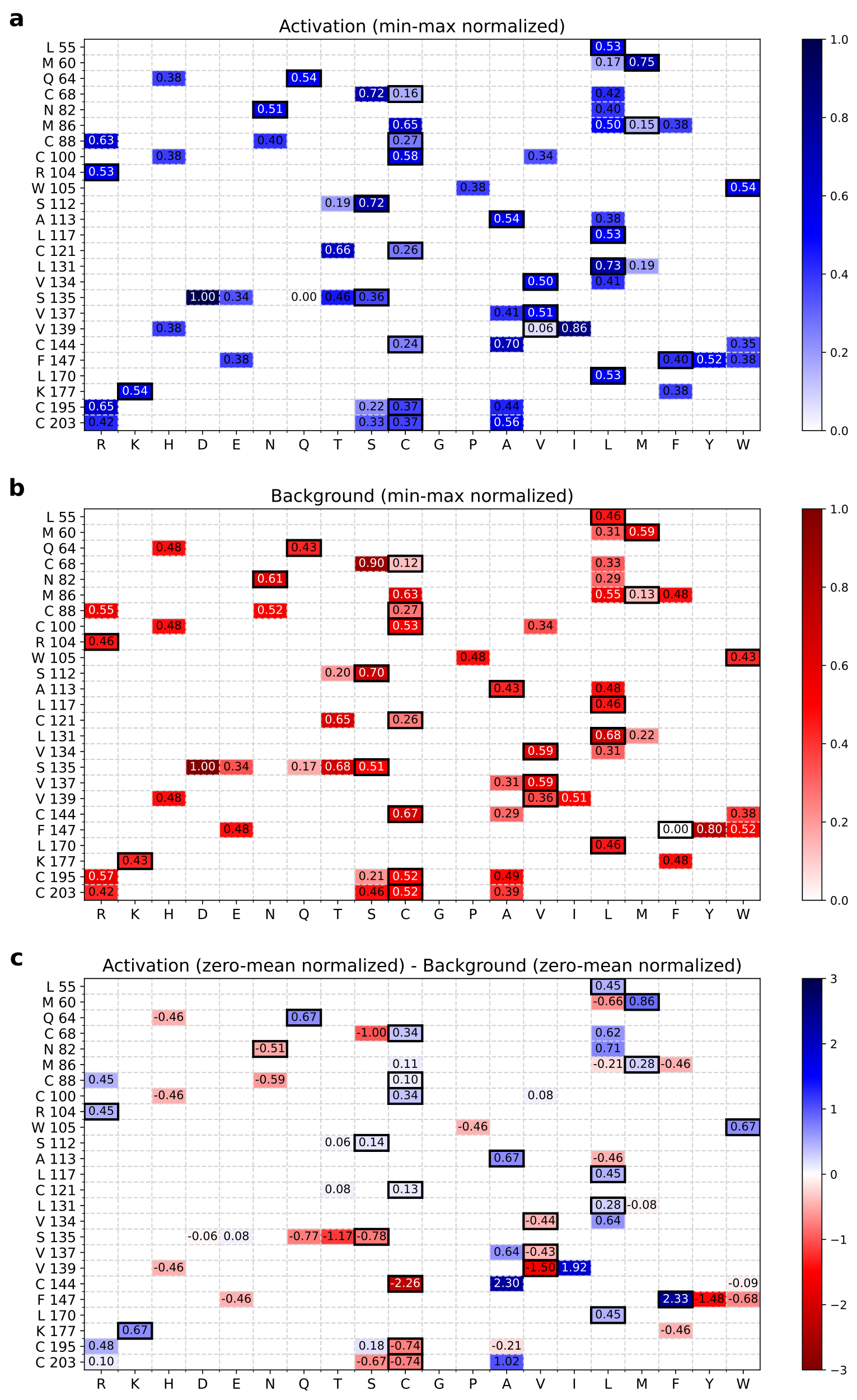


Supplementary Fig. 32. Apparent contribution of individual L8 library mutations to activation by Cs (a), leakiness (b), and overall performance (c) based on regression analysis. The y-axis shows positions and L6-4D10 sequence. Columns represent the 20 canonical amino acids. Numbers in cells represent the normalized calculated contribution of having that amino acid in the specified position on the β-gal activity plate assay score. Mutations with predicted favorable contribution (increased activation and overall fitness, and decreased leakiness) are highlighted in darker blue, mutations with unfavorable contribution (decreased activation and overall fitness, and increased leakiness) are highlighted in darker red. Original L6-4D10 amino acids are outlined in black.


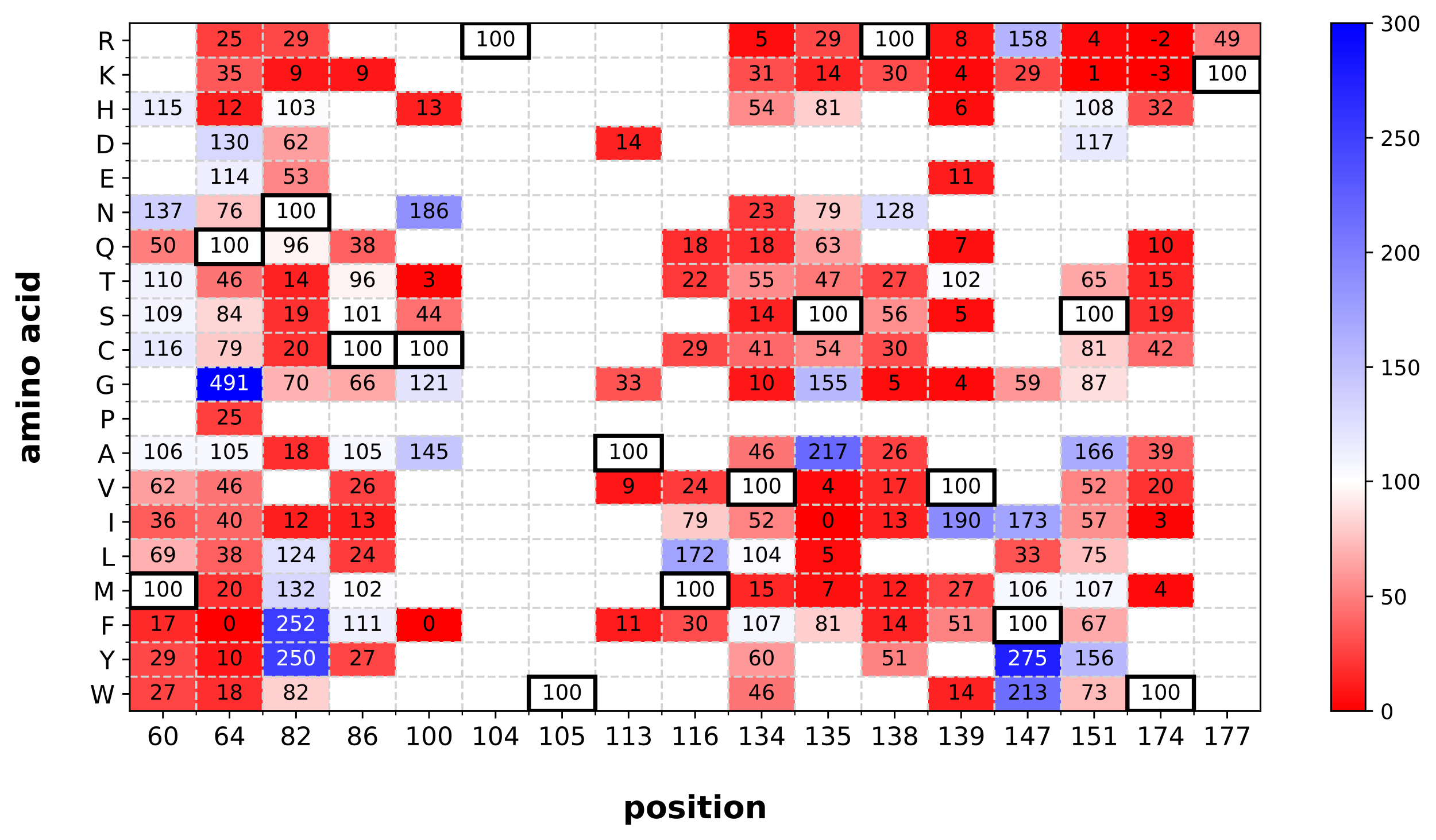


Supplementary Fig. 33. Site-saturation mutagenesis of L8-3F01. The y-axis shows the 20 canonical amino acids. Each column shows one of the 17 positions subjected to saturation mutagenesis. Each cell shows the effect of the mutation on the repressor’s response to 20 ng/mL Cs after 48 hrs incubation relative to the parental L8-3F01 (percent), quantified using a custom ImageJ script. Mutations that led to increased activation are highlighted in blue; mutations that caused decreased response are highlighted in red. If the mutation was not tested, made the protein insensitive to 20 ng/mL Cs (signal in the presence of 20 ng/mL Cs less than 10% above background), or if it increased the leakiness of the repressor > 5x compared to L8-3F01, its effect on activation was not assessed, and the corresponding cells were left blank. Original L8-3F01 amino acids are outlined in black.


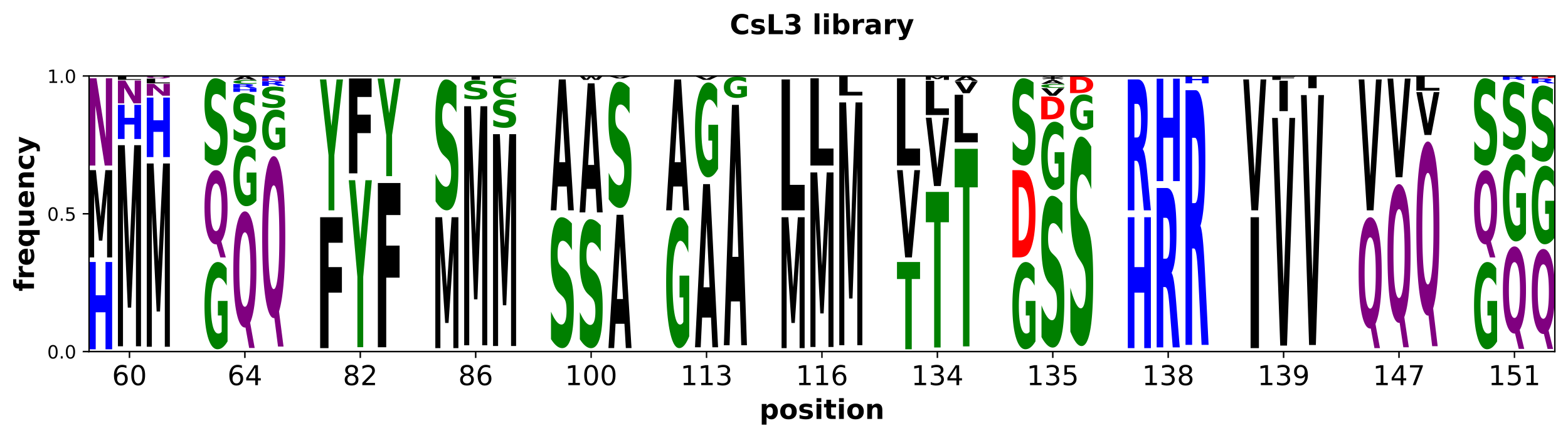


Supplementary Fig. 34. Round 5 library design, construction, and screening results. For each of the 13 positions mutated, shown are the theoretical amino acid frequencies of the encoded library (column 1), and experimental frequencies of random colonies (column 2) and selected hits (column 3).


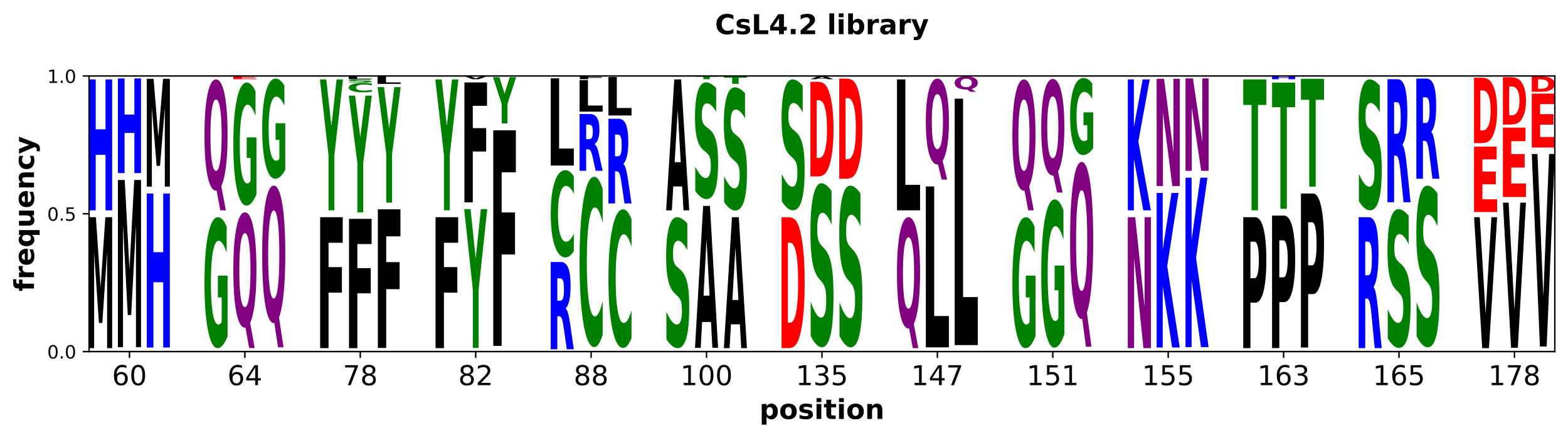


Supplementary Fig. 35. Round 6 library design, construction, and screening results. For each of the 13 positions mutated, shown are the theoretical amino acid frequencies of the encoded library (column 1), and experimental frequencies of the randomly picked colonies (column 2) and selected hits (column 3).

**Supplementary Note 1. Additional rounds of library design and screening**

Since the L1-9 + Es pair elicited the strongest response among all TetR mutant + SU pairs tested in the first round, and L1-9 had acceptably low leakiness, we decided to initially focus on developing an Es repressor.

**Second-round Es library design and screening**

For the second round, we drew diversity from naturally evolved TetR family members. The sequences of TetR class B from transposon Tn10 (used as a starting template in this study) and 33 homologs (mostly from class B, with others from classes A, D, E, and H) were aligned (Supplementary Fig. 7), and 38 positions distributed across the length of the LBD were selected. The natural sequence diversity at these positions was encoded in 73 oligonucleotides (Supplementary Data 3). To decrease the number of mutations incorporated into each clone, the oligonucleotides encoding the parent clone L1-9 (Supplementary Data 4) were also added to the pool at varying concentrations (see Methods for details).

Following the cloning of this library into the pVER7314 vector, library diversity analysis was performed by sequencing 196 random clones grown on LB plates containing carbenicillin and kanamycin. These data revealed, as intended, sparse incorporation of natural diversity, with 0-2 targeted mutations per clone (Supplementary Fig. 19a). Several mutations at non-targeted positions were also observed (Supplementary Fig. 19b).

Approximately 20,000 colonies from the prescreen plates were then tested for activity at 0, 0.2 (a 100x increase in assay stringency over the 1^st^ round), and 1 μg/mL Es on M9 (X-gal)-containing assay plates, allowing for chromogenic screening of repressor activity. More than 100 putative hits were rearrayed and retested at 0 and 0.2 μg/mL Es and assigned a score of 1-4 for both activation and leakiness, based on visual inspection of colony color (1 for high leakiness and/or low activation, 4 for low leakiness and high activation). In parallel, all putative hits were sequenced, and amino acid frequencies were compared to random colonies (Supplementary Fig. 20).

We noticed a higher-than-expected (based on random colony sequencing) substitution rate among hit clones at targeted positions 137, 140, 162, and 170, and at two unintended positions - 108 and 138 (likely originating from PCR or primer synthesis errors). We then evaluated whether any mutations at all these positions correlated with clone performance (i.e., activation and/or leakiness). Indeed, we found several trends. C138G was highly enriched in clones with relatively high activation and low leakiness. Interestingly, while L170I was the most abundant substitution across all hits, the less frequent L170V mutation was more enriched among the best-performing hits. Similarly, T162Q was common across all hits but uncommon in the best hits. K108Q, another unintended substitution, frequently appeared in clones with the highest ligand response but somewhat leaky expression. Interestingly, in one instance, the addition of a single mutation - L55M - to a K108Q-containing clone lowered the leakiness without much effect on inducer response. Among all hits, L4-118 was the most sensitive to Es and the least leaky, and was considered the best round-two hit (Supplementary Data 2).

**Third-round Es library design and screening**

In the third round, we explored the effects of combining apparently beneficial mutations found in L4 screening. We included the top three mutations (K108Q, C138G, and L170V) as well as L55M, N129H, V137A, and F140Y. We believed that the latter four may also have positively contributed to variants' fitness, but their insufficient occurrence prevented conclusive determination in the previous round. We also recruited six new residues in proximity to the binding pocket: 67, 109, 112, 117, 131, and 173. Amino acids in these positions are located at the edges of the binding pocket and may interact with the amino acids coordinating the Es, or the Es itself if it assumes a pose substantially different than that of native ligand in the parental protein. Tested diversity was selected based on manual inspection of the first round docked models. This new library, L7, with ~200,000 theoretical sequence diversity was encoded in 30 oligonucleotides (Supplementary Data 5, Supplementary Fig. 21), assembled, and cloned into pVER7314. This library was transformed into KM3 cells and subjected to repressor prescreening as before, followed by library quality control through sequencing of random colonies (Supplementary Fig. 21).

Approximately 20,000 clones were then tested for β-gal activity on 20 ng/mL Es (further 10-fold increase of the assay stringency from the round two library). 330 clones were selected as hits, rearrayed, sequenced, and replated on 0 and 20 ng/mL Es. The plates were imaged and analyzed using a custom ImageJ script, providing two scores (integers 1-5) representing each colony’s relative activation by 20 ng/mL Es and its leakiness.

Sequence-activity relationships for the L7 library were analyzed in two ways. First, we compared incorporation frequencies of different amino acids between hits and random clones (Supplementary Fig. 21). Then we assessed the contributions of individual mutations through regression analysis of the two β-gal assay scores (Supplementary Fig. 22).

Confirming the conclusions from the L4 library analysis, mutations K108Q, C138G, and L170V all showed strong enrichment, being found in 83, 92, and 69% of hits vs. 55, 52, and 15% of random clones, respectively (Supplementary Fig. 21). Regression analysis showed that all three increased Es-induced activation (Supplementary Fig. 22a), and K108Q also somewhat decreased leakiness (Supplementary Fig. 22b). In addition, position 109 was strongly retained as glutamine (66% of hits vs. 36% of random clones), and tyrosine was preferred at position 67, although less dramatically (72% vs. 53%, Supplementary Fig. 21).

Mutations L55M and F140Y, and wild-type T112 were modestly enriched in hits (Supplementary Fig. 21), thus we performed additional analysis. F140Y seemed to both slightly improve activation and decrease leakiness (Supplementary Fig. 22). Regression analysis results for positions 55 and 112 might have been skewed by two clones with isoleucine residues in those locations, so we analyzed the fraction of each amino acid in each activation or leakiness score bin instead (Supplementary Fig. 23). Surprisingly, despite only modest preference for methionine in position 55 in hits overall, it correlated with reduced leakiness. We saw no obvious preference at position 112.

One hit, denoted L7-A11, scored 5 and 1 in relative activation by 20 ng/mL Es and leakiness, respectively, and was deemed the best hit of this round. Notably, L7-A11 contained all 6 mutations (L55M, F67Y, K108Q, C138G, F140Y, and L170V) that were strongly rated as beneficial in this round of selection and no additional mutations (Supplementary Data 2). Quantitative β-gal activity assay showed ~150-fold improvement of L7-A11 over L4-118, the best round two hit (Table 1, Supplementary Fig. 8). Despite this substantial improvement, L7-A11 showed lower activation and higher leakiness than the wild-type TetR-Atc system (Table 1, Supplementary Fig. 8). Thus, we continued with more rounds of design and screening.

**Fourth-round Es library design and screening**

We sought to further improve L7-A11 using two approaches in parallel: site-saturation mutagenesis of the 17 positions mutated during the first round, and another sparse screen at many positions (including sites not yet mutated).

*Site-saturation mutagenesis of L7-A11*

We generated all 323 point mutants of L7-A11 at the 17 positions constituting the ligand-binding pocket (Supplementary Data 6). Es-induced activation and leakiness were assessed using the β-gal activity plate assay with 0 and 5 ng/mL Es. Each mutant was plated in triplicate alongside L7-A11, colony color intensity was calculated with the ImageJ script, and the three measurements were averaged.

The results (Supplementary Fig. 24) suggested that L60, G104, Q135, G138, I139, L147, and L151 were critical for L7-A11 function; positions 86, 100, and 105 allowed several substitutions that modestly increased activation by Es; and positions 64, 82, 113, 116, 134, 174, and 177 allowed multiple mutations, some of which substantially increased Es-induced activation. The latter were considered for incorporation into further libraries.

*Sparse mutagenesis of L7-A11*

For this round of sparse mutagenesis, we selected all six cysteine-containing wtTetR positions (68, 88, 121, 144, 195, and 203) and 37 random positions. 27 of these positions were screened in at least one previous library, and 16 were new (Supplementary Fig. 5). We created two libraries, L10 (Supplementary Data 7) and L11 (Supplementary Data 8), each diversified all six cysteines, and 20 other positions distributed along the length of the gene to reduce mutagenesis of consecutive residues. The tested diversity was chosen based on the results of the previous libraries, phylogenetic diversity (Supplementary Fig. 7), and manual evaluation of the first round docked models. Oligos encoding parental clone L7-A11 with only the six cysteines mutated (Supplementary Data 9) were included in the gene assembly reaction to minimize the mutagenic load, as was done before for the L4 library. To prepare for testing hits *in planta,* all oligonucleotides from this point on reflected plant codon usage. We also codon-optimized the sequence of the TetR DNA-binding domain in pVER7314, creating the new vector pVER7334.

Following library assembly, cloning, and active repressor pre-screening, 223 putative hits (99 and 124 from libraries L10 and L11, respectively) were identified from ~20,000 screened clones. These clones were rearrayed, and their leakiness and derepression were assessed by the β-gal plate assay on 0, 1.5, and 7.5 ng/mL Es. All putative hits and 189 random clones were sequenced and compared to analyze sequence-activity relationships (Supplementary Fig. 25).

Library L10 hits were enriched in P69L, E73A, and N82K substitutions; C144 was strongly retained and V99, Q109, A113, and V170 were mostly retained (Supplementary Fig. 25 top). Almost all library L11 hits retained G104, F105, Q108, A113, Q135, G138, Y140, C144, L147, L151, and K177, while C121T and C203A mutations were enriched in hits (Supplementary Fig. 25 bottom). These results confirmed many of the earlier L7-A11 site-saturation mutagenesis (SSM) observations (Supplementary Fig. 24): residues G104, G138, L147, and L151 were completely conserved; K was preferred over the tested L, R, and Q at position 177; A was favored over T, L, V or I at position 113; and mutation N82K increased activation. The three best hits from this round, L10-B07, L10-B08, and L11-C06 (Supplementary Data 2), retained L7-A11 identity at positions 99, 104, 105, 108, 109, 113, 135, 138, 140, 144, 147, 151, 170, and 177, and contained mutation C121T. L10-B08, which showed the highest Es responsiveness (Table 1, Supplementary Fig. 8), also carried E73A, N82K, and C203A substitutions.

**Fifth-round Es library design and screening**

In the fifth round, we focused on lowering repressor leakiness. We made a new library vector, pVER7571, by mutating the ribosome binding site (RBS) of pVER7334 site (AggAGA 🡪 AcAGA) to decrease basal repressor expression level, thus enhancing the sensitivity of the assay to leakiness (fewer repressor molecules must bind more tightly to *TetO* sites to achieve the same level of background transgene expression as before). Two new libraries, L12 and L13, were created in pVER7571 based on results from L10 & L11 libraries and L7-A11 SSM, respectively. The L12 library had position 77, and 5 out of 6 wild-type cysteine residues (all but position 144) mutated to an amino acid from the top L10 & L11 hits and encoded limited variability (2-3 amino acids) at another 9 positions. The L13 library rescreened beneficial mutations from L7-A11 SSM and alternatives for three wild-type cysteines and one previously introduced cysteine; the beneficial mutations C88N and C121T from L11-C06, and the strongly hit-enriched wild-type C144 were retained (Supplementary Fig. 26, Supplementary Data 10, Supplementary Data 11).

Approximately 10,000 clones from each library were screened using the β-gal plate assay with 0 or 2 ng/mL Es. As the RBS mutation dramatically reduced repressor expression, we had to supplement the Es-containing plates with 0.001-0.002% arabinose to increase repressor concentration and, thus, raise the stringency of the derepression assay.

From library L12, we discovered and sequenced 43 hits, alongside 94 random clones (Supplementary Fig. 26 top). Comparison of the sequences showed that mutations T65I, F67Y, and I174W were preferred, whereas wild-type residues I57, R62, P69, E73, and N82 were mostly retained. Interestingly, for most positions with clear preference among hits (especially 62 and 69), this preference was already observable in the random clones, and might have been the result of increased selection pressure for lower leakiness.

From library L13, we discovered and sequenced 60 hits, alongside 78 random clones (Supplementary Fig. 26 bottom). Their comparison showed some preference for C100A and I174Y, strong retention of M86, and selection against 82N (in favor of K or R), 116W (in favor of S or C), 134G (in favor of M or F), and 177H (in favor of R or K). Interestingly, position 82 - common between the two libraries - showed the opposite preference in L13 from the L12 library, where N was strongly favored over K. This is perhaps caused by the different sequence context. The bias in library L13 random clones was less pronounced than in the L12 library.

Out of the three best round five hits, denoted L12-11, L13-9, and L13-23 (Supplementary Data 2), two (L12-11 and L13-9) showed further lowering of Es EC_50_, and L13-23 demonstrated the best selectivity for Es over other SUs (Table 1, Supplementary Fig. 8, Supplementary Fig. 9, Supplementary Fig. 12).

**Sixth-round Es library design and screening**

In the sixth round of Es-responsive repressor evolution, we explored diversity found in the best hits from round five (Supplementary Fig. 27, Supplementary Data 12). 7,500 clones were screened using the plate β-gal assay for inducer-free repression and for activation in the presence of 2 ng/mL Es. We sequenced 44 putative hits and 92 randomly selected clones. Sequence analysis (Supplementary Fig. 27) showed that 82N, 116W, and to a lesser extent 174Y, were selected against relative to other residues. 110C, 116S, and 177A were preferred among hits, while 62R was again strongly selected even in random clones. However, the top performing hit, L15-20 (Supplementary Data 2), did not show much improvement over previous rounds’ hits when retested in the liquid β-gal assay (Table 1, Supplementary Fig. 8). Thus, we stopped optimizing the Es-responsive repressors at this point, reasoning that we had reached diminishing returns phase.

**Library design and screening for other inducers**

After establishing the library design and screening methodology during the development of the Es repressor - resulting in EsRs with very high sensitivity to Es and low leakiness, we next pursued repressors for other SU herbicides. Of particular interest were metsulfuron-methyl (Ms), sulfometuron-methyl (Sm), and chlorsulfuron (Cs), as these herbicides, along with Es, are approved for use on many of the most important crops worldwide, including maize, soybean, canola, and cotton. As a starting point for engineering, we selected a handful of first-round clones that had shown promise for responsivity to those alternative inducers: L1-9, L1-29, and L1-44 responded to Cs, L1-22 responded to Sm, and L1-29 and L1-44 responded to Ms (Supplementary Fig. 4). The full amino acid sequence diversity of the four selected first round clones was encoded in 102 oligonucleotides (Supplementary Data 13).

This library, denoted L2, was assembled, cloned, assessed for quality and sequence diversity (Supplementary Fig. 28), and screened like library L1, except that each of the three new target ligands was tested at 2 μg/mL concentration (10-fold lower) to increase assay stringency.

The color of ~10,000 colonies was visually inspected in the presence and absence of different SUs on M9 X-gal assay plates. 20 putative hits were rearrayed and retested.

Two clones, L2-14 and L2-18 (Supplementary Data 2), showed improved and highly selective derepression by Cs, while retaining strong repression activity (i.e. low leakiness). No improvement was seen for Ms and Sm, so we focused on optimizing response to Cs.

**Third-round Cs library design and screening**

In the next round, we explored the diversity provided by the previous round top hits L2-14 and L2-18, complemented by diversity at positions 67, 109, 112, and 173 previously tested in L7 library (Es library). We also tested 108Q and 170V/A substitutions, found in library L4 to be associated with high SU responsiveness. The oligonucleotides designed to generate this library, L6, are shown in Supplementary Data 14.

Library L6 was assembled, ligated into the pVER7314 vector, transformed, and plated as before. After confirming library quality (Supplementary Fig. 29), ~20,000 clones were stamped onto M9 X-gal plates containing 0, 0.2, or 2.0 μg/mL Cs. Following incubation at 30 °C for ~48 hours, ~550 putative hits (chosen by visual inspection) were rearrayed and re-analyzed. To facilitate clone prioritization, rearrayed plates were imaged at various time points of incubation, and images were analyzed using a custom ImageJ script. In parallel, all clones were sequenced and the amino acid incorporation frequencies analyzed (Supplementary Fig. 29).

To help determine the contribution of individual mutations to the repressor’s background leakiness or/and activation by Cs, we performed a regression analysis of the quantified β-gal activity plate assay results (Supplementary Fig. 30). Both the amino acid incorporation frequencies (Supplementary Fig. 29) and the regression analysis results indicated a benefit from substitutions 100C, 138R, 139V, and 174W. Additionally, the regression analysis highlighted a strong correlation of high activation with 109Q and to a lesser extent 108Q, and of low leakiness with 170L and 173A. These observations guided the design of the next library.

One of the top hits of this library, L6-4D10 (Supplementary Data 2), demonstrated an almost 10x increase in β-gal activity over background after incubation with 0.2 μg/mL of Cs.

**Fourth-round Cs library design and screening**

The fourth round of evolution further explored some L6 library trends (positions 100, 112, 139, and 170), attempted to reduce the number of cysteine residues (positions 68, 88, 121, 144, 195, and 203) using TetR family phylogenetic information (Supplementary Fig. 7), tested more of the L7 library (Es library) diversity (positions 55, 117, 131, and 137), and retested several main binding pocket residues (positions 60, 64, 82, 86, 104, 105, 113, 134, 135, 147, and 177). Codon usage was adjusted to resemble that found in plants. The fully synthetic library was constructed from oligonucleotides (Supplementary Data 15). As the theoretical diversity of the library was very high, to decrease the number of changes per clone, the library-encoding oligonucleotides were mixed at 5:95, 10:90, and 25:75 ratios with those encoding the L6 library hit clone L6-4D10 with only the six cysteine-containing positions diversified (Supplementary Data 16). The resulting library (L8) was cloned into pVER7334, which contains plant codon-optimized sequence for the TetR DNA-binding domain.

From ~15,000 clones assessed on M9 X-gal plates containing 0 or 0.2 μg/mL Cs, 248 putative hits were selected and rearrayed. All hits along with random clones from each of the three libraries (5:95, 10:90, and 25:75 oligo ratio) were sequenced and compared to check for trends in amino acid incorporation (Supplementary Fig. 31). In addition, the 32 best performing hits were rearrayed again and imaged after 24 hours (with Cs) and 48 hours (without Cs). The images were analyzed using a custom ImageJ script, and the scores were used for the regression analysis (Supplementary Fig. 32).

The trend in the overall population of hits (first rearray, Supplementary Fig. 31) was to maintain the parental L55, Q64, R104, W105, L117, L170, and K177, while mutating C144 to A and C195 and C203 to non-cysteine amino acids (with a potential preference to R over A and S in both cases). The regression analysis of top hit sequences and the corresponding leakiness and Cs activation scores (second rearray, Supplementary Fig. 32) supported the benefits of C144A and mutating C195 and C203 (with R and A being the best alternative amino acids). Another two cysteines, 68 and 88, tolerated mutation, but at the cost of increasing leakiness. V139I, V134L, V137A, and N82L also correlated with better clone performance. Surprisingly, the top hit of this round, L8-3F01 (Supplementary Data 2), harbored mutations at only several of these positions, and often to amino acids other than those trending in most hits.

**Site-saturation mutagenesis of the ligand-binding pocket**

To generate diversity for the next library, the 17 core residues of the wild-type ligand-binding pocket (60, 64, 82, 86, 100, 104, 105, 113, 116, 134, 135, 138, 139, 147, 151, 174, and 177) in L8 library hit L8-3F01 were subjected to site-saturation mutagenesis (Supplementary Data 17).

Mutants were transformed into KM3, and 96 colonies from each plate were sequenced. Colonies representing each possible side-chain at each position (except for 17 that were not found) were rearrayed in triplicate onto M9 X-gal assay plates with 0 or 20 ng/mL Cs. Plates were incubated at 37 °C for 48 hrs before imaging. Residue substitutions were then ranked by activation and repression characteristics using a custom ImageJ script (Supplementary Fig. 33).

The greatest improvement in activity was achieved by replacing residue N82 with phenylalanine or tyrosine. Substitutions Q64G, S135A, S135D, S135E, F147Q, F147V, F147Y, and S151Q dramatically increased induction by Cs, but at the expense of a substantial increase in leakiness. Amino acids R104, W105, A113, W174, and K177 appeared crucial for L8-3F01 function, as no mutations were tolerated.

**Fifth-round Cs library design and screening**

The CsL3 library was constructed to rescreen top-performing substitutions from L8-3F01 saturation mutagenesis and evaluate their combined effects (Supplementary Data 18). To facilitate downstream purification of hit proteins, a 6xHis-tag was incorporated at the C-terminus of the protein during library assembly.

Approximately 10,000 colonies were picked into 384-well plates and replica-stamped onto M9 X-gal assay plates containing 0 or 20 ng/mL Cs. 110 putative hits were rearrayed and replated. Colony color was assessed at 24 (with Cs) and 96 hrs (without Cs) of incubation at 37 °C. In parallel, all putative hits and a set of random clones were sequenced.

Analysis of amino acid substitution frequencies in the random and hit populations (Supplementary Fig. 34) suggested maintenance of residues Q64, A113, M116, S135, R138, and V139 and incorporation of substitutions V134T and F147Q. Interestingly, an unintended F147L mutation was seen in three top hits and associated with a ~ 2x increase in activation over background compared to the next best clones.

**Creating novel diversity through random mutagenesis**

To explore diversity at more positions, the ligand-binding domain of one of the top CsL3 library hits, CsL3-C12 (Supplementary Data 2), was subjected to error-prone PCR mutagenesis. Approximately 10,000 colonies were then replica-plated onto M9 X-gal assay plates containing 0 or 20 ng/mL Cs. Putative hits were then rearrayed and replica-plated onto fresh plates with 0 or 20 ng/mL Cs. Colonies color was assessed after 24 hrs of incubation with Cs and 72 hrs of incubation without inducer, and the best-performing variants were sequenced.

Position 178 was mutated (to either V or E) in most hits. Substitutions at new positions (F78Y and S165R) and a previously mutated position (R88C) were also seen.

**Sixth-round Cs library design and screening**

The sixth-round library, designated CsL4.2, was designed to rescreen the diversity at 13 positions inspired by the CsL3 library and the random mutagenesis results (Supplementary Data 19). The library also contained the C-terminal 6xHis-tag. Approximately 8,000 colonies were rearrayed in 384-well format and replica-plated onto M9 X-gal assay plates with 0 or 5 ng/mL Cs. Putative hits were rearrayed in 96-well format and replica-plated onto the same plates for retesting. 34 confirmed hits were sequenced. Analysis of amino acid incorporation (Supplementary Fig. 35) showed that retention of F82, and mutations 147L, 178V, and to a lesser extent 151Q were strongly selected in hits. Although there was no obvious preference at position 135 in the larger hit population, all top six clones had S135D. The best library hits, CsL4.2-20 and CsL4.2-15 (Supplementary Data 2), had EC_50_ similar to the most sensitive EsRs (Table 1, Supplementary Fig. 10). As such, we deemed the construction of CsRs successful and stopped optimization.

**Supplementary references**

1. Gurskaya, N. G. *et al.* A colourless green fluorescent protein homologue from the non-fluorescent hydromedusa Aequorea coerulescens and its fluorescent mutants. *Biochem. J.* **373**, 403–408 (2003).

2. Bevis, B. J. & Glick, B. S. Rapidly maturing variants of the Discosoma red fluorescent protein (DsRed). *Nat. Biotechnol.* **20**, 83–87 (2002).

3. Betts, S. D. *et al.* Uniform Expression and Relatively Small Position Effects Characterize Sister Transformants in Maize and Soybean. *Front. Plant Sci.* **10**, 1209 (2019).

4. Gordon-Kamm, W. *et al.* Stimulation of the cell cycle and maize transformation by disruption of the plant retinoblastoma pathway. *Proc. Natl. Acad. Sci. U. S. A.* **99**, 11975–11980 (2002).

5. Matsui, K., Umemura, Y. & Ohme-Takagi, M. AtMYBL2, a protein with a single MYB domain, acts as a negative regulator of anthocyanin biosynthesis in Arabidopsis. *Plant J. Cell Mol. Biol.* **55**, 954–967 (2008).

6. Bourett, T. M., Sweigard, J. A., Czymmek, K. J., Carroll, A. & Howard, R. J. Reef coral fluorescent proteins for visualizing fungal pathogens. *Fungal Genet. Biol. FG B* **37**, 211–220 (2002).

7. Zheng, X. *et al.* The cauliflower mosaic virus (CaMV) 35S promoter sequence alters the level and patterns of activity of adjacent tissue- and organ-specific gene promoters. *Plant Cell Rep.* **26**, 1195–1203 (2007).

8. Gatz, C., Frohberg, C. & Wendenburg, R. Stringent repression and homogeneous de-repression by tetracycline of a modified CaMV 35S promoter in intact transgenic tobacco plants. *Plant J. Cell Mol. Biol.* **2**, 397–404 (1992).

9. Li, Z. Soybean EF1A2 promoter and its use in constitutive expression of transgenic genes in plants. (2014).

10. Kalla, R. *et al.* The promoter of the barley aleurone-specific gene encoding a putative 7 kDa lipid transfer protein confers aleurone cell-specific expression in transgenic rice. *Plant J. Cell Mol. Biol.* **6**, 849–860 (1994).

11. An, G. Development of Plant Promoter Expression Vectors and Their Use for Analysis of Differential Activity of Nopaline Synthase Promoter in Transformed Tobacco Cells. *Plant Physiol.* **81**, 86–91 (1986).

12. Callis, J., Carpenter, T., Sun, C. W. & Vierstra, R. D. Structure and evolution of genes encoding polyubiquitin and ubiquitin-like proteins in *Arabidopsis thaliana* ecotype Columbia. *Genetics* **139**, 921–939 (1995).
