# Supplementary material for: Design and evolution of the tetracycline repressor into sulfonylurea herbicide-responsive gene switches for field crops
Source: Nat Commun. 2026 Jun 8;17:7278. doi: 10.1038/s41467-026-73848-w (PMC13402354; doi:10.1038/s41467-026-73848-w)
Supplement: Supplementary file 3 — Description of Additional Supplementary Files [file 41467_2026_73848_MOESM3_ESM.pdf]

Description of Additional Supplementary Files

File Name: Supplementary Data 1
Description: Oligonucleotides used to construct library L1.

File Name: Supplementary Data 2
Description: Protein sequences of the best hits from each library.

File Name: Supplementary Data 3
Description: Oligonucleotides used to construct library L4.

File Name: Supplementary Data 4
Description: Oligonucleotides used to encode the parent clone L1-9.

File Name: Supplementary Data 5
Description: Oligonucleotides used to construct library L7.

File Name: Supplementary Data 6
Description: Oligonucleotides used to generate point mutants of hit clone L7-A11 at the 17 positions constituting the ligand-binding pocket.

File Name: Supplementary Data 7
Description: Oligonucleotides used to construct library L10.

File Name: Supplementary Data 8
Description: Oligonucleotides used to construct library L11.

File Name: Supplementary Data 9
Description: Oligonucleotides used to encode hit clone L7-A11 with the six cysteines mutated.

File Name: Supplementary Data 10
Description: Oligonucleotides used to construct library L12.

File Name: Supplementary Data 11
Description: Oligonucleotides used to construct library L13.

File Name: Supplementary Data 12
Description: Oligonucleotides used to construct library L15.

File Name: Supplementary Data 13
Description: Oligonucleotides used to construct library L2.

File Name: Supplementary Data 14
Description: Oligonucleotides used to construct library L6.

File Name: Supplementary Data 15
Description: Oligonucleotides used to construct library L8.

File Name: Supplementary Data 16
Description: Oligonucleotides used to encode hit clone L6-4D10 with the six cysteines diversified.

File Name: Supplementary Data 17
Description: Oligonucleotides used to generate point mutants of hit clone L8-3F01 at the 17 positions constituting the ligandbinding pocket.

File Name: Supplementary Data 18
Description: Oligonucleotides used to construct library CsL3.

File Name: Supplementary Data 19
Description: Oligonucleotides used to construct library CsL4.2.
