# Supplementary material for: Design and evolution of the tetracycline repressor into sulfonylurea herbicide-responsive gene switches for field crops
Source: Nat Commun. 2026 Jun 8;17:7278. doi: 10.1038/s41467-026-73848-w (PMC13402354; doi:10.1038/s41467-026-73848-w)
Supplement: Supplementary file 23 — Reporting Summary [file 41467_2026_73848_MOESM23_ESM.pdf]

Corresponding author(s): Kevin E. McBride, William Gordon-Kamm, Loren L. Looger

Last updated by author(s): May 5, 2026

Reporting Summary

Nature Portfolio wishes to improve the reproducibility of the work that we publish. This form provides structure for consistency and transparency in reporting. For further information on Nature Portfolio policies, see our Editorial Policies and the Editorial Policy Checklist.

Statistics

For all statistical analyses, confirm that the following items are present in the figure legend, table legend, main text, or Methods section.

n/a	Confirmed	
☐	☒	The exact sample size (*n*) for each experimental group/condition, given as a discrete number and unit of measurement
☐	☒	A statement on whether measurements were taken from distinct samples or whether the same sample was measured repeatedly
☐	☒	The statistical test(s) used AND whether they are one- or two-sided<br>*Only common tests should be described solely by name; describe more complex techniques in the Methods section.*
☒	☐	A description of all covariates tested
☒	☐	A description of any assumptions or corrections, such as tests of normality and adjustment for multiple comparisons
☐	☒	A full description of the statistical parameters including central tendency (e.g. means) or other basic estimates (e.g. regression coefficient) AND variation (e.g. standard deviation) or associated estimates of uncertainty (e.g. confidence intervals)
☐	☒	For null hypothesis testing, the test statistic (e.g. *F*, *t*, *r*) with confidence intervals, effect sizes, degrees of freedom and *P* value noted<br>*Give P values as exact values whenever suitable.*
☒	☐	For Bayesian analysis, information on the choice of priors and Markov chain Monte Carlo settings
☒	☐	For hierarchical and complex designs, identification of the appropriate level for tests and full reporting of outcomes
☒	☐	Estimates of effect sizes (e.g. Cohen's *d*, Pearson's *r*), indicating how they were calculated

Our web collection on statistics for biologists contains articles on many of the points above.

Software and code

Policy information about availability of computer code

Data collection	BD FACSDiva (v9.0.2), SOFTmax PRO (v4.6), SparkControl (v3.1 SP1), BZ-X800 Viewer (v1.03.00.01), Octet Data Acquisition Software (v6.4.0.137)
Data analysis	FlowJo 10.10.0, GraphPad Prism 10, Python 3, PyMol (v2.3.0), Chem3D 9, Microsoft Excel, ImageJ/Fiji, d*Trek, MOSFLM, Phaser, Coot, Refmac5, Octet Data Analysis Software (v6.4.0.137), XnView. The custom code for running computational protein modeling and design software Chameleon and the ImageJ script are available at https://github.com/LorenLoogerUCSD/SURepressors. A snapshot of the code is provided on Zenodo [https://zenodo.org/records/19933159].

For manuscripts utilizing custom algorithms or software that are central to the research but not yet described in published literature, software must be made available to editors and reviewers. We strongly encourage code deposition in a community repository (e.g. GitHub). See the Nature Portfolio guidelines for submitting code & software for further information.

Data

Policy information about availability of data

All manuscripts must include a data availability statement. This statement should provide the following information, where applicable:

- Accession codes, unique identifiers, or web links for publicly available datasets
- A description of any restrictions on data availability
- For clinical datasets or third party data, please ensure that the statement adheres to our policy

The authors declare that all data supporting the findings of this study are available within the Article and its Supplementary Information files, Supplementary Data

files, and Source Data. The crystal structures of EsR(L7-D1) apo, EsR(L11-C6)-Es, CsR(L4.2-20) apo, and CsR(L4.2-20)-Cs generated in this study have been deposited in the PDB under accession codes 9DT2 [https://doi.org/10.2210/pdb9DT2/pdb], 9DT3 [https://doi.org/10.2210/pdb9DT3/pdb], 9DT4 [https://doi.org/10.2210/pdb9DT4/pdb], and 9DT5 [https://doi.org/10.2210/pdb9DT5/pdb]. Previously published structures used in this study are available in the PBD under accession codes 1A6I [https://doi.org/10.2210/pdb1A6I/pdb], 1BJ0 [https://doi.org/10.2210/pdb1BJ0/pdb], 1BJZ [https://doi.org/10.2210/pdb1BJZ/pdb], 1DU7 [https://doi.org/10.2210/pdb1DU7/pdb], 1N0H [https://doi.org/10.2210/pdb1N0H/pdb], 1ORK [https://doi.org/10.2210/pdb1ORK/pdb], 1QPI [https://doi.org/10.2210/pdb1QPI/pdb], 1T9A [https://doi.org/10.2210/pdb1T9A/pdb], 1T9B [https://doi.org/10.2210/pdb1T9B/pdb], 1T9C [https://doi.org/10.2210/pdb1T9C/pdb], 1T9D [https://doi.org/10.2210/pdb1T9D/pdb], 1YBH [https://doi.org/10.2210/pdb1YBH/pdb], 1YHY [https://doi.org/10.2210/pdb1YHY/pdb], 1YHZ [https://doi.org/10.2210/pdb1YHZ/pdb], 1YI0 [https://doi.org/10.2210/pdb1YI0/pdb], 1YI1 [https://doi.org/10.2210/pdb1YI1/pdb], 2NS7 [https://doi.org/10.2210/pdb2NS7/pdb], 2NS8 [https://doi.org/10.2210/pdb2NS8/pdb], 2O7O [https://doi.org/10.2210/pdb2O7O/pdb], 2TCT [https://doi.org/10.2210/pdb2TCT/pdb], 2TRT [https://doi.org/10.2210/pdb2TRT/pdb], 2VKE [https://doi.org/10.2210/pdb2VKE/pdb], 2VPR [https://doi.org/10.2210/pdb2VPR/pdb], 2VKV [https://doi.org/10.2210/pdb2VKV/pdb], 3E9Y [https://doi.org/10.2210/pdb3E9Y/pdb], 3EA4 [https://doi.org/10.2210/pdb3EA4/pdb], 4AC0 [https://doi.org/10.2210/pdb4AC0/pdb], 5FEM [https://doi.org/10.2210/pdb5FEM/pdb], 6DEL [https://doi.org/10.2210/pdb6DEL/pdb], 6DEM [https://doi.org/10.2210/pdb6DEM/pdb], 6DEN [https://doi.org/10.2210/pdb6DEN/pdb], 6DEP [https://doi.org/10.2210/pdb6DEP/pdb], 7STQ [https://doi.org/10.2210/pdb7STQ/pdb], 7U1D [https://doi.org/10.2210/pdb7U1D/pdb], 7Y0L [https://doi.org/10.2210/pdb7Y0L/pdb], 7YD2 [https://doi.org/10.2210/pdb7YD2/pdb], 8GOL [https://doi.org/10.2210/pdb8GOL/pdb], 8IVE [https://doi.org/10.2210/pdb8IVE/pdb], 8IVM [https://doi.org/10.2210/pdb8IVM/pdb], 8IVN [https://doi.org/10.2210/pdb8IVN/pdb], 8IVS [https://doi.org/10.2210/pdb8IVS/pdb], 8IVT [https://doi.org/10.2210/pdb8IVT/pdb], 8IW3 [https://doi.org/10.2210/pdb8IW3/pdb], 8IW6 [https://doi.org/10.2210/pdb8IW6/pdb], 8J7I [https://doi.org/10.2210/pdb8J7I/pdb], and 8J7L [https://doi.org/10.2210/pdb8J7L/pdb]. Bacterial pRSET-based vectors encoding N-terminal 6xHis-tagged wtTetR, L12-11, L13-9, L13-23, L15-20, CsL4.2-15, and CsL4.2-20 as well as mammalian vectors pcDNA6/L13-9, pcDNA6/L15-20, pcDNA6/CsL4.2-15, pcDNA6/CsL4.2-20, and pcDNA4/TO-sfGFP generated in this study have been deposited at Addgene (#242345-242356). Contact Loren Looger (llooger@ucsd.edu) for additional bacterial and mammalian plasmids requests. Corteva Agriscience will provide plant plasmids to academic investigators for non-commercial research under an applicable material transfer agreement subject to proof of permission from any third-party owners of all or parts of the material and to governmental regulation considerations. Completion of a stewardship plan is also required. The Pioneer maize inbred line PH184C described in this research is proprietary.

Research involving human participants, their data, or biological material

Policy information about studies with human participants or human data. See also policy information about sex, gender (identity/presentation), and sexual orientation and race, ethnicity and racism.

Reporting on sex and gender	n/a
Reporting on race, ethnicity, or other socially relevant groupings	n/a
Population characteristics	n/a
Recruitment	n/a
Ethics oversight	n/a

Note that full information on the approval of the study protocol must also be provided in the manuscript.

Field-specific reporting

Please select the one below that is the best fit for your research. If you are not sure, read the appropriate sections before making your selection.

[x] Life sciences [ ] Behavioural & social sciences [ ] Ecological, evolutionary & environmental sciences

For a reference copy of the document with all sections, see nature.com/documents/nr-reporting-summary-flat.pdf

Life sciences study design

All studies must disclose on these points even when the disclosure is negative.

Sample size	No formal sample size calculation was performed. Sample sizes were chosen based on community standards for each experiment type. A minimum of three independent biological replicates were used, which is standard practice for establishing reproducibility of qualitative or large-magnitude effects. Because the effects demonstrated in this study (e.g., reporter gene expression levels with or without the ligand) are large in magnitude and many comparisons are qualitative rather than statistical, formal power analysis was not applicable. Sample sizes were considered sufficient to demonstrate reproducibility across independent experiments.
Data exclusions	No data were excluded in the study.
Replication	Initial protein hits were discovered through library screening and confirmed by different methods. Later, the genes were resynthesized de novo, cloned in a different vector at a different location, and recharacterized by a different investigator there using a mixture of previous and new methods. Overall, the proteins were tested in E. coli, in purified protein, in planta, and in eukaryotic cell lines independently by more than a dozen people. All testing produced consistent results. All repeated experiments yielded similar results.
Randomization	For flow cytometry experiments, the cells were grown in 24-well plates. To reduce edge effects, the location of each repressor-inducer pair was randomized before each independent experiment. Other samples were not randomly allocated to experimental groups because group membership was determined by the experimental manipulation itself (e.g., which construct or reagent was introduced). All conditions were tested in parallel under identical experimental conditions to control for batch effects and environmental covariates.
Blinding	In flow cytometry experiments, the investigator who collected and analyzed the data did not know which repressor-inducer pair is present in the analyzed sample. Before each of three independent experiments, two different (always new) people decided on the order of transfection

mixtures (columns) and ligands (rows), respectively, reported it to a records keeper, and removed all the labels. After all data were analyzed, the data were unblinded in the presence of the third investigator that was not otherwise involved in the study.

Reporting for specific materials, systems and methods

We require information from authors about some types of materials, experimental systems and methods used in many studies. Here, indicate whether each material, system or method listed is relevant to your study. If you are not sure if a list item applies to your research, read the appropriate section before selecting a response.

Materials & experimental systems

n/a	Involved in the study	
☐	☒	Antibodies
☐	☒	Eukaryotic cell lines
☒	☐	Palaeontology and archaeology
☒	☐	Animals and other organisms
☒	☐	Clinical data
☒	☐	Dual use research of concern
☐	☒	Plants

Methods

n/a	Involved in the study	
☒	☐	ChIP-seq
☐	☒	Flow cytometry
☒	☐	MRI-based neuroimaging

Antibodies

Antibodies used	Anti-DsRed-Express polyclonal antibody (Clontech, Product #632496)
Validation	This polyclonal antibody was validated by the manufacturer by Western blot analysis (https://www.takarabio.com/documents/Certificate%20of%20Analysis/632496/632496-101717.pdf?srsltid=AfmBOorQTQTIR9494avp3ZhOQfyAsjrcIy0L3ZarMLioPL24QwjOz3aY) and is cited in more than 1500 publications.

Eukaryotic cell lines

Policy information about cell lines and Sex and Gender in Research

Cell line source(s)	HEK293 cell line was obtained from ATCC (catalog #ACS-4500)
Authentication	The cell line was authenticated by ATCC. No additional authentication has been performed.
Mycoplasma contamination	The cell line tested negative for mycoplasma contamination. Cells were tested monthly.
Commonly misidentified lines (See ICLAC register)	None used.

Plants

Seed stocks	Seeds of the Pioneer Zea mays inbred line PH184C are proprietary. Seeds of Glycine max (L.) Merrill cv. Jack, Oryza sativa cv. Kitaake, Nicotiana tabacum cv. Xanthi NN, N. benthamiana cv. TW17, and Arabidopsis thaliana were obtained from DuPont Pioneer's own collection.
Novel plant genotypes	Plant vectors were assembled using appropriate promoters, terminators, and selection markers for each species and application. All genes were plant codon-optimized. All vectors but PHP45473 were designed for random integration using Agrobacterium transformation and contained insertion flanked by T-DNA borders. Instead of T-DNA borders, vector PHP45473 has AscI restriction sites for excision and isolation of the DNA construct for particle-gun bombardment-based plant transformation. Vector designs are shown in Table S3. The number of independent lines that were analyzed and the generation used for the experiments differed between experiments and are clearly stated in the manuscript.
Authentication	Transgene DNA copy number was estimated by qPCR. DNA was extracted from 200 ng fresh leaf tissue via a modified HotSHOT alkaline lysis method. Genes encoding DsRed-Express and/or HRA were quantified using sequence-specific forward and reverse primers and fluorogenic probes (FAM and MGB-based, correspondingly). Each assay was multiplexed and normalized with an endogenous gene using sequence-specific forward and reverse primers and a VIC-labeled probe. Each multiplexed assay was primer-titrated to near equal efficiencies, and reactions for test and endogenous genes were run simultaneously in a single-tube optimized reaction. Upon completion of real time qPCR, all raw data were used to calculate the ΔCt, and copy numbers were determined using the 2-ΔΔCt method.

Flow Cytometry

Plots

Confirm that:

- ☒ The axis labels state the marker and fluorochrome used (e.g. CD4-FITC).
- ☒ The axis scales are clearly visible. Include numbers along axes only for bottom left plot of group (a 'group' is an analysis of identical markers).
- ☐ All plots are contour plots with outliers or pseudocolor plots.
- ☒ A numerical value for number of cells or percentage (with statistics) is provided.

Methodology

Sample preparation	HEK293 cells were plated in 24-well plates (30,000 cells per well). The next day cells were transfected with 0.5 µg of DNA mixture and 2 µl of TurboFect according to the manufacturer's protocol. The DNA mixtures consisted of ~14:1 (w/w) pcDNA6/TR:pcDNA4/TO-sfGFP plasmid DNA ratio for experimental conditions and ~6:1 pcDNA4/TO:pcDNA4/TO-sfGFP plasmid DNA ratio for the single-stain control. Unstained/live-dead stain (propidium iodide, PI) single-stain control cells were treated similarly, but no DNA was added in the reaction mixture. 24 hours after transfection, the medium was replaced with fresh medium supplemented with 1 µg/ml Tc, Es, Cs, or the appropriate amount of DMSO (carrier solvent, max 0.1%). 24 hours after induction, the cells were harvested and resuspended in serum-free Opti-MEM with 3 µM of PI (in serum-free Optim-MEM without PI in case of unstained and single-stain sfGFP controls). Single-stain live-dead control was obtained by mixing cells that were heated at 65C for 10 min and cooled down for 1 min on ice with unheated cells. Samples were analyzed using a FACSymphony A1 Cell Analyzer (Becton Dickinson) using violet laser (405 nm) and BV480 (525/50) filter for sfGFP and yellow-green laser (561 nm) and PE-Texas Red (610/20) filter for PI detection.
Instrument	FACSymphony A1 Cell Analyzer
Software	BD FACSDiva (v9.0.2), FlowJo 10.10.0
Cell population abundance	The percentage of sfGFP-expressing cells among live cells ranged between 6% (no inducer or non-cognate inducer) and 69% (cognate inducer or no repressor control).
Gating strategy	FSC-A vs SSC-A was used to exclude debris, followed by FSC-A vs FSC-H to select single cells, PI (PE-Texas Red)-negative cells to select live cells, and sfGFP (BV480)-positive cells to quantify median intensity.

☒ Tick this box to confirm that a figure exemplifying the gating strategy is provided in the Supplementary Information.
