# Supplementary material for: Design and evolution of the tetracycline repressor into sulfonylurea herbicide-responsive gene switches for field crops
Source: Nat Commun. 2026 Jun 8;17:7278. doi: 10.1038/s41467-026-73848-w (PMC13402354; doi:10.1038/s41467-026-73848-w)
Supplement: Supplementary file 2 — Peer Review file [file 41467_2026_73848_MOESM2_ESM.pdf]

nature portfolio

Peer Review File

Design and evolution of the tetracycline repressor into sulfonylurea herbicide-responsive gene switches for field crops

Corresponding Author: Dr Loren Looger

This file contains all reviewer reports in order by version, followed by all author rebuttals in order by version.

Version 1:

Reviewer comments:

Reviewer #2

(Remarks to the Author)
Abstract
lines 29 and 32
“affinity” … binding constants determined, estimated?
lines 37-39
“The new proteins …” are not new, better variants/mutants.

Major problem:
Manuscript lines 173-174 … You have not used iso-7-chlortetracycline! Also, in Supplement Figure 1 you refer to the structure of TetR/iso-tetracycline complex with PDB entry code 1DU7. The figure shows not the iso-7-Chlortetracycline but the common 7-Chlor-tetracycline. In the PDB the entry code 1DU7 is obsolete and you are forwarded to 2X9D published in https://doi.org/10.1021/jm200332e

2X9D shows the real iso-tetracycline. 1DU7 obviously has been overwritten at least in 2011.
In iso-tetracyclines the rigid tetracycline framework (naphthacene, tetracene) is broken into two connected rigid pieces with much more conformational freedom.
With this confusion the manuscript has to be corrected, and the editor may give my name to the authors to provide some help.
By the way, I think a reference using only the PDB code is not appropriate.

According to other reviewer reports the authors spent substantial efforts to improve the manuscript.

referee #5 major comments 1), page 21 of rebuttal.
The criticism on environmental problems should answered and/or pointed out in the manuscript.
--- end of comments ---

Reviewer #5

(Remarks to the Author)
The authors responses have helped clarify the scope and context for the work presented. I have no further comments.

Reviewer #6

(Remarks to the Author)
The authors have made considerable effort to address the reviewers’ comments. While much of their response is satisfactory, a few questions remain, which are highlighted below (listed as 1, 2 and 3).

Important features of a field-compatible system include low background, high magnitude of inducer-evoked response, and

practicality of inducer application (i.e., rapid penetration and movement to target tissues/cells, lack of degradation by target plant, neutrality to growth and development, safety, photostability, and registration for field use). Only two existing plant-specific induction systems employ approved agrochemicals as inducer: ecdysone receptor-based systems (Unger et al., 2002, Padidam et al., 2003; Tavva et al., 2006) and ABA receptor PYR1-based systems (Park et al. 2015; Park et al. 2024). In the case of ABA receptor-based systems, in planta reporter gene activation was shown only in response to azinphos-ethyl - a compound banned due to its extreme toxicity. By contrast, the agriculturally relevant candidate from that paper - mandipropamid - was shown only to induce expression of RD29B, a native ABA-responsive gene, with no synthetic reporter gene induction demonstrated. The ecdysone receptor (EcR)-based systems appeared highly promising in the early 2000s during the period of their active development (Unger et al., 2002, Padidam et al., 2003; Tavva et al., 2006). However, they have never become widely adopted in plants for several reasons.
In the case of ABA receptor-based systems, in planta reporter gene activation was shown only in response to azinphos-ethyl - a compound banned due to its extreme toxicity

1. These orthogonal gene expression circuits (Park et al, 2024) are indeed intended to report toxic molecules as biosensors for environmental monitoring. Such manuscript does not appear to suggest that azinphos-ethyl should be used for controlling traits in crops. Please update accordingly.

- Statistical analyses of several experiments are completely missing (Quantification of every fluorescence panel on Figure 4 and quantification and statistics of Figure 4E; Figure 5F and 5G). We added statistical analysis of Figures 5F and 5G, as requested (see below). For the tobacco leaf disc fluorescence images shown in Figure 4C, the original unmerged image files are sadly no longer available. As a result, we cannot confidently perform quantitative analysis without risking misrepresentation of the data. We therefore prefer to retain these results as qualitative, representative images.

2. It is not clear whether raw images from all replicates are unavailable and whether this situation aligns with the journal's guidelines during peer review.

- The measuring units at which the herbicides are applied change throughout the paper constantly (ppm, ppb, gr/Ha, etc). This is very confusing when trying to compare across experiments. Select one way of presenting in the main text and be consistent. Also, although the understanding of "billions" in the scientific literature is almost always 1000 millions, in general billions represent different things in different parts of the world (1 million of millions). It would be convenient to use a concentration unit that is understood across the world (molarity).
Thank you for pointing out the potential ambiguity of the term "billions." We have now converted all concentrations in the manuscript into mass concentrations. Converting these values into molar concentrations would result in quite awkward numbers, which we believe would confuse the readers rather than improve the clarity of the manuscript.

3. It should be easy to prepare a supplementary table listing the tested concentrations using molarity, which is commonly used in scientific publications. The authors should consider whether the journal follows this convention.

Version 2:

Reviewer comments:

Reviewer #2

(Remarks to the Author)

According to other reviewer reports and the initial manuscript the authors spent substantial improvement to the manuscript.

To clarify my former issue:
In Figure S1 the 7-chlortetracycline is shown.
The authors did the initial rounds of modelling with PDB entry 1DU7.
I suggest a suitable reference for the 7-chlortetracycline/TetR complex DOI: https://doi.org/10.1006/jmbi.1994.0138 with PDB entry 2TCT
by Caroline Kisker et al., The Complex formed between Tetracycline Repressor and Tetracycline-Mg2+ reveals Mechanism of Antibiotic Resistance. J. Mol. Biol. 247 (2), 260-280 (1995).

Using PDB entry 1DU7 is not helpful as this data are not accessible and misleading for the reader because of the entry title: Crystal structure of Tet repressor class D with 4-epi-tetracycline (no publication available).
This is a stereoisomer which would not bind to TetR due to steric clashes.
Therefore, this entry has been obsoleted and superseded by entry 2X9D, because the correct chemical structure of the bound ligand is iso-7-chlortetracycline.
Iso-tetracyclines are degradation products of tetracyclines, see DOI 10.1021/jm200332e with the PDB entry 2X9D titled: Tet repressor (class D) in complex with iso-7-chlortetracycline.

Minor point:
Figure 1 (A) Chemical structure of tetracycline (Tc).
This is correct, but for the modelling efforts 7-Chlortetracycline has been used.

--- end of comments ---

Reviewer #6

(Remarks to the Author)
The authors responded to the comments as requested.

Version 3:

Reviewer comments:

Reviewer #2

(Remarks to the Author)
After all ... o.k., it was a somewhat stressful, also for the authors I think, sorry.
Finally, my only comment refers to line 947 which has a pdf-format depending a strange character after "identical side-chains truncated to C "

--- end of comments ---

Open Access This Peer Review File is licensed under a Creative Commons Attribution 4.0 International License, which permits use, sharing, adaptation, distribution and reproduction in any medium or format, as long as you give appropriate credit to the original author(s) and the source, provide a link to the Creative Commons license, and indicate if changes were made.
In cases where reviewers are anonymous, credit should be given to 'Anonymous Referee' and the source.
The images or other third party material in this Peer Review File are included in the article's Creative Commons license, unless indicated otherwise in a credit line to the material. If material is not included in the article's Creative Commons license and your intended use is not permitted by statutory regulation or exceeds the permitted use, you will need to obtain permission directly from the copyright holder.
To view a copy of this license, visit https://creativecommons.org/licenses/by/4.0/

Referee #1 (Remarks to the Author):
Looger and colleagues present a new sulfonylurea herbicide responsive gene switch in soybean, maize, and rice. The herbicide gene switch is based on computationally inspired library generation for thifensulfuron-methyl (Ts), high throughput screening, and directed evolution of the tetracycline repressor (TetR). Using this approach, the authors identified multiple sensors with specificity to closely related herbicides ethametsulfuron-methyl (Es) and chlorsulfuron (Cs). Structures of sensors in the apo and holo states of two of these sensors showed the herbicides in the expected binding pocket on TetR, and for one sensor the apo- and DNA-bound structure showed the conformational changes expected for a bacterial allosteric transcription factor (aTF). The demonstrated Es-dependent induction of a reporter gene in soybean (Fig 1G), and maize leafs in vitro (Fig 3) but not in greenhouse-grown plants (lines 383-386). They demonstrate Wuschel2 and Baby boom expression induced by Es in maize and O. sativa (Fig 3). They also demonstrate herbicide-inducible gene control in Nicotiana and Arabidopsis. Most notably, they demonstrate an induction of Bt toxin in Nicotiana and show through an insecticidal assay that Es-induction saves leaf tissue from H. zea larvae (Fig 4).

With respect to the plant work, there were a few things which caught my eye (use of T1 lines for most of their data rather than T2 lines, a mention in the discussion that T2 lines were silenced (lines 613-614, data not shown), the lack of RNAseq measurements for determining whether there were transcriptional differences between Es +/- and WT plant lines; the lack of bulk phenotyping of plants like weight and height, etc). For these technical issues, I'll defer to other reviewers with expertise in plant sciences.

The primacy claim by the authors of no previous practical chemically inducible system in plants is confusing considering previous work by Sean Cutler's group on reengineering a native plant hormone receptor to respond to controlling agrochemicals (examples: Park et al Nature 2015; Park et al Nature Chemical Biology 2024). Perhaps there is an emphasis on 'crop plants' or 'practical' that precludes these papers; I think explicitly citing and explaining the authors' exclusion criteria is essential for a revision.

Thank you for the suggestions. We revised the introduction to add a more comprehensive review of past work, including work from the Cutler group, and to highlight the limitations of existing systems, which motivated the development of the system described in the manuscript.

Important features of a field-compatible system include low background, high magnitude of inducer-evoked response, and practicality of inducer application (*i.e.*, rapid penetration and movement to target tissues/cells, lack of degradation by target plant, neutrality to growth and development, safety, photostability, and registration for field use). Only two existing plant-specific induction systems employ approved agrochemicals as inducer: ecdysone receptor-based systems (Unger et al., 2002, Padidam et al., 2003; Tavva et al., 2006) and ABA receptor PYR1-based systems (Park et al. 2015; Park et al. 2024).

In the case of ABA receptor-based systems, *in planta* reporter gene activation was shown only in response to azinphos-ethyl - a compound banned due to its extreme toxicity. By contrast, the agriculturally relevant candidate from that paper - mandipropamid - was shown only to induce expression of RD29B, a native ABA-responsive gene, with no synthetic reporter gene induction demonstrated.

The ecdysone receptor (EcR)-based systems appeared highly promising in the early 2000s during the period of their active development (Unger et al., 2002, Padidam et al., 2003; Tavva et al., 2006). However, they have never become widely adopted in plants for several reasons.

The primary limitation is the poor bioavailability of approved ecdysone analogs (such as tebufenozide and methoxyfenozide) within plant tissue (Carlson et al., 2001), which makes induction inefficient, particularly when applied by spraying. In addition, many potent EcR agonists have been reported to occur naturally in plants (Adler & Grebenok, 1999) (*e.g.*, phytoecdysteroids like ecdysterone, a.k.a. 20-hydroxyecdysone), leading to undesirably high basal expression levels and diminished responses to exogenous inducer. These shortcomings have prevented EcR-based systems from being widely adopted for plant use. Thus, there appear to be no readily deployable agrochemical-inducible expression systems.

On the other hand, I do feel comfortable commenting on the technical aspects relating to the protein engineering & the biochemical assays. In large part, these aspects of the paper were performed well. The Supplementary Results (lines 7-333; Figs S13-34) describing the engineering campaign was interesting to read for a specialist. The impact of computational design on the development of the sensors appear minimal - positioning of the docked clusters (Fig S14) do not support the computational design algorithm as the solved structures with Es and Cs are distinct from any of the 20 clustered docked Ts used in the initial design process. The design process used Ts, but greater screening success was found with Es and Cs. The computationally designed library had ~10 positions encoded with NNK mutations, and hits in the first round of screening were quite sparse and with poor responsiveness. The replica plating screen is impressive for an academic lab in terms of scale. In terms of impact, there is not much to learn and/or new about the workflow or choice of targets. TetR is an example of a bacterial allosteric transcription factor (aTF). There is a voluminous literature on reengineering aTFs to respond to new chemicals. Examples include:

Tang, S. Y., & Cirino, P. C. (2011). Design and application of a mevalonate- responsive regulatory protein. Angewandte Chemie, 123(5), 1116-1118.
Taylor, N. D., Garruss, A. S., Moretti, R., Chan, S., Arbing, M. A., Cascio, D., ... & Raman, S. (2016). Engineering an allosteric transcription factor to respond to new ligands. Nature methods, 13(2), 177-183.
Wang, Z., Doshi, A., Chowdhury, R., Wang, Y., Maranas, C. D., & Cirino, P. C. (2020). Engineering sensitivity and specificity of AraC-based biosensors responsive to triacetic acid lactone and orsellinic acid. Protein Engineering, Design and Selection, 33, gzaa027.
Rondon, R., & Wilson, C. J. (2021). Engineering alternate ligand recognition in the PurR topology: A system of novel caffeine biosensing transcriptional antirepressors. ACS Synthetic Biology, 10(3), 552-565.
Additionally, there is an older paper which reports the redesign of TetR to respond to a different controlling ligand:
Scholz JMB 2003 https://doi.org/10.1016/S0022-(03)00427-3

Based on the above, the impact of this paper rests on the practicality and utility of this new Es-specific induction system in crop plants.

The Reviewer is correct that the reengineering of aTFs to respond to (very modestly) different ligands has been demonstrated before, and that the impact of our work rests largely on the utility of the developed SUR system in crop plants. We have revised the Introduction to include additional examples of prior aTF engineering efforts, including those cited above (see below). At the same time, we would like to emphasize that the leap in chemical space from tetracycline to SUs achieved in this work is far greater than in any of the listed examples. This in turn required far more dramatic protein redesign compared to the relatively small number of compensatory protein mutations in those studies. We believe that this combination of a massive

chemical-space transition and demonstrated utility in plants distinguishes the present study from earlier aTF engineering efforts, which are mainly proof-of-principle in nature.

Chemically-inducible expression tools are valuable academically for studying gene function and regulation, and practically for controlling phenotypes and processes in many organisms[1]. Numerous inducible gene expression systems have been discovered and developed through the generation of inducible chimeric transactivators and extensive protein-engineering efforts to expand the ligand specificities of the allosteric transcription factors (Tang & Cirino, 2011; Taylor et al., 2016; Wang et al., 2020; Rondon & Wilson, 2021). In plants, these efforts have resulted in the development of several widely used inducible systems, including dexamethasone- (Aoyama and Chua, 1997), estrogen- (Zuo, Niu, and Chua, 2000), and ethanol-inducible (Roslan et al., 2021) systems. These systems provide strong and temporally controllable gene activation in laboratory settings. However, their inducers are not suitable for field deployment due to regulatory restrictions (dexamethasone, estrogen) and challenges related to volatility, dosing control, and pleiotropic effects (ethanol).

For that, I don't have the appropriate expertise to weigh whether the evidence presented in different crop plants is sufficient to demonstrate a robust system. I have the following minor technical comments.

Minor comments

1. Given the proposed field use of crop plants, testing the specificity of the EsR(L15-20) sensor against a variety of registered agrochemicals would improve the robustness of the paper. Additionally, testing more than n=1 of chemicals for the sensors used in plants (Table 1) would improve the biochemical characterization for the paper.

While we agree that assessing potential cross-reactivity is important, we do not think that the probability of cross-reactivity with a chemically unrelated compound is high. Moreover, the number of registered agrochemicals (typically on the scale of hundreds, depending on country or region) makes comprehensive specificity testing impractical. If the Reviewer has specific agrochemicals in mind that they believe present a plausible risk of cross-reactivity, we would be happy to test those compounds.

2. Line 83. Reference 15, used in support of the interface design algorithm, should be removed. Although the ref 15 paper has not been retracted, follow-on literature (https://doi.org/10.1073/pnas.0907950106) did not support the conclusions of designed binders in that paper. Since the authors used a simple dock-design protocol, there must be another suitable reference for describing the computational protocol used.

We appreciate the concern that some of the experimental results from that paper have not been validated. The computational algorithms, however, are not suspect - and contrary to the Reviewer's claim, we would not classify the docking procedure as simple. We wrote that these (in so far as we are aware, never before used) algorithms were based on that work. There is no other appropriate reference to cite in that place. We will remove that statement entirely if the Reviewer and Editor insist.

3. Fig 1e & description of mammalian systems. This section reads like an afterthought and doesn't add much to the paper. From a biotech perspective, there are much better chemically inducible systems that work in mammalian cells & that are not herbicides. Given the validation of the engineered aTFs in bacterial systems & in planta, I suggest excising this section.

TetR-based inducible expression systems are by far most commonly used in mammalian cells and animals. Despite being herbicides, SUs are actually less toxic than Tc and related

antibiotics. They also show good bioavailability. Thus, these new inducer systems could be powerful tools in animal systems as well, as we show here. Frankly, we think that the animal applications are at least as important as the plant ones, despite the system being initially developed for plants. However, if the Reviewer and Editor feel strongly, we could move this to Supplementary.

The crystal structure of the class D TetR from *E. coli* in complex with iso-7-chlortetracycline (Protein Data Bank (PDB) ID 1DU7, **Figure S1A**) was used as the scaffold for design of TetR mutants capable of binding and being activated by SUs. Residues constituting a potential SU binding pocket were identified by inspection (**Methods, Figure S1B**). These 17 positions were mutated to all amino acids but cysteine and evaluated for compatibility with the TetR backbone, producing an initial amino acid library. In parallel, an atomic model of Ts was created with Chem3D (PerkinElmer), and a total of 144 conformers were generated, informed by available structures of SU herbicides in the PDB. Rotations of each conformer around its center of mass were placed at nodes of a cubic lattice centered at the center of mass of the iso-7-chlortetracycline in 1DU7, producing 2.3 x 10^9 total possible Ts placements in the binding pocket.

All these Ts placements were scored by assessing clashes with the scaffold and possible interactions with the side-chains of the residues from the previously created amino acid library at the 17 variable positions. Out of the billions of possible ligand placements, 1,811 were deemed possibly compatible with the protein scaffold. These ligand poses were quite diverse. We used hierarchical clustering to partition them into 20 clusters (**Figure S2A)**. The median pose was taken as representative of each cluster (**Figure S2B)**, and full sequence redesign (allowing all amino acids but cysteine) was again performed, this time in the context of each placed ligand. The amino acid diversity of top designs for each of the 20 poses was collated, and these 20 sets were merged into an amino acid library representative of all clusters (**Methods**). The resulting library at the 17 variable positions contained 4, 5, 4, 4, 5, 3, 8, 11, 10, 10, 8, 8, 7, 9, 6, 7, and 5 amino acids, respectively (**Figure S3**, column 1), yielding a theoretical library size of ~4 x 10^{13}.

Conversion of the computationally designed diversity into nucleic acid libraries for testing presented multiple practical hurdles. In most cases, the desired amino acid diversity at individual positions was not uniquely encodable by single degenerate codons - in most cases many codons would be required - increasing the number of oligos needed. This problem was further exacerbated by regions where multiple residues would need to be encoded on the same oligo due to their proximity (*e.g.*, positions 134, 135, 138, and 139), producing combinatorial complexity. Altogether, encoding all amino acids suggested by modeling - and no more - would require 1000s of oligos. To make the mutagenesis feasible, the library was expanded to encode a slightly enlarged set of amino acids at one position, and all 20 amino acids (using the degenerate NNK codon) at 10 other positions (**Figure S3**, column 2**, Supplementary_Oligos**), resulting in a much larger (~3 x 10^{19}) theoretical sequence diversity - but one that could be readily assembled in the lab.

4. The description of the computational design process and thoughts in library construction (lines 159-193) can be condensed significantly, particularly because it appears that design of the libraries did not help much.

We condensed the computational design description, as requested:

The crystal structure of the class D TetR from *E. coli* in complex with iso-7-chlortetracycline (Protein Data Bank (PDB) ID 1DU7, **Figure S1A**) was used as the scaffold for design of TetR mutants capable of binding to and being activated by SUs. Residues constituting a potential SU binding pocket were identified by inspection (**Figure S1B**). These 17 positions were mutated *in silico* to all amino acids but cysteine and evaluated for compatibility with the TetR backbone, producing an initial amino acid library. In parallel, atomic models of 144 Ts conformers were created and placed at nodes of a lattice centered at the center of mass of the iso-7-chlortetracycline in 1DU7 (**Methods**).

These Ts placements were scored by assessing clashes with the scaffold and possible interactions with the side-chains of the residues from the previously created amino acid library at the 17 variable positions. Out of all possible ligand placements, 1,811 were deemed possibly compatible with the protein scaffold and partitioned into 20 clusters (**Figure S2A)**. The median pose was taken as representative of each cluster (**Figure S2B)**, and full binding pocket sequence redesign was again performed, this time in the context of each placed ligand. The amino acid diversity of top designs was merged into an amino acid library representative of all clusters (**Methods**, **Figure S3**, column 1).

Conversion of the computationally designed diversity into nucleic acid libraries for testing presented multiple practical hurdles. In most cases, the desired amino acid diversity at individual positions was not uniquely encodable by single degenerate codons - in most cases many codons would be required - increasing the number of oligos needed. This problem was further exacerbated by regions where multiple residues would need to be encoded on the same oligo due to their proximity (*e.g.*, positions 134, 135, 138, and 139), producing combinatorial complexity. To make the mutagenesis feasible, the library was expanded to encode a slightly enlarged set of amino acids at some positions (**Figure S3**, column 2**, Supplementary_Oligos**), resulting in a much larger (~3 x 10^{19}) theoretical sequence diversity - but one that could be readily assembled in the lab.

5. Figure 2 would benefit from a key in the figure showing the different chemicals (Es, Cs, Tet)

Thank you for your suggestion. We added chemical structures of the compounds on panels A-C next to the appropriate repressor structures and modified the figure caption accordingly:

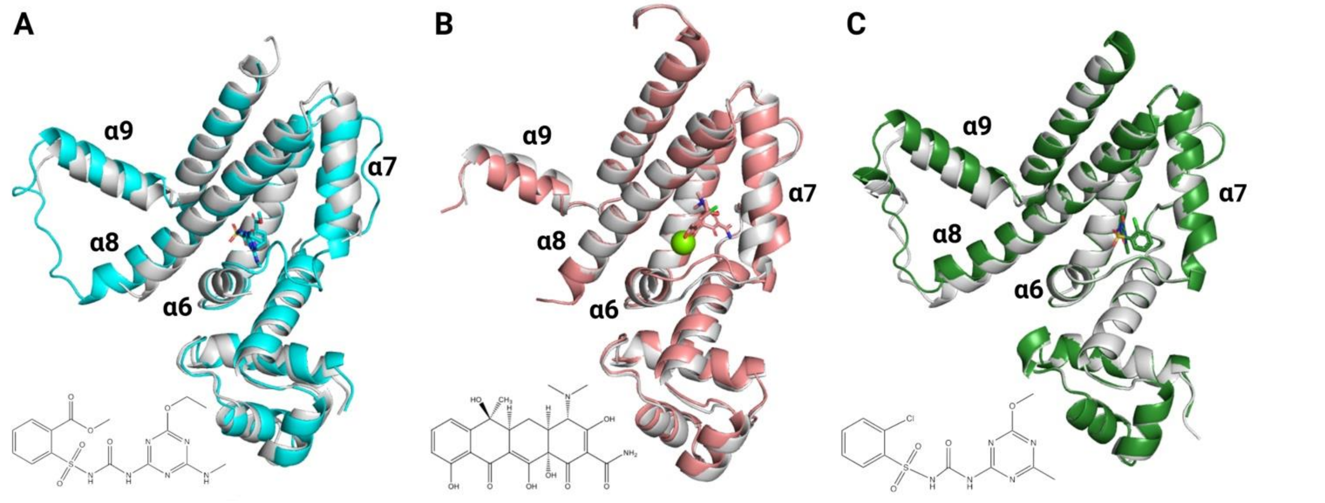

Figure 1.

Structural characterization of SURs. (**A-C**) Overlaid apo- (white) and ligand-bound (colored) monomers of (**A**) EsR (this study), (**B**) wtTetR (PDB IDs 1A6I and 2TCT), and (**C**) CsR (this study). Proteins are shown as cartoons, ligands are shown as sticks, and discussed helixes are labeled. Chemical structures of the corresponding ligands are shown at bottom left. (**D**) Overlaid class D wtTetR-Tc (PDB ID 2TRT), EsRL11-C06-Es, and CsRL4.2-20-Cs complexes: overall ligand position in the proteins and close-up of the ligand-binding cavity. Proteins are shown as cartoons, ligands are shown as sticks. Salmon – Tc, cyan – Es, green – Cs. (**E-F**) Details of protein-ligand interactions in the EsRL11-C06-Es (**E**) and CsRL4.2-20-Cs (**F**) complexes. Proteins are shown as white cartoons, ligands are shown as colored sticks, protein residues forming the ligand-binding cavity are shown as white sticks, key interacting residues are labeled, water molecules are shown as red spheres, and hydrogen bonds are shown as dashed yellow lines. (**G**) The relative orientation of the median cluster 9 Ts pose (gray) and Es (cyan) in the EsR-L11-C06-Es structure. (**H-I**) Overlay of the available (**H**) SulE-SU and (**I**) AHAS-SU complexes. Proteins are shown as white cartoons, ligands are shown as colored sticks.

6. Line 437 – the supporting table the text points to is a list of plasmids used. I was expecting a table of phenotype characteristics of plants from calli arising from each T0. Please explain.

Apologies for the misplaced Table reference. We have removed it.

7. Statistics. Significance of the effects, statistical method used, and confidence intervals should be reported in the main text (examples line 358 “800-fold”, :” Untreated fragments failed to demonstrate any growth, whereas Es-treated fragments regenerated full seedlings”).

In response to the Reviewer’s comment, we have revised the manuscript and added statistical analysis where possible. In the case of the examples that were explicitly listed:

1. “800-fold”: Because background-corrected control values are at or near zero, fold-induction metrics are not statistically robust. We therefore adjusted the manuscript to not report quantitative measurements.

Homozygous progeny of one of the clonal events were analyzed in herbicide soil soaking experiment. Soil soak with Muster® (active ingredient - Es) led to complete and widespread derepression, with DsRed-Express expression increasing from near-background levels in water-treated controls to strong signal in treated leaves (**Figure 1G, Figure S16**). This confirmed the feasibility of stable integration and functional SU-dependent derepression of SUR-controlled genes in intact plants.

2. “Untreated fragments failed to demonstrate any growth, whereas Es-treated fragments regenerated full seedlings”:

Untreated leaf fragments failed to demonstrate any growth (0/14), whereas 8/14 Es-treated leaf fragments regenerated full seedlings (Fisher’s exact test, $p = 0.0019$, **Figure 3E,F**).

Referee #2 (Remarks to the Author):

The manuscript of L.Looger represents a huge amount of sound work. The contents relies on tetracycline repressor variants trained to recognize not the native inducer tetracycline, but other artificial ligands. Such artificial-responsive gene switches with TetR modifications were already developed by the late Wolfgang Hillen (Erlangen, Germany) in various publications. In this light I can't see breaking news suitable for NATURE, even for possible application in agriculture.

The Reviewer is correct that the reengineering of the TetR to respond to new ligands has previously been demonstrated, most notably in the work of Wolfgang Hillen. However, we would like to emphasize that the leap in chemical space from tetracycline to SUs is far greater than from tetracycline to the compounds used in earlier studies (which differed in fairly minor

substituents on a conserved antibiotic core), and accommodation of this larger shift required far more extensive protein redesign than the limited set of compensatory mutations in the previous work. We believe that the combination of this unprecedented chemical-space transition (and correspondingly vast mutagenesis to the protein) and demonstrated utility of the system in plants clearly distinguishes the present study from any prior work in terms of both protein evolution methodology and biotechnological utility and supports its suitability for publication in Nature Communications*.*

Referee #3 (Remarks to the Author):

In this manuscript, authors evolve TetR into sulfonylurea herbicide binding transcriptional repressors (SUR) that could be used for selective induction of transcription in crop plants. The new SURs show binding specificity to different kinds of sulfonylurea herbicides in vitro and successfully show their ability to respond in different plant species and mammalian cells in culture. The work is technically strong and thorough, and the system is likely to be widely deployed by other labs.

In my opinion, the manuscript would be suitable for publication if greater emphasis is put on characterization of the system at the biological level in place of the structural characterization (that does not contribute with any major result).

Major criticisms:

- The novelty here is the demonstrated deployment of a genetic circuit that results in an engineered transcriptional response to a commonly applied chemical in crops in the field, rather than the specific effort to engineer a repressor that responds to a new ligand. While the system is deployed in multiple crops as proof-of-concept, much of the paper focuses on the molecular engineering instead of the in-planta characterization and biology. While the protein engineering component is well-executed, this aspect of the work - if reported alone - seems better suited for a more specialized audience. To highlight the broader significance and translation aspect of this system, I feel that more in depth characterization of the repressor function in planta, in crops, is critical to disclose in this initial report that is likely to be widely utilized by other labs. In at least one crop system, the authors should provide data that quantifies the following (and include a discussion of these issues in the main text):

1. Degree of orthogonality - two contexts are important: first, the response to endogenous molecules throughout the lifetime of the plant (rather than at a single timepoint). Second, does the repressor act on other sites in the genome. Comparative transcriptomics in an RNAseq experiment with multiple time points across the lifetime of the plant could indicate the degree of orthogonality. While it is likely that this repressor system is not completely insulated from native transcriptional regulation in the plant, these data would be critical for other labs who would like to deploy this tool and would be critical to report in the first disclosure of this system.

We would respectfully argue that investigating potential off-target effects of a novel TetR-based transcriptional regulator in plants is far beyond the scope of this work. TetR systems have been used in many plants, with no evidence of off-target effects. As we have preserved the DNA-binding domain in the present work, this likely extends to the repressors we demonstrate.

*We would also argue that our data showing undetectable DsRed-Express background signal across multiple stages of plant development (**Figure 5D**), together with the absence of pleiotropic effects in plants containing Es-inducible Wus2/Bbm expression cassette in the absence of SU (**Figure 3L**), demonstrates that there is no appreciable response of the*

repressors to endogenous molecules. Sulfonylureas are a synthetic class of molecules, with no known SU natural products. It is unlikely that there would either be endogenous SUs or that non-SU molecules would significantly activate the proteins, consistent with what we see in different plant systems.

2. In addition, it would be very helpful to characterize the dynamics of the response following treatment in different tissues of at least one example crop. For how long after herbicide application to understand the duration of the response following treatment

*We have presented multiple expression constructs/strategies in the manuscript and included the exact data on the dynamics of the response for some (**Figure 5C**). We are not sure how additional experiments (which expression strategy? which crop?) would help.*

- Statistical analyses of several experiments are completely missing (Quantification of every fluorescence panel on Figure 4 and quantification and statistics of Figure 4E; Figure 5F and 5G).

*We added statistical analysis of **Figures 5F** and **5G**, as requested (see below). For the tobacco leaf disc fluorescence images shown in **Figure 4C**, the original unmerged image files are sadly no longer available. As a result, we cannot confidently perform quantitative analysis without risking misrepresentation of the data. We therefore prefer to retain these results as qualitative, representative images.*

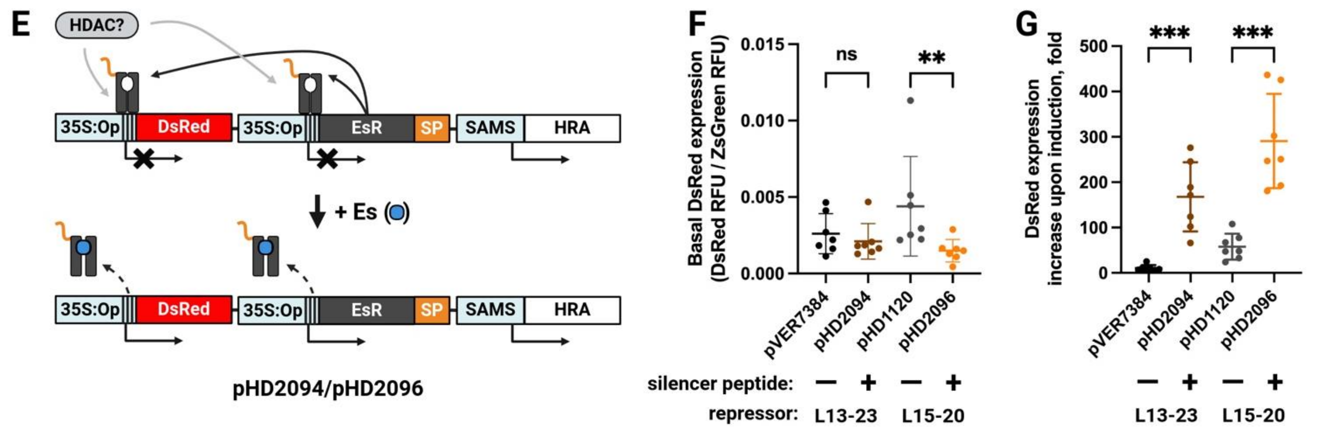

Figure 5.
<…> (**F, G**) Effect of added silencer peptide on two EsR repressors: EsR(L13-23) (vectors pVER7384 and pHD2094) and EsR(L15-20) (vectors pHD1120 and pHD2096). Background signal (**F**) and derepression magnitude (**G**) were assessed in tobacco leaves. The plots show n = 7 experimental replicates. Shown are mean and std. dev.; ns - not significant, ** - $P < 0.01$, *** - $P < 0.001$, two-tailed Mann-Whitney test; P-values are 0.535, 0.004, 0.0006, and 0.0006, respectively.

- In several sections of the manuscript, the expression levels of the SUR are directly linked to its ability to maintain the OFF state of the reporter, but also the requirement of SU to switch to the ON state. As an example, authors create an auto-repressible version of the SUR that allows for a stronger induction, but results in leakier expression of the reporter and a SUR version with a silencing inducer peptide. At no point do the authors try to circumvent this problem by a fairly straightforward experiment that would be replacing the promoter that drives the expression of the SUR. A better characterization of the simple construct architecture on Figure 1F would probably negate or downgrade the necessity of self-regulation.

We respectfully disagree with the Reviewer. We don't think that any constitutive promoter will be able to simultaneously enable low background signal (which requires high repressor concentration) and fast activation (which requires relatively low repressor concentration).

Minor criticisms:

- The measuring units at which the herbicides are applied change throughout the paper constantly (ppm, ppb, gr/Ha, etc). This is very confusing when trying to compare across experiments. Select one way of presenting in the main text and be consistent. Also, although the understanding of "billions" in the scientific literature is almost always 1000 millions, in general billions represent different things in different parts of the world (1 million of millions). It would be convenient to use a concentration unit that is understood across the world (molarity).

Thank you for pointing out the potential ambiguity of the term "billions." We have now converted all concentrations in the manuscript into mass concentrations. Converting these values into molar concentrations would result in quite awkward numbers, which we believe would confuse the readers rather than improve the clarity of the manuscript.

- In Figure 5A, B and C, Authors argue that the loss of de-repression is something that needs to be improved and they deploy a siRNA strategy for doing so. Although the result is a very impressive activation, I disagree with the authors negative interpretation of the loss of de-repression. The left panel on figure 5C shows what could be interpreted as a reversible system versus an irreversible system on the right panel. A reversible system would probably be of more interest to everyone than an irreversible system. If authors would make a better reversible system (higher and more uniform expression, sharper OFF), this manuscript would be even more impressive.

*Thank you for pointing this out. We have revised the title of **Figure 5** and associated manuscript text to highlight the usefulness of the temporary inducible system. Unfortunately, developing a better reversible system is out of scope of this study.*

Figure 2. Modifications to the SUR expression system.

Inducer applied a single time will eventually break down, resulting in re-repression of the target gene expression. While this can be beneficial for many applications, in some cases, longer activation might be desired. Moreover, induction propagation in large, complex tissues, in which penetration and distribution of small molecule inducers can be uneven (sometimes dramatically so), also presents a challenge. To achieve a more prolonged and penetrating induction, we added a short inhibitory RNA (siRNA)[48] targeting the repressor ($siRNA_{REP}$) under a repressor-regulated MMV-Op promoter, creating vector pHD1198 (**Figure 5B, Table S3**).

- Different SU resulted in different de-repression in Arabidopsis thaliana, even when the active principle was not the optimal ligand for the receptor. Authors discuss a bit about bioavailability, however they are not clear about the composition of the vehicle (surfactans, for example), solubility, polarity, etc. In fact, the experiment in maize where SUs are dissolved in DMSO proved better than other vehicles. Authors should use a comparable administration method, at least in one experiment, even though is understandable that the ultimate ligand would be provided as an off-the-shelf herbicide.

Given that out of all tested systems, we only see inducer behavior discrepancy in Arabidopsis*, we are more inclined to think that plant properties, rather than vehicle composition, are the reason for the observed phenomenon. This is further strengthened by the short genetic distance between* Arabidopsis *and plants known to metabolize the SUs; we really think that we understand this particular failure. We show it in the interest of transparency, and to show that*

the repressors still can be used robustly in the most common plant model system, just with other inducers.

- Receptors engineered to respond to herbicides and other commonly used chemicals to trigger plant responses in the field have been extensively developed. It would be helpful to reference and mention these prior engineering efforts of protein circuits (complementary to the transcriptional circuits reported here)

We have revised the Introduction to include a more comprehensive overview of existing inducible plant expression systems and to clarify how the system developed in this study addresses existing gaps.

Chemically-inducible expression tools are valuable academically for studying gene function and regulation, and practically for controlling phenotypes and processes in many organisms[1]. Numerous inducible gene expression systems have been discovered and developed through the generation of inducible chimeric transactivators and extensive protein-engineering efforts to expand the ligand specificities of the allosteric transcription factors (Tang & Cirino, 2011; Taylor et al., 2016; Wang et al., 2020; Rondon & Wilson, 2021). In plants, these efforts have resulted in the development of several widely used inducible systems, including dexamethasone- (Aoyama and Chua, 1997), estrogen- (Zuo, Niu, and Chua, 2000), and ethanol-inducible (Roslan et al., 2021) systems. These systems provide strong and temporally controllable gene activation in laboratory settings. However, their inducers are not suitable for field deployment due to regulatory restrictions (dexamethasone, estrogen) and challenges related to volatility, dosing control, and pleiotropic effects (ethanol).

Important features of a field-compatible system include low background, high magnitude of inducer-evoked response, and practicality of inducer application (*i.e.*, rapid penetration and movement to target tissues/cells, lack of degradation by target plant, neutrality to growth and development, safety, photostability, and registration for field use). Only two existing plant-specific induction systems employ approved agrochemicals as inducer: ecdysone receptor-based systems (Unger et al., 2002, Padidam et al., 2003; Tavva et al., 2006) and ABA receptor PYR1-based systems (Park et al. 2015; Park et al. 2024).

In the case of ABA receptor-based systems, *in planta* reporter gene activation was shown only in response to azinphos-ethyl - a compound banned due to its extreme toxicity. By contrast, the agriculturally relevant candidate from that paper - mandipropamid - was shown only to induce expression of RD29B, a native ABA-responsive gene, with no synthetic reporter gene induction demonstrated.

The ecdysone receptor (EcR)-based systems appeared highly promising in the early 2000s during the period of their active development (Unger et al., 2002, Padidam et al., 2003; Tavva et al., 2006). However, they have never become widely adopted in plants for several reasons. The primary limitation is the poor bioavailability of approved ecdysone analogs (such as tebufenozide and methoxyfenozide) within plant tissue (Carlson et al., 2001), which makes induction inefficient, particularly when applied by spraying. In addition, many potent EcR agonists have been reported to occur naturally in plants (Adler & Grebenok, 1999) (*e.g.*, phytoecdysteroids like ecdysterone, a.k.a. 20-hydroxyecdysone), leading to undesirably high basal expression levels and diminished responses to exogenous inducer. These shortcomings have prevented EcR-based systems from being widely adopted for plant use. Thus, there appear to be no readily deployable agrochemical-inducible expression systems.

- While the writing is very clear and easy to follow, in several instances, the authors could also use more objective, specific, and quantitative language. A few examples:

We have corrected the language to be more specific:

- update “prudent” – suggested replace with recommended? Practical? Feasible?
Tetracycline repressor (TetR)-based gene switches work in plants, but antibiotics are neither approved nor advisable for crop use.

- “intricate interactions” – unclear what is meant by intricate for a small molecule protein interaction
Crystal structures of both EsR and CsR repressors in complex with their respective SU ligands revealed extensive interactions explaining their high-affinity binding.

- “use agrochemicals has not been attempted” – suggested replace with “has not previously been reported”
This part of the introduction has been significantly rewritten, and this language is not present anymore.

- “spread of the response” – does this relate to movement of the inducer chemical herbicide? Or some other aspect of transcriptional regulation?
Addition of a repressor-regulated siRNA targeting the repressor transcript increased the magnitude and spatial distribution of the response following herbicide treatment and resulted in a partially bi-stable gene switch.

- Intro: “no practical” – however, engineered transcriptional regulation is available in plants for research purposes – just not suitable for deployment in the field. Perhaps be more specific?
This part of the introduction has been significantly rewritten, and this language is not present anymore.

Other general comments:

Line 123 – KM3 design. How many mutants on LacI or binding site that restore KanR in the absence of TetR (escapes) were detected?

We did not quantify such escapes.

Line 175-176 Replace “full sequence redesign” with line from materials and methods (line 742-743) “full binding pocket redesign” for clarity. If that is not correct, please be more explicit on what this means.

Thank you for pointing this out. We added “binding pocket” to clarify the sentence as suggested:

*The median pose was taken as representative of each cluster (**Figure S2B)**, and full binding pocket sequence redesign was again performed, this time in the context of each placed ligand.*

Figure S3. For clarity, select one of the positions and label each one of the columns to make it easier to understand. Col1=computationally designed library=CD; Col2= primer-encoded library=P; Col3=randomly picked clones=R; Col4=selected hits=H. To better explain the problem with primer design, take a representative of a simple clone and a complex clone and create a diagram. This diagram can show the closeness of the residues to be mutated and the enlarged diversity if specific clones are to be used. For example: present the DNA sequence of the template highlighting the positions to be mutated, underscore the codon to be mutated and list the possible codons to mutate to for the simple type. For the complex type, list some example codons and the number of possible codons that should be placed in that position, showing the result of the multiplication of all the possibilities. Do randomly picked clones represent the 20k clones (Line 198)? Are selected hits the chosen 40 (Line 201)?

*Thank you for your suggestions. We added column labels to **Figure S3** and clarified the number of sequenced random clones in the manuscript text (see below). However, we feel that the current text already explains the challenges of encoding only the computationally designed amino acid diversity with sufficient clarity, and that the proposed diagram would not substantially improve reader understanding, particularly given that this information is not critical to the manuscript.*

This library, denoted L1, was assembled by PCR, cloned into the pVER7314 vector, and transformed into the KM3 *E. coli* strain. The library was first plated onto Luria Broth (LB) plates containing carbenicillin (to select transformants) and kanamycin (to select for intact repressor activity). After sequencing ~100 random colonies to verify library quality and diversity (**Figure S3**, column 3), ~20,000 randomly picked clones were screened for Ts-induced derepression and preserved repressor activity by replica-plating onto X-gal-containing minimal media (M9) agar plates with or without 20 µg/ml Ts.

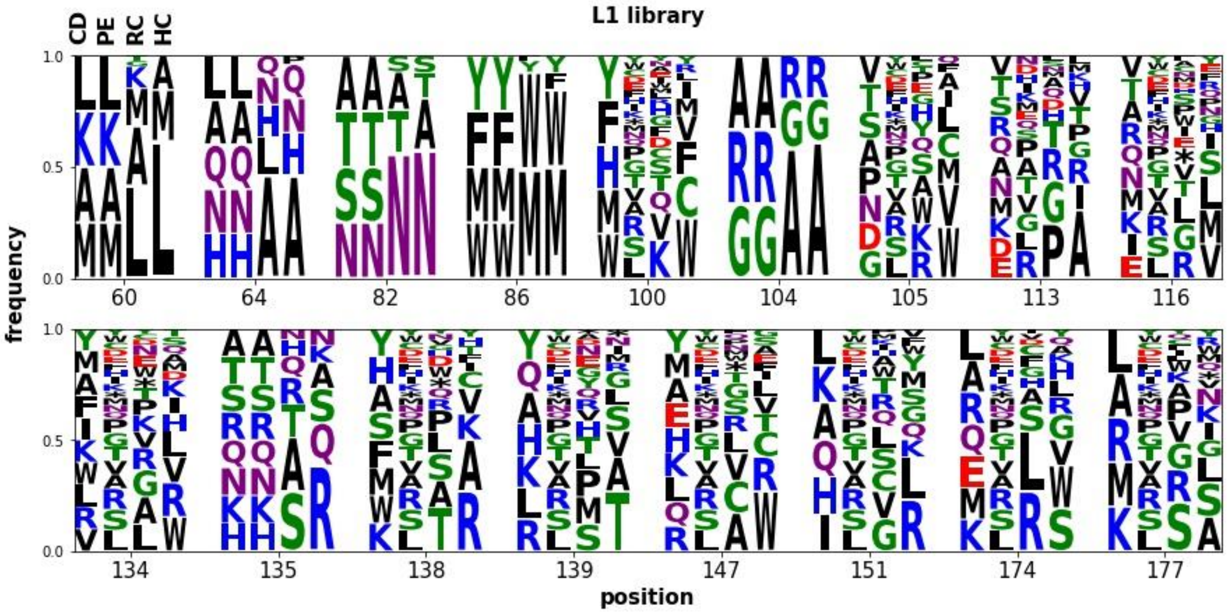

Figure S3. Round 1 library design, construction, and screening results. For each of the 17 mutated positions, shown are amino acid frequencies of the computationally designed library (column 1, "CD"), primer-encoded library (column 2, "PE"), randomly picked clones (column 3, "RC"), and selected hits (column 4, "HC").

Line 196-198. How many colonies were sequenced to verify library quality and diversity? How many total colonies were obtained from the transformation of the library? What is the reduction of diversity in the library? If sequencing was done by NGS, please provide an estimation of diversity reduction from expected ~3x10^9 to actual diversity. If not possible, please provide justification.

On average, we sequenced around 100 random colonies from each library for quality control. When this project was started (2005), NGS had very limited availability and was extremely expensive, so the assessment was based on limited Sanger sequencing and was mostly qualitative rather than quantitative. There is not enough data to calculate the reduction of diversity, and there are no samples left to re-analyze. We added information about the number of sequenced colonies to the manuscript:

After sequencing ~100 random colonies to verify library quality and diversity (**Figure S3**, column 3), ~20,000 randomly picked clones were screened for Ts-induced derepression and preserved

repressor activity by replica-plating onto X-gal-containing minimal media (M9) agar plates with or without 20 µg/ml Ts.

Line 198. How were the 20k colonies selected? Random?

Yes, 20k colonies were selected randomly. We now clarify this in the manuscript:

After sequencing ~100 random colonies to verify library quality and diversity (**Figure S3**, column 3), ~20,000 randomly picked clones were screened for Ts-induced derepression and preserved repressor activity by replica-plating onto X-gal-containing minimal media (M9) agar plates with or without 20 µg/ml Ts.

Figure S4. Provide chemical structures of SU tested. Show fold increase without vs with SU/Atc inside each box of the table. Explain divergence of A (beta-gal) vs B (MUG) responses for selected clones. What's the hypothesis for the mutTetR responding to some but not all Sus?

Thank you for your suggestions. We have added numbers showing fold increase in β-gal activity in the presence of each compound vs. *no-ligand control to subplots A and B and it really improved the readability of the data. We also added a third subplot (C) to the figure showing the chemical structures of the tested SU compounds.*

We believe that the difference between results shown in subplots A and B is primarily driven not by the compound used (X-gal vs. MUG), but by the assay type (colony color vs. β-gal activity of lysate). The former is higher-throughput but less sensitive and more prone to variability.

While SUs have a common substructure, they have different substitutes that define the molecule size and chemical properties. Given the depth of the TetR ligand-binding pocket, these differences are substantial enough for mutTetRs to be able to accommodate some but not all SUs.

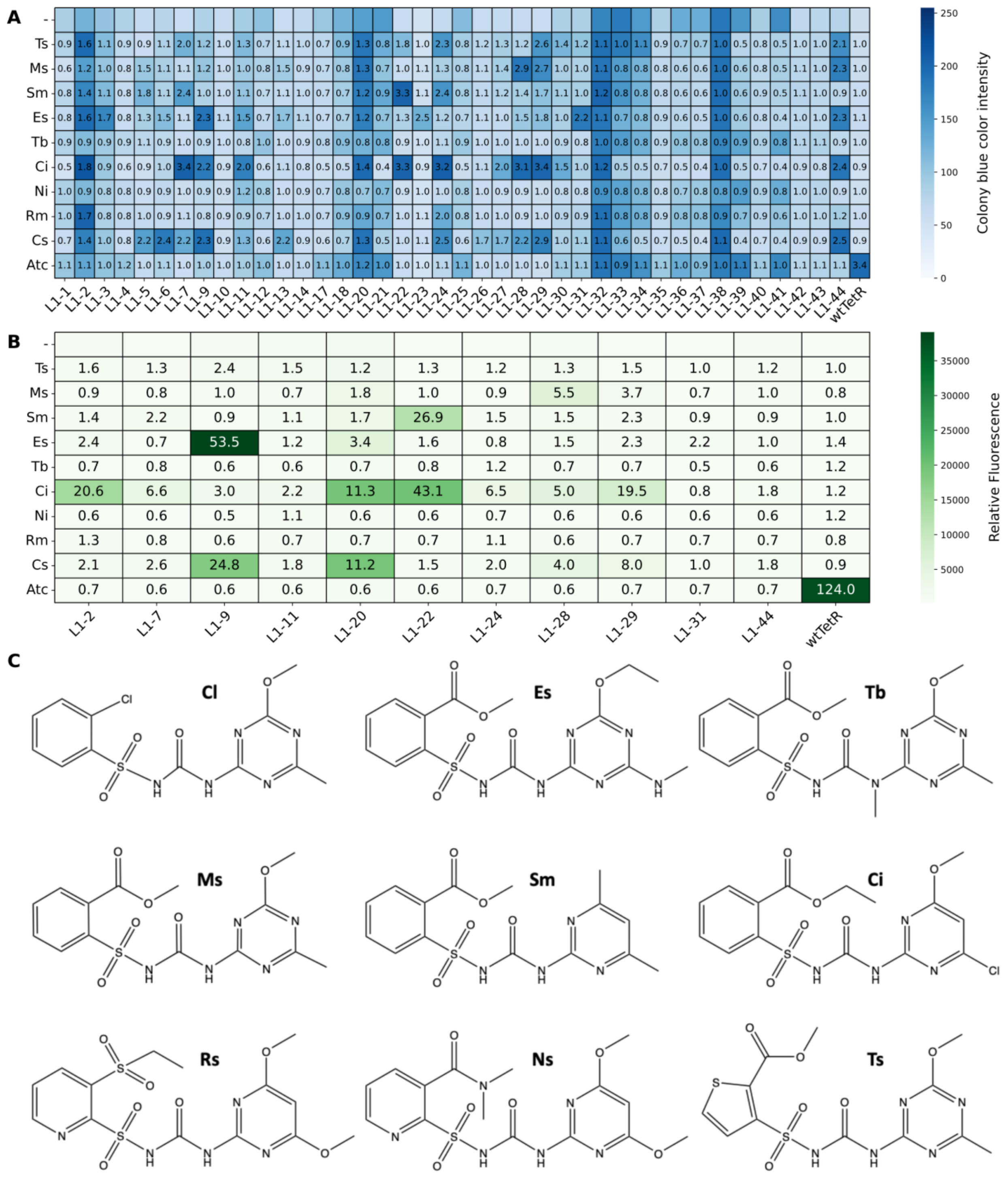

Figure S1. Derepression of 1st round hits by nine SUs and Atc. (**A**,**B**) β-gal activity of KM3 cells expressing an L1 library hit or wild-type TetR assessed (**A**) based on intensity of the blue color of the colonies after 60 hours of incubation on plates or (**B**) in bacterial lysate using fluorogenic substrate 4-methylumbelliferyl-β-D-galactopyranoside (MUG) after 2 hours of incubation with chlorsulfuron (Cs), rimsulfuron (Rs), nicosulfuron (Ns), chlorimuron-ethyl (Ci), tribenuron-methyl (Tb), ethametsulfuron-methyl (Es), sulfometuron-methyl (Sm), metsulfuron-methyl (Ms), thifensulfuron-methyl (Ts) (all at 10 ppm (~25 µM)), anhydrotetracycline (atc) at 4 µM, or without any ligand (-). Numbers in cells represent the ratio of β-gal activity in the presence of the compound vs. in the absence of any ligand. (**C**) Chemical structures of the SU compounds used in screening.

Table 1. Elaborate on specificity of responses vs broad responses to SU.

While our screening pipeline did not explicitly include negative selection steps, as strict ligand selectivity was not a requirement at this stage of the project, subsequent characterization revealed that some of the top variants for a given SU can also be induced by other SUs. However, we are confident that improved selectivity should be achievable, if desired. For example, EsR(L13-9) already shows significant cross-reactivity with only one additional tested SU and exhibits >1,000-fold higher sensitivity to Es compared to the other tested SUs.

Solving the structures of both the ligand-bound and apo versions of SURs (although of slightly different mutants in the case of EsR) allowed us to investigate differences between the DNA-bound and non-DNA-bound forms. Alignment of apo EsR-L7-D01 with Es-bound EsR-L11-C06 showed structural perturbations similar to those seen upon Tc binding to wtTetR[20]. The most significant conformational changes include unfolding of the C-terminus of α6, perturbation of the loop connecting helix α6 to α7 (although a part of this loop was unresolved in EsR-L7-D01), and displacement of α7, α9, and the N-terminus of α8 (**Figure 2A**), suggesting a mechanism of ligand-induced derepression analogous to wild-type (**Figure 2B**). Interestingly, the apo and Cs-bound structures of CsR CsL4.2-20 overlap almost perfectly, except for the N-terminus of α8, α9, and the loop connecting them (**Figure 2C**). More studies would be needed to determine whether the Cs-mediated derepression indeed utilizes a different mechanism, or whether this is a crystallization artifact.

Lines 240-252: Is it fair to compare structures of different mutants as if they were the same structure in the apo or holo form? Are the changes sufficient to justify anything? Are they needed in the bulk of the paper? I would probably move this paragraph and what they are trying to say to supplemental.

While obviously not ideal, comparison of the structures of different mutants is a fairly common practice in structural biology, as it is not always possible to get a structure of the most desired mutant. We believe that the structural information will be found interesting and important by readers interested in the protein engineering aspects of the work, but we can move this part to Supplementary if it is deemed not significant enough.

Validation of SUR function in mammalian cells

We first tested the new SURs using transient expression in mammalian cells. We cloned two EsR constructs (L13-9 and L15-20) and two CsR constructs (CsL4.2-15 and CsL4.2-20) in place of TetR in the pcDNA6/TR vector of the T-REx kit (Thermo-Fisher), and superfolder GFP (sfGFP) into the pcDNA4/TO reporter vector (**Figure 1D**). The pcDNA6/Repressor (one of the two EsRs or two CsRs, or the TetR parental vector) and pcDNA4/TO-sfGFP vectors were co-transfected into HEK293 cells. Tc, Es, Cs, or DMSO (carrier solution - negative control) were added to the cells the next day. The cells were allowed to express for 24 hours after induction, then imaged (**Figure 1E**) and quantified for green fluorescence by flow cytometry (**Figure S15**). TetR responded strongly to Tc but not to either SU ligand; EsR-L13-9 and L15-20 responded strongly to Es, almost negligibly to Cs, but not to Tc; CsRL4.2-15 responded strongly to Cs, weakly to Es, but not Tc; CsRL4.2-20 responded strongly to both Es and Cs, but not Tc. Without repressor, the sfGFP signal was equivalent in all cells. Thus, the designed repressors function as intended in mammalian cells, the SU compounds are cell-permeable and do not show any obvious toxicitv at the used concentrations.

Lines 320-335. It is strange to see a reference to figure 1 at this point of the paper. Why are the cloned constructs not the same used for crystallography? Elaborate about the differences of specificities in bacteria (Table 1) vs mammalian cells. It seems that there is no overarching rule on when there will be cross-induction for the herbicides when used in mammalian cells compared to the EC50 values on Table 1.

*The cloned constructs were selected based on protein performance and not on our ability to crystallize them, which would be a strange selection criterion given that protein crystallization success is difficult to predict and that better-performing proteins (for example, more soluble) might be harder to crystallize. We also respectfully disagree with the statement that there is no correlation between cross-induction observed in mammalian cells and EC_{50} values in **Table 1** – we think they align quite well, in fact. For example, EsR-L13-9 shows the highest selectivity in mammalian cells (**Figure S15**) and has the lowest Es EC_{50} and highest Cs EC_{50} when tested in bacteria (**Table 1**), while the least selective in mammalian experiment CsL4.2-20 has the lowest EC_{50} for the non-target inducer (Es) among all repressors tested in mammalian cells.*

Lines 383-386. State how old the treated maize plants were in this experiment. What is the EC50 for the herbicide as such in maize in the field/greenhouse versus the concentration required for binding to the repressor? Is it possible that not enough Es for repressor is making into the plant? What about drench experiments instead of foliar application? Later, drench experiments are done in other plant species, why not done in maize in the same way?

The plants used for the maize spray experiment were grown to the V6 stage; we have added this information to the manuscript (see below).

A maize EC_{50} for Es is not readily available in the literature as herbicides are typically characterized by the application rate needed for weed control rather than by a crop EC_{50} (the crop should be tolerant at labeled rates). The typical Es application concentration is 200 mg/l, which is >15,000-fold higher than the measured EC_{50} of EsR(L15–20) (12 µg/l). Thus, the gene switch is activated at concentrations far below those applied in the experiment and in typical crop application.

The observation of induction after vacuum-infiltration and at the application site of the DMSO formulation is consistent with uptake and successful induction in treated tissue. However, limited movement of Es within maize tissue remains a plausible explanation of the lack of systemic induction, since we did not observe induction spreading beyond the treated area. We also performed drench experiments in maize, but these likewise did not produce induction.

Interestingly, although robust reporter activation was observed in maize *in vitro,* we were unable to induce expression in V6 stage greenhouse-grown plants using a conventional herbicide spray test or soil drench. We could only induce expression in mature plant leaves by applying Es dissolved in DMSO, but expression was limited to the application area.

Line 449-459. Explain why putting the repressor under the control of the 35S:Op would result in higher induction upon addition of Es. It is hard to explain that having released expression of the repressor with Es would result in higher expression levels. Is it possible that the promoter for driving expression of EsR is too strong and more Es or more binding sites in the genome are needed to release the competition? Do you even need expression of EsR in the second part of the construct in B. Maybe just by adding competing Operators in any part of the T-DNA would be sufficient to tune down the repression.

Placing the repressor under the control of 35S:Op results in higher sensitivity and response to Es because it tailors the number of repressor copies produced in each particular cell through inhibition of its own expression. The result of this is that less Es is needed to shift the equilibrium to allow expression of the reporter gene. Using a weaker constitutive promoter to drive EsR expression could also increase sensitivity, but this approach comes with several drawbacks. Most importantly, use of a weak promoter driving repressor expression means that some cells will have very low repressor expression (given normal levels of cell-to-cell variability), and in these cells it is likely that a healthy amount of reporter gene would be expressed before the repressor could manage to repress it. Moreover, such an approach would require labor-intensive promoter strength optimization for each specific case. In contrast, the autoregulated promoter approach enables default use of a strong promoter without any associated loss of sensitivity. We updated the text to make the rationale for placing the repressor under the control of the 35S:Op promoter clearer:

After demonstrating the new repressors' general usability in multiple plant systems, we focused on making improvements to the expression system to increase responsivity to ligand. For this we decided to use a negative autoregulation approach whereby the repressor regulates its own expression level through operator sequences incorporated into the promoter driving its transcription. This ensures optimal repressor concentration level that requires less inducer to saturate the pool of repressor molecules, cause operator derepression, and induce expression of the reporter gene. This method has been shown to improve response amplitude and kinetics in theory[41] and experimentally in *E. coli*[42] and yeast[43], the latter application employing TetR. To test this approach in plants, we created two vectors with the *35S-Op:DsRed-Express* fluorescent reporter, constitutively expressing HRA, and either a constitutive (pVER7385, **Figure4A, Table S3**) or autoregulated (pVER7384, **Figure4B, Table S3**) EsR(L13-23) repressor.

We then tested the effect of SUR autoregulation in intact plants. Stable transformants from both constructs were treated with Muster® at the registered application level (20 g Es/Ha) through leaf spraying or base watering. Control plants were left untreated. Plants with the autoregulated repressor (pVER7384) demonstrated dramatically increased reporter gene activation in both treatment modalities (**Figure 4D**). Untreated pVER7385 plants were dark; control pVER7384 plants showed very dim, but non-zero, fluorescence (**Figure 4D**, bottom row). Thus, the negative autoregulation strategy indeed increased the system's sensitivity to the ligand but at a cost of slightly increased leakiness.

Line 460-467. Support for the hypothesis mentioned above. It is possible that the expression of EsR has to be tuned down. What is the difference in treatment with maize?

We agree that the expression of EsR had to be tuned down, that is why we placed it under the autoregulating promoter.

To test the SURs in *Arabidopsis* we used the same pVER7384 vector (**Figure 4B, Table S3**) containing autoregulated repressor. Homozygous T2 seedlings of inducible *A. thaliana* transformants with a single-copy insertion were sprayed at the rosette stage with Ally® (Ms), Classic® (Ci), Glean® (Cs), or Muster® (Es) at 0.25x application level (5 g/Ha). A null segregant treated with Classic® was used as a control. Interestingly, Ally®, Classic®, and Glean®, but not Muster® (which contains the best ligand for the used EsR(L13-23) repressor), induced widespread DsRed-Express expression within 3 days of application (**Figure S17**), similar to Muster®-sprayed tobacco (**Figure 4D**, top row, right column).

Lines 473-480. Is it possible that the differences between herbicides is not due to their chemical nature but instead to the surfactant that each herbicide has? Is it possible that the different chemicals have different bioavailabilities that compensate for their specificity? This experiment should have been done with each chemical delivered in the same solvent.

Given that out of all tested systems, we only see inducer behavior discrepancy in Arabidopsis*, we are more inclined to think that the plant properties, rather than the composition of the vehicle, are the reason for the observed phenomenon. This is further strengthened by the low genetic distance between* Arabidopsis *and plants known to metabolize the SUs utilized; we really think that we understand this particular failure.*

Lines 489-503. How many times was the experiment repeated? Where is the quantification and statistical analysis?

*The experiment was performed once using three independent lines for each construct and four technical replicas per line. We have added this information to the manuscript. We do not have a robust way to quantify these data for statistical analysis, but we believe the representative images shown on **Figure 4E** clearly illustrate the results.*

Single-copy transformants with inducible AcGFP expression were then identified for both lines. Selected single-copy events (three events for each line, four technical replicas for each event) were tested for insect resistance by introducing 2nd instar *H. zea* larvae to leaf disks on agar with or without 100 ng/ml Es for 48 hours.

Lines 504-544. Deploying an siRNA to downregulate EsR upon ES treatment is a great way to release expression of the repressed genes as shown in the manuscript, however, it could impact reversibility of the system as shown in figure 5C.

That is correct. We have tested and shown different expression strategies to provide the readers with examples of induction regimens that can be achieved with the engineered SURs. It is up to each researcher to pick the one that is the most suitable for their purpose, given their system, question, etc. Furthermore, the temporal properties of the system can be tuned by adjusting the strength of the promoters driving expression of the repressor and the siRNA. For example, higher levels of repressor transcripts would be harder to override with siRNA, resulting in a faster reset of the system.

> While the reduction of the number of *TetO* sequences to one in the SUR promoter of the autoregulated repressor system seemed to reduce the basal DsRed-Express expression compared to vector pVER7384 with three *TetO* sequences (**Figure 5D,** top; **Figure 4D**, bottom row), it should also reduce the system's sensitivity to the ligand. As an alternative way to reduce basal reporter gene expression, we tested the fusion of a small transcriptional silencer peptide element from *Arabidopsis* transcription factor AtMYBL2 responsible for transcriptional silencing of anthocyanin biosynthesis[52] to the C-terminus of the repressor.

Lines 546-548. Revise figure number reference. Figure 5D? It cannot be ruled out that the effect of a tighter control of the reporter and the response to Es is not related to controlling the basal expression of EsR.

We believe that the figure number reference is correct. We do not claim that we have fully ruled out all possible reasons for the observed phenotypes, but we definitely think that the basal level of the repressor expression plays a key role. We would appreciate it if the Reviewer can explain in more details why they think the Figure 5D is misreferenced and suggest modification to the sentence that would make it less controversial.

Lines 558-566. Missing statistics to claim about Figure 5F and 5G. While the claim is obvious for G, not so much for F.

We added statistical analysis and modified the associated figure caption and text accordingly.

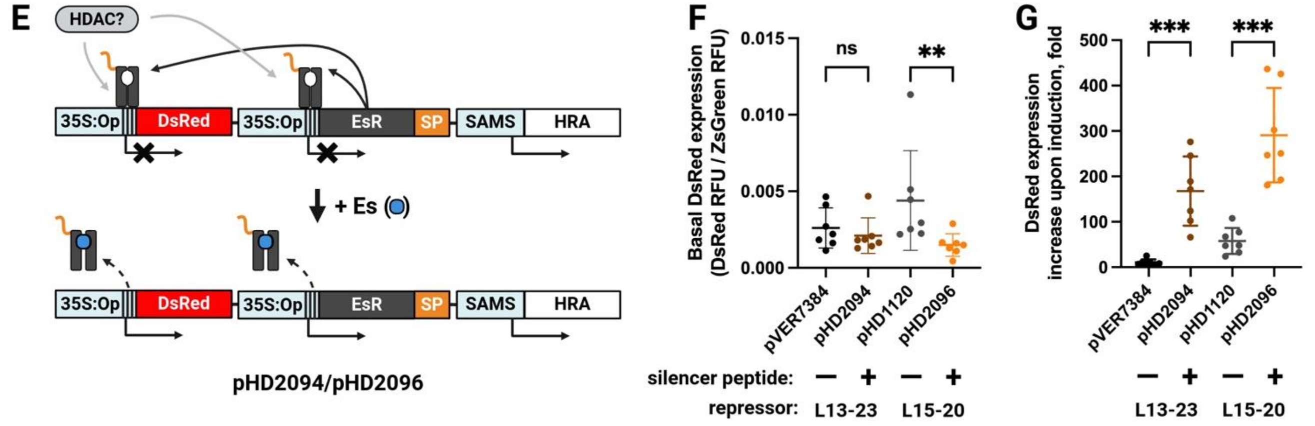

Figure 5.
<…> (**F, G**) Effect of added silencer peptide on two EsR repressors: EsR(L13-23) (vectors pVER7384 and pHD2094) and EsR(L15-20) (vectors pHD1120 and pHD2096). Background signal (**F**) and derepression magnitude (**G**) were assessed in tobacco leaves. The plots show n = 7 experimental replicates. Shown are mean

and std. dev.; ns - not significant, ** - P < 0.01, *** - P < 0.001, two-tailed Mann-Whitney test; P-values are 0.535, 0.004, 0.0006, and 0.0006, respectively.

EsR(L13-23) or EsR(L15-20), in plasmids pVER7384 or pHD1120 respectively, were extended with a 6-amino acid transcriptional silencer peptide (TLLLFR). The resulting vectors, pHD2094 and pHD2096 (**Figure 5E, Table S3**), respectively, were tested for basal expression and derepression alongside control vectors pVER7384 and pHD1120 using a rapid *Agrobacterium*-based *N. benthamiana* leaf infiltration assay. Interestingly, only the construct encoding EsR(L15-20) with the silencer peptide motif showed decreased baseline expression (**Figure 5F**) but both showed 6-10-fold increased derepression (**Figure 5Figure *2*G**). The latter might be explained by more stringent autorepression, which reduces the total concentration of repressor that needs to be titrated by the added ligand.

Lines 565-566. "[...] which reduces the total concentration of repressor that needs to be titrated by the added ligand." I couldn't agree more. It highlights the importance of controlling for the levels of expression of the repressor. As suggested many times above.

Referee #5 (Remarks to the Author):

The manuscript by McBride et al. describes the development of an herbicide responsive synthetic gene circuit for chemically inducible gene expression. The circuit utilizes the TetR system, which natively encodes a TetR repressor that binds to an operator sequence and inhibits gene expression of the associated locus through steric hindrance. The authors utilized an E. coli-based system to select for mutated receptors that can respond to chemicals unrelated to the tetracycline antibiotic—specifically sulfonylurea-type herbicides, which are widely used in agriculture. The most successful candidate identified from the screen (EsR, an Es-binding repressor) exhibits low nanomolar sensitivity to Es when utilized in a gene circuit to regulate fluorescent protein expression. The authors also incorporated two types of negative feedback loops, which increase the sensitivity of the signaling system: an autoregulatory loop where the EsR binds to its own promoter to inhibit expression and a siRNA-based silencing system that further reduces EsR transcript levels.

Implementation of the EsR-based system demonstrated efficacy in several agriculturally relevant contexts including: tissue-culture-based transformation of maize using Es inducible morphogenetic regulators (WUS/BBM), inducible expression of the Bt toxin gene for insect resistance, and induction of persistent expression in whole tobacco plants. Different aspects of the system were demonstrated in maize, rice, tobacco and Arabidopsis as well as human cell culture.

The work represents an important addition to the genetic engineering toolkit for plants and crops. As the authors state, being able to conditionally regulate the activity of a pathway in crops may improve the implementation of biotechnological solutions as it would enable induction of traits on an "as-needed" basis, potentially limiting the negative effects of constitutively expressing crop protection systems. However, demonstration that the system the authors developed provides such an advantage has not been made. Important questions such a field efficacy and deployability require extensive further study. I also have substantial concerns that the system described is unlikely to be widely implemented in agriculture as it would require farmers to utilize herbicides even in situations where weed control is not the goal since the inducer is an herbicide. The authors would need to demonstrate several advantages of their system under field conditions to be convincing as an agricultural innovation. Otherwise,

it primarily seems more like a tool best suited for academic research, which is valuable, but more limited in impact.

Major comments:

1) The authors highlight that the TetR system is unwise to utilize for crops as widespread application of antibiotics (tetracycline) could have environmental and human health impacts. However, why is the widespread use of an herbicide not also a concern? Considering that the SUR system would lead farmers to apply herbicide even when weed control is not necessarily desired seems concerning.

We agree with the Reviewer that excessive or unnecessary herbicide use is an important concern. However, the focus of our research is not to encourage additional herbicide application, but rather to demonstrate that gene switches can be built around compounds already approved, widely used, and considered safe for agricultural deployment, in contrast to tetracycline and related antibiotics, which are categorically unsuitable for field use. Our manuscript establishes the feasibility of large shifts in substrate specificity, which we expect will ultimately enable the development of switches responsive to even safer, more selective, or non-herbicidal inducers in future iterations. As to the specific point raised, the SUs that our system is using for transgene induction are agrochemicals that are often being added to fields of common crops anyway to control weeds - this was a main criterion for their selection.

What is the stability of these herbicides in the environment? Would herbicide residue from previous seasons, or drift into a field from application in neighboring plots, not lead to unintended induction of the system? The authors need to test the utility of their system under field conditions and utilize a construct design that provides a field-relevant trait of value.

SU herbicides have been used for decades now, and information about their year-to-year environmental persistence is available (both on product labels and in independent studies (e.g., doi: 10.1016/j.chemosphere.2005.01.051, 10.1071/EA04221)). While we agree that testing of the system under field conditions will be required before the system can be widely implemented, the proposed multi-year field study is out of scope of this manuscript.

2) The design and optimization of the transgenes used in the study is not well described. How was the optimal architecture of EsR promoters achieved for the regulation of downstream outputs? Figure 3D, construct PHP74334 appears to have different number of EsR binding sites in the promoter of WUS2 compared to BBM. What is the basis of this different design? To understand whether the presented constructs reflect optimal designs, the authors should present this tuning as part of a more rigorously described synthetic biology approach.

The objective of this work was to demonstrate the functionality of the EsR-based regulatory system in plants rather than to fine-tune it for a specific application. Hence, no rigorous optimization of promoter design for downstream genes was performed, and the promoter architectures used were empirical design choices informed by in silico *analysis and our prior experience of working with these reporter genes.*

3) There are several inducible systems in plants that the authors do not address as alternatives to their system. The dexamethasone-glucocorticoid induction system (PMID: 9107046), the ethanol-inducible system (PMID: 11722766) and the estrogen-inducible system (PMID: 11069700) have been used for years. While each has its limits, I don’t think they should be completely ignored, as the authors have done. The authors need to compare their system to the state of the art to show that there is an improvement.

We have revised the Introduction to include a more comprehensive overview of existing inducible plant expression systems, including the systems highlighted by the Reviewer, and to clarify how the system developed in this study addresses the existing gap.

Chemically-inducible expression tools are valuable academically for studying gene function and regulation, and practically for controlling phenotypes and processes in many organisms[1]. Numerous inducible gene expression systems have been discovered and developed through the generation of inducible chimeric transactivators and extensive protein-engineering efforts to expand the ligand specificities of the allosteric transcription factors (Tang & Cirino, 2011; Taylor et al., 2016; Wang et al., 2020; Rondon & Wilson, 2021). In plants, these efforts have resulted in the development of several widely used inducible systems, including dexamethasone- (Aoyama and Chua, 1997), estrogen- (Zuo, Niu, and Chua, 2000), and ethanol-inducible (Roslan et al., 2021) systems. These systems provide strong and temporally controllable gene activation in laboratory settings. However, their inducers are not suitable for field deployment due to regulatory restrictions (dexamethasone, estrogen) and challenges related to volatility, dosing control, and pleiotropic effects (ethanol).

Important features of a field-compatible system include low background, high magnitude of inducer-evoked response, and practicality of inducer application (*i.e.*, rapid penetration and movement to target tissues/cells, lack of degradation by target plant, neutrality to growth and development, safety, photostability, and registration for field use). Only two existing plant-specific induction systems employ approved agrochemicals as inducer: ecdysone receptor-based systems (Unger et al., 2002, Padidam et al., 2003; Tavva et al., 2006) and ABA receptor PYR1-based systems (Park et al. 2015; Park et al. 2024).

In the case of ABA receptor-based systems, *in planta* reporter gene activation was shown only in response to azinphos-ethyl - a compound banned due to its extreme toxicity. By contrast, the agriculturally relevant candidate from that paper - mandipropamid - was shown only to induce expression of RD29B, a native ABA-responsive gene, with no synthetic reporter gene induction demonstrated.

The ecdysone receptor (EcR)-based systems appeared highly promising in the early 2000s during the period of their active development (Unger et al., 2002, Padidam et al., 2003; Tavva et al., 2006). However, they have never become widely adopted in plants for several reasons. The primary limitation is the poor bioavailability of approved ecdysone analogs (such as tebufenozide and methoxyfenozide) within plant tissue (Carlson et al., 2001), which makes induction inefficient, particularly when applied by spraying. In addition, many potent EcR agonists have been reported to occur naturally in plants (Adler & Grebenok, 1999) (*e.g.*, phytoecdysteroids like ecdysterone, a.k.a. 20-hydroxyecdysone), leading to undesirably high basal expression levels and diminished responses to exogenous inducer. These shortcomings have prevented EcR-based systems from being widely adopted for plant use. Thus, there appear to be no readily deployable agrochemical-inducible expression systems.

4) Second paragraph of the introduction: I would add that the ideal inducible system should have a very low false positive or background activity. This should be demonstrated for plants grown under field-relevant conditions.

Thank you for your suggestion. We have added it:

Important features of a field-compatible system include low background, high magnitude of inducer-evoked response, and practicality of inducer application (*i.e.*, rapid penetration and movement to target tissues/cells, lack of degradation by target plant, neutrality to growth and development, safety, photostability, and registration for field use).

5) Paragraph starting on line 338: It seems worrying that the SUR system does not lead to widespread induction of the reporter in maize and only works when DMSO is added, presumably to facilitate uptake through the cuticle of mature plants. Is spraying plants in the field with an herbicide/DMSO mixture practical or safe? If not, this seems like a big hurdle for the implementation of this system in agriculture.

We agree that this system is unlikely to be used in maize agriculture until we figure out the cause of the observed problem and a safe way to overcome this limitation. However, we have shown that the systems work well in a number of diverse crops in field-like settings.

6) The lack of Arabidopsis responsiveness to Muster herbicide/inducer indicates species-specific efficacy. I would also worry that the responsiveness of the system might therefore be variable depending on the tissue or environmental context, as well. Has this been tested? Also, the authors propose that the Muster chemical is inactive due to it being potentially metabolized in Arabidopsis. What is the evidence to support this?

*The responsiveness of the system is indeed variable and highly dependent on expression strategy, as shown in the manuscript. However, some approaches allow for plant-wide activation (see **Figure 5D**). As for the evidence of potential metabolism in* Arabidopsis*, we did not conduct any experiments to delve deeper into this, so it is just a literature data-driven hypothesis. Detailed investigation of this phenomenon is out of the scope of this manuscript.*

7) Figure 4E: the use of the EsR system for Bt induction is promising, however, the efficacy of this system should be compared to a more traditional constitutive expression system. Quantitative data comparing susceptibility to herbivory using both systems is needed in whole plants. There is a possibility that an inducible system may lead to spatiotemporal variation in Bt toxin expression and therefore resistance may be uneven across the plant body or between plants.

These are great suggestions, but we included the Bt induction experiments just as a proof-of-principle. The experiments proposed will most likely require a lot of optimization, and we believe they fall out of the scope of the current manuscript, which is aimed at giving a general overview of the development and applicability of a new inducible expression system.

8) Line 615: “Hence, this technology has the potential to enable single-season traits and eliminate environmental persistence, addressing many safety concerns of transgenic crops.” The authors have not determined what the persistence of the different herbicides used are in the field and how this affects subsequent crops. Not only should the environmental impact of the herbicide be tested, but whether the residual herbicide leads to spurious induction of the SUR system should be tested. The longevity of the treatment effects should also be tested in crops under field-relevant conditions.

This line in the Discussion section of the manuscript is just our speculation about possible future applications of the system. However, we do not have the capacity to test every possible application. SU herbicides have been used for decades now, and information about their year-to-year environmental persistence is available (both on product labels and in independent studies (e.g., doi: 10.1016/j.chemosphere.2005.01.051, 10.1071/EA04221)).

<u>Minor comments:</u>

Pioneer maize inbred PH184C was transformed using *Agrobacterium* strain LBA4404 THY- carrying the plasmid PHP74334. Transformants were selected on mannose[33], and single-copy T0 events were identified and self-pollinated. Segregating null and homozygous T1 progeny were collected, and leaf discs were excised and vacuum-infiltrated with either Es solution or water control. Treated disks were incubated, and DsRed-Express RNA and protein levels were quantified over a 3-day period. Results clearly showed Es-dependent DsRed-Express protein expression by 24 hours, which steadily increased up to 72 hours (**Figure 3B**). DsRed-Express RNA was detected within 1 hour and started to plateau between 24-72 hours post-treatment (**Figure 3C**). Both DsRed-Express protein and RNA were below the level of detection in untreated transgenic and treated non-transgenic controls.

1) Paragraph starting on line 380: This text describes published work and should be substantially shortened and reference provided.

Unfortunately, there is no paragraph starting on line 380 in the manuscript draft that we have submitted, so we are a little bit confused what the Reviewer is referring to. We will be happy to address the request if the Reviewer could cite the paragraph they are talking about.

2) Figure 4C legend: typo for Murashige and Skoog.

Thank you for pointing this out. We fixed the typo.

Reviewer #2 (Remarks to the Author):

Abstract
lines 29 and 32
"affinity" … binding constants determined, estimated?

We agree that the term "affinity" may imply directly measured binding constants (e.g., K_d), whereas our study reports functional EC_{50} values. To improve accuracy, we have revised the abstract to replace the term "affinity" to avoid implying that we precisely measured thermodynamic parameters. We believe these changes clarify the nature of the measurements while preserving the intended meaning.

Designed variants show low-nanomolar EC_{50} values for ethametsulfuron-methyl (Es, Muster®) or chlorsulfuron (Cs, Glean®) and tightly bind the Tet operator sequence, but only in the absence of corresponding SUs. Crystal structures of both EsR and CsR repressors in complex with their respective SU ligands revealed extensive interactions explaining their strong binding.

lines 37-39
"The new proteins …" are not new, better variants/mutants.

We have corrected the language to be more specific:

The SU repressors also function well in mammalian cell culture and may enable regulation of additional genes in conjunction with TetR.

Major problem:
Manuscript lines 173-174 … You have not used iso-7-chlortetracycline! Also, in Supplement Figure 1 you refer to the structure of TetR/iso-tetracycline complex with PDB entry code 1DU7. The figure shows not the iso-7-Chlortetracycline but the common 7-Chlor-tetracycline. In the PDB the entry code 1DU7 is obsolete and you are forwarded to 2X9D published in https://doi.org/10.1021/jm200332e
2X9D shows the real iso-tetracycline. 1DU7 obviously has been overwritten at least in 2011.
In iso-tetracyclines the rigid tetracycline framework (naphthacene, tetracene) is broken into two connected rigid pieces with much more conformational freedom.
With this confusion the manuscript has to be corrected, and the editor may give my name to the authors to provide some help.
By the way, I think a reference using only the PDB code is not appropriate.

Thank you for catching this mistake! We indeed used 7-chlortetracycline, not iso-7-chlortetracycline. We have corrected this throughout the manuscript (lines 173–174, 180, 697, 698, 702, 730) as well as in the captions of Supplementary Figures S1 and S2.

We are aware that the PDB entry 1DU7 is obsolete. However, the initial rounds of modeling for this project were performed in 2006-2007, prior to its replacement by the current entry 2X9D. For consistency with the original modeling workflow, we chose to retain the use of 1DU7 throughout the manuscript. Unfortunately, to our knowledge, there is no primary publication associated with the original 1DU7 deposition; therefore, we are

currently referencing only the PDB entry. If the reviewers/editor have advice on any other citation we should make, that would be greatly appreciated.

According to other reviewer reports the authors spent substantial efforts to improve the manuscript.

referee #5 major comments 1), page 21 of rebuttal.
The criticism on environmental problems should answered and/or pointed out in the manuscript.

We have revised the Discussion to include a paragraph outlining environmental concerns associated with the potential overuse of SU herbicides if widely utilized as inducers to control traits in crops:

The repressor system developed here can be useful for many applications. This technology can facilitate conditional regulation of beneficial trait genes (as we demonstrated with Cry2A Bt toxin expression), while simultaneously controlling weeds to optimize crop performance. We also showed an increase in transformation efficiency in monocots with inducible *Wuschel2* (*Wus2*) and *Baby boom* (*Bbm*) expression with no deleterious effects on plant growth. With latent gene activation, it may also be possible to unlock high-value traits incompatible with commercial seed production or to maintain dominant male-sterile parents for hybrid seed production. Alongside other inducible expression systems, SURs can be useful for facilitating work with difficult-to-transform species (like monocots), increasing the efficiency of producing marker-free transgenic plants[78], annotating function of genes whose overexpression or silencing prevents recovery of transgenic plants or production of viable seeds[79-81], and other basic plant biology studies.

It is possible that the benefits of the developed induction system could lead to the field application of SU herbicides beyond their current applications in weed control. While this is not ideal from an environmental perspective, for induction purposes SU compounds could be applied at even lower concentrations than those currently used in standard agricultural practice, and they are among the lower-toxicity herbicides. Moreover, this work establishes the feasibility of substantial shifts in substrate specificity, which we expect will ultimately enable the development of switches responsive to even safer, more selective, and/or non-herbicidal inducers in future iterations.

Additionally, the new repressors could find application in many fields of biotechnology and basic research beyond plants. <…>

Reviewer #6 (Remarks to the Author):
The authors have made considerable effort to address the reviewers' comments. While much of their response is satisfactory, a few questions remain, which are highlighted below (listed as 1, 2 and 3).

- Important features of a field-compatible system include low background, high magnitude of inducer-evoked response, and practicality of inducer application (i.e., rapid penetration and movement to target tissues/cells, lack of degradation by target plant, neutrality to growth and development, safety, photostability, and registration for field use). Only two

existing plant-specific induction systems employ approved agrochemicals as inducer: ecdysone receptor-based systems (Unger et al., 2002, Padidam et al., 2003; Tavva et al., 2006) and ABA receptor PYR1-based systems (Park et al. 2015; Park et al. 2024). In the case of ABA receptor-based systems, in planta reporter gene activation was shown only in response to azinphos-ethyl - a compound banned due to its extreme toxicity. By contrast, the agriculturally relevant candidate from that paper - mandipropamid - was shown only to induce expression of RD29B, a native ABA-responsive gene, with no synthetic reporter gene induction demonstrated. The ecdysone receptor (EcR)-based systems appeared highly promising in the early 2000s during the period of their active development (Unger et al., 2002, Padidam et al., 2003; Tavva et al., 2006). However, they have never become widely adopted in plants for several reasons.

In the case of ABA receptor-based systems, in planta reporter gene activation was shown only in response to azinphos-ethyl - a compound banned due to its extreme toxicity

1. These orthogonal gene expression circuits (Park et al, 2024) are indeed intended to report toxic molecules as biosensors for environmental monitoring. Such manuscript does not appear to suggest that azinphos-ethyl should be used for controlling traits in crops. Please update accordingly.

We have revised this paragraph to make it clear that azinphos-ethyl was never suggested by Park et al. *as an inducer to control traits in crops:*

In the case of ABA receptor-based systems, *in planta* reporter gene activation was shown only in response to azinphos-ethyl – to demonstrate the system's potential for environmental monitoring of this extremely toxic compound. By contrast, the agriculturally relevant candidate from that paper - mandipropamid - was shown only to induce expression of *RD29B*, a native ABA-responsive gene, with no synthetic reporter gene induction demonstrated.

- Statistical analyses of several experiments are completely missing (Quantification of every fluorescence panel on Figure 4 and quantification and statistics of Figure 4E; Figure 5F and 5G). We added statistical analysis of Figures 5F and 5G, as requested (see below). For the tobacco leaf disc fluorescence images shown in Figure 4C, the original unmerged image files are sadly no longer available. As a result, we cannot confidently perform quantitative analysis without risking misrepresentation of the data. We therefore prefer to retain these results as qualitative, representative images.

2. It is not clear whether raw images from all replicates are unavailable and whether this situation aligns with the journal's guidelines during peer review.

All nine biological replicates are shown on the panel. Technical replicates were performed at the same time, whose raw images have also been lost to time. To ensure transparency, we have clarified this limitation in the appropriate Methods section (see below). We are happy to provide any additional clarification if required to ensure adherence to the journal's guidelines.

Duplicate leaf disks from rooted imazapyr-resistant tobacco plantlets transformed with either pVER7384 or pVER7385 were placed on MS agar with Es at the indicated

concentration and incubated at 25 ºC for 3 days under 16/8 hr light/dark cycle, during which the samples were inspected daily for DsRed-Express expression. All photos were taken with a 0.5 s exposure and arranged in a 9x9 contact-sheet format using XnView software with brightness set to 9, contrast to 22, and gamma to 2. Original unprocessed image files are no longer available; therefore, these data are presented qualitatively.

- The measuring units at which the herbicides are applied change throughout the paper constantly (ppm, ppb, gr/Ha, etc). This is very confusing when trying to compare across experiments. Select one way of presenting in the main text and be consistent. Also, although the understanding of “billions” in the scientific literature is almost always 1000 millions, in general billions represent different things in different parts of the world (1 million of millions). It would be convenient to use a concentration unit that is understood across the world (molarity).

Thank you for pointing out the potential ambiguity of the term “billions.” We have now converted all concentrations in the manuscript into mass concentrations. Converting these values into molar concentrations would result in quite awkward numbers, which we believe would confuse the readers rather than improve the clarity of the manuscript.

3. It should be easy to prepare a supplementary table listing the tested concentrations using molarity, which is commonly used in scientific publications. The authors should consider whether the journal follows this convention.

We agree that reporting molar concentrations improves clarity. We now provide approximate molar concentrations alongside mass concentrations throughout the main manuscript text.

Reviewer #2 (Remarks to the Author):

According to other reviewer reports and the initial manuscript the authors spent substantial improvement to the manuscript.

To clarify my former issue:
In Figure S1 the 7-chlortetracycline is shown.
The authors did the initial rounds of modelling with PDB entry 1DU7.
I suggest a suitable reference for the 7-chlortetracycline/TetR complex DOI: https://doi.org/10.1006/jmbi.1994.0138 with PDB entry 2TCT by Caroline Kisker et al., The Complex formed between Tetracycline Repressor and Tetracycline-Mg2+ reveals Mechanism of Antibiotic Resistance. J. Mol. Biol. 247 (2), 260-280 (1995).

Using PDB entry 1DU7 is not helpful as this data are not accessible and misleading for the reader because of the entry title: Crystal structure of Tet repressor class D with 4-epi-tetracycline (no publication available).
This is a stereoisomer which would not bind to TetR due to steric clashes.
Therefore, this entry has been obsoleted and superseded by entry 2X9D, because the correct chemical structure of the bound ligand is iso-7-chlortetracycline.
Iso-tetracyclines are degradation products of tetracyclines, see DOI 10.1021/jm200332e with the PDB entry 2X9D titled: Tet repressor (class D) in complex with iso-7-chlortetracycline.

We agree with the Reviewer that the obsolete status of 1DU7, as well as the discrepancy between the entry title and actual ligand identity, may be confusing to readers. To address this, we have revised the Methods section to explicitly clarify the annotation and history of this structure, and to make completely clear what ligand we used for our computational modeling and design.

While alternative 7-chlortetracycline/TetR structures such as 2TCT are appropriate starting models, slight differences in protein and ligand conformations between these structures and 1DU7 may lead to non-equivalent modeling outcomes. Therefore, to ensure reproducibility of the reported results, we retained 1DU7 as the primary structural reference throughout the manuscript. We have also added primary citations for all listed PDB entries, where available.

We hope these revisions resolve the Reviewer's concerns and improve clarity for the reader.

PDB structure 1DU7 was used for redesign of the TetR binding pocket. Although the entry is annotated as containing 4-epi-tetracycline, the ligand in the structure is 7-chlortetracycline. This structure has since been obsoleted and superseded by PDB entry 2X9D[93], which contains iso-7-chlortetracycline, a degradation product of 7-chlortetracycline. Despite its obsolete status, the 1DU7 coordinates remain accessible from the PDB. Alternative 7-chlortetracycline/TetR structures (*e.g.,* 2TCT[94]) may serve as suitable starting points. However, slight differences in protein and ligand conformations may lead to non-equivalent modeling outcomes.

Residues interacting with 7-chlortetracycline, as well as residues that could interact with a somewhat translated and/or rotated Ts molecules, <…>.

Minor point:
Figure 1 (A) Chemical structure of tetracycline (Tc).
This is correct, but for the modelling efforts 7-Chlortetracycline has been used.

The Reviewer is correct that the modeling was performed using 7-chlortetracycline. However, this choice of the ligand was driven by the selection of the most suitable TetR structure available at the time, which contained this Tc analog, rather than by an assumption that 7-chlortetracycline better resembles sulfonylurea (SU) compounds than Tc. Throughout the study, we have worked with tetracycline, anhydrotetracycline, and 7-chlortetracycline. Including all these chemical structures in ***Figure 1*** *would take too much space. Therefore, we chose to present only the Tc structure - the canonical ligand of TetR - which we believe is sufficient to illustrate the structural differences between Tc family compounds and SUs – the main purpose of that figure panel. For these reasons, we would prefer to retain the Tc structure in* ***Figure 1A****. We hope this is acceptable for the reviewer.*

Reviewer #2 (Remarks to the Author):

After all ... o.k., it was a somewhat stressful, also for the authors I think, sorry.
Finally, my only comment refers to line 947 which has a pdf-format depending a strange character after
"identical side-chains truncated to C "

Thank you for pointing this out. The strange character after "identical side-chains truncated to C" is the Greek letter γ. We will make sure it is displayed correctly in the pdf version.

The search model employed was an ensemble of the LBDs of 16 unique dimers of class B or class D TetR structures (PDB IDs: 1A6I, 1BJ0, 1BJZ, 1DU7, 1ORK, 1QPI, 2NS7, 2NS8, 2O7O, 2TCT, 2TRT, 2VKE, 2VPR, and 2VKV) with non-identical side-chains truncated to C_γ or the last common atom.
